# Recoding of stop codons expands the metabolic potential of two novel Asgardarchaeota lineages

**DOI:** 10.1038/s43705-021-00032-0

**Published:** 2021-06-28

**Authors:** Jiarui Sun, Paul N. Evans, Emma J. Gagen, Ben J. Woodcroft, Brian P. Hedlund, Tanja Woyke, Philip Hugenholtz, Christian Rinke

**Affiliations:** 1 Australian Centre for Ecogenomics, School of Chemistry and Molecular Biosciences, The University of Queensland, St Lucia, QLD Australia; 2 School of Earth and Environmental Sciences, The University of Queensland, St Lucia, QLD Australia; 3 Centre for Microbiome Research, School of Biomedical Sciences, Queensland University of Technology (QUT), Translational Research Institute, Woolloongabba, Australia; 4 School of Life Sciences and Nevada Institute of Personalized Medicine, University of Nevada, Las Vegas, NV USA; 5 DOE Joint Genome Institute, Berkeley, CA USA

**Keywords:** Metagenomics, Metagenomics

## Abstract

Asgardarchaeota have been proposed as the closest living relatives to eukaryotes, and a total of 72 metagenome-assembled genomes (MAGs) representing six primary lineages in this archaeal phylum have thus far been described. These organisms are predicted to be fermentative heterotrophs contributing to carbon cycling in sediment ecosystems. Here, we double the genomic catalogue of Asgardarchaeota by obtaining 71 MAGs from a range of habitats around the globe, including the deep subsurface, brackish shallow lakes, and geothermal spring sediments. Phylogenomic inferences followed by taxonomic rank normalisation confirmed previously established Asgardarchaeota classes and revealed four additional lineages, two of which were consistently recovered as monophyletic classes. We therefore propose the names *Candidatus* Sifarchaeia class nov. and *Ca*. Jordarchaeia class nov., derived from the gods Sif and Jord in Norse mythology. Metabolic inference suggests that both classes represent hetero-organotrophic acetogens, which also have the ability to utilise methyl groups such as methylated amines, with acetate as the probable end product in remnants of a methanogen-derived core metabolism. This inferred mode of energy conservation is predicted to be enhanced by genetic code expansions, i.e., stop codon recoding, allowing the incorporation of the rare 21st and 22nd amino acids selenocysteine (Sec) and pyrrolysine (Pyl). We found Sec recoding in Jordarchaeia and all other Asgardarchaeota classes, which likely benefit from increased catalytic activities of Sec-containing enzymes. Pyl recoding, on the other hand, is restricted to Sifarchaeia in the Asgardarchaeota, making it the first reported non-methanogenic archaeal lineage with an inferred complete Pyl machinery, likely providing members of this class with an efficient mechanism for methylamine utilisation. Furthermore, we identified enzymes for the biosynthesis of ester-type lipids, characteristic of bacteria and eukaryotes, in both newly described classes, supporting the hypothesis that mixed ether-ester lipids are a shared feature among Asgardarchaeota.

## Main

The recently described Asgard archaea have been proposed as the closest living prokaryotic relatives to eukaryotes, supporting a two-domain tree of life^1,2^. Six Asgard lineages have been described (although see note added in proof), all of which are named after Norse gods; Lokiarchaeota, Thorarchaeota, Odinarchaeota, Heimdallarchaeota, Helarchaeota, and the recently proposed Gerdarchaeota^1,3–5^. Asgard archaea were introduced as a superphylum^1^. However, a subsequent reclassification, based on taxonomic rank normalisation using relative evolutionary divergence (RED), indicates that this lineage is a phylum for which the name Asgardarchaeota was proposed together with the classes Lokiarchaeia, Thorarchaeia, and Heimdallarchaeia as Latin placeholder names until nomenclature types are designated^6,7^.

The inferred eukaryotic-like nature of the Asgardarchaeota, in particular the encoded plethora of eukaryotic signature proteins (ESPs), spurred initial speculations about possible eukaryotic contamination of the recovered metagenome-assembled genomes (MAGs)^8^. However, these arguments have since been refuted by analysing additional MAGs^9^, and long-read sequencing technologies yielding near-complete MAGs have confirmed that eukaryote-like features are integral to Asgardarchaeota genomes^10^. Furthermore, a recent, decade-long cultivation effort resulted in the first Asgardarchaeota co-culture, *Candidatus* Prometheoarchaeum syntrophicum strain MK-D1, a Lokiarchaeia representative from deep-sea sediments^11^. The authors obtained a closed genome encoding 80 ESPs and presented evidence for the transcription of these genes, supporting not only that Asgardarchaeota genomes are not chimeric assembly artefacts, but also that ESP genes are actively expressed by these archaea.

Insights into the metabolism of Asgardarchaeota based on functions inferred from MAGs, transcriptomics, and experimental data from the Lokiarchaeia culture indicate that members of this phylum are mostly anaerobic fermentative heterotrophs^5,11,12^, although at least one lineage has the potential for short-chain hydrocarbon oxidation^4^. In addition, some Heimdallarchaeia seem to have acquired oxygen-dependent pathways in their recent evolutionary history and were inferred to reduce oxygen or nitrate^13^. Heimdallarchaeia, Thorarchaeia and Lokiarchaeia encode the complete archaeal Wood–Ljungdahl pathway^14^, which could function as an electron sink or operate in reverse to oxidise organic substrates^12^. It was further hypothesised that cofactors reduced by Asgardarchaeota during organic carbon oxidation may be reoxidized by fermentative hydrogen production to fuel a syntrophic relationship with hydrogen- or formate-consuming organisms^12^. Study of Lokiarchaeia co-cultures containing *Ca*. P. syntrophicum MK-D1 confirmed several of these inferred functions. In particular, this archaeon uses small peptides and amino acids while growing syntrophically with a methanogen or a bacterial sulphate reducer through interspecies hydrogen and possibly also formate transfer^11^.

Despite this recent focus on Asgardarchaeota, we have likely only explored a small fraction of the diversity encompassed by this phylum. Microbial community profiling based on small subunit (SSU) rRNA gene sequences suggest that many novel Asgardarchaeota lineages are awaiting genomic discovery^5,14,15^. Here we describe 46 Asgardarchaeota MAGs obtained from coastal, hot spring and deep-sea sediments complemented by 25 MAGs extracted from public metagenomic datasets. This improved genomic sampling enabled us to resolve phylogenomic relationships, extend the rank normalisation analysis, and to propose two new classes, *Ca*. Sifarchaeia (see note added in proof) and *Ca*. Jordarchaeia, both named after Norse Gods. Based on metabolic reconstruction we infer both lineages to be hetero-organotrophic acetogens which make use of genetic recodings to enhance their metabolic capabilities, including the first reported complete archaeal pyrrolysine machinery outside of methanogens.

## Results and discussion

### Sampling sites and community profiling

An in silico SSU rRNA gene survey, based on SILVA (r132)^16^ revealed 99 sites around the globe, predominantly from anoxic marine and freshwater sediments, as potential Asgardarchaeota habitats for metagenomic recovery (Fig S1). Subsequently, we collected samples for shotgun sequencing from sites in Queensland, Australia, with similar characteristics and based on SSU rRNA gene screening discovered Asgardarchaeota at relative abundances of up to 2.7% in anoxic sediments from two brackish lakes at the Sunshine Coast (Fig. S2a–c). We extended our search to deep sea sediments and detected Asgardarchaeota in anoxic cores from the Hikurangi Subduction Margin of the Pacific Ocean with relative abundances reaching 11.6% in core segments 1.5–634.7 m below the seafloor (mbsf), with the highest abundances reported for depths >100 mbsf (Fig. S2d, e). Additionally, we identified two hot spring sediments, from Mammoth Lakes, CA, U.S. and Tengchong, China, as Asgardarchaeota habitats (Fig. S2f, g**)**.

### Genome recovery, phylogenomics and taxonomic rank normalisation

Metagenomic analysis of the selected lake, deep-sea and hot spring sediments yielded a set of 46 Asgardarchaeota MAGs, which were supplemented with 25 MAGs recovered from the NCBI Sequence Read Archive (SRA) (Table S1). Overall, the 71 MAGs have an average estimated completeness of 78.7 ± 15.3% with an estimated contamination of 3.8 ± 2.3% (Table S1). The GC content ranged from 28.8 to 48.4%, and the average genome size was estimated to be ~4 Mbp (Table S1 and Fig. S3).

**Fig. 1:**
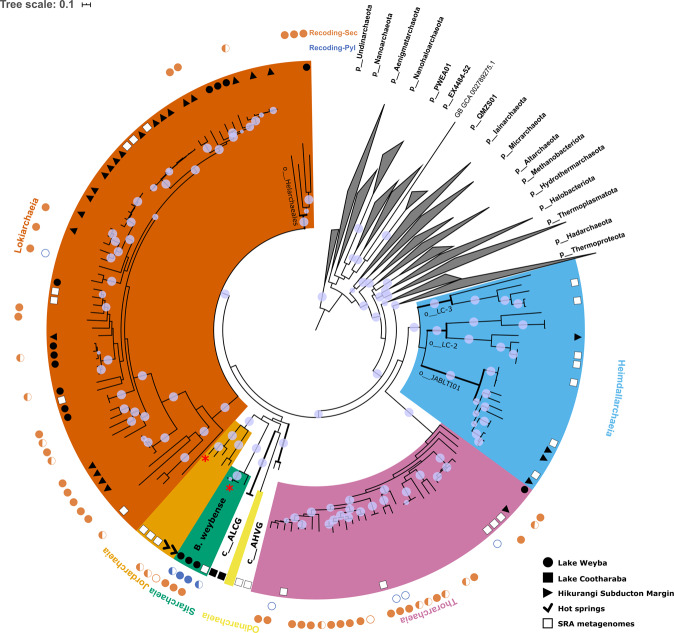
Phylogeny and rank normalised taxonomy of Asgardarchaeota. A maximum-likelihood inference was performed using IQ-TREE under the LG + C10 + F + G + PMSF model, based on a multiple-sequence alignment of up to 122 protein markers (subsampled to 42 amino acids per marker) for 143 Asgardarchaeota MAGs, and 1637 archaeal representatives of non-Asgard lineages in GTDB release r95 (1780 taxa, 5124 sites). The tree was rooted on the Undinarchaeota. Branches with bootstrap support >0.9 are indicated by purple dots. Habitat information for Asgardarchaeota MAGs recovered in this study is shown with black and white symbols at the branch tips corresponding to the symbols in the figure legend. The two outer layers indicate the presence of inferred selenocysteine (Sec, orange) and pyrrolysine (Pyl, blue) encoding systems in each MAG: colour-filled circles represent a complete Sec/Pyl-encoding system, i.e. genes required for the Sec/Pyl biosynthesis and insertion, and the corresponding tRNA; semicircles represent a partial set of detected genes; empty circles indicate the presence of only a tRNAsec or tRNApyl. Asgardarchaeota classes are highlighted in different colours: Bright cyan - Sifarchaeia; dark yellow - Jordarchaeia; Light pink - Thorarchaeia; orange - Lokiarchaeia; Sky blue - Heimdallarchaeia; Light yellow - Odinarchaeia; Asgard hot vent group (AHVG) and Asgard Lake Cootharaba group (ALCG) - black with bold nodes. Note, that the previously proposed lineages ‘Gerdarchaeota’ and ‘Helarchaeota’ are represented by GTDB the order-level lineages Helarchaeales and JABLTI01, respectively.

We inferred evolutionary relationships via maximum-likelihood and Bayesian trees (Table S2) from trimmed multiple-sequence alignments of 122 and 53 archaeal single-copy marker proteins, respectively^17,18^. Our phylogeny was further evaluated by inferring trees from (1) alignments post removal of compositionally biased sites to increase tree accuracy for distantly related sequences, and (2) alignments of alternative concatenated marker sets including 16 ribosomal proteins (rp1)^19^ and 23 ribosomal proteins (rp2)^20^. All phylogenomic inferences of our extended dataset confirmed the monophyly of previously proposed Asgardarchaeota lineages and recovered four novel lineages within this phylum (Fig. 1 and S4–11). Next, we applied the taxonomic rank normalisation approach implemented in the Genome Taxonomy Database (GTDB)^6,7^ to assign ranks to Asgardarchaeota lineages. Our results support the rank of class for Thorarchaeia, Odinarchaeia, Heimdallarchaeia and Lokiarchaeia (Fig. 1 and Table S3). The previously proposed phyla “Helarchaeota” and “Gerdarchaeota” were robustly recovered within the classes Lokiarchaeia and Heimdallarchaeia, and represent the GTDB order-level lineages Helarchaeales and JABLTI01, respectively (Fig. 1 and Table S3). Two of the novel lineages found in the present study comprising 4 and 5 MAGs, were robustly recovered in all phylogenies (Fig. 1 and S4–S11) and were assigned the rank of class based on their RED values and independence from other classes within Asgardarchaeota. A pangenomic analysis based on protein clusters further supported considerable differences between the novel and existing classes (Fig. S12). We propose the names *Ca*. Sifarchaeia class nov. (see note added in proof) and *Ca*. Jordarchaeia class nov., derived from the gods Sif and Jord in Norse mythology. For simplicity, these candidate classes will be referred to as Sifarchaeia and Jordarchaeia in the remainder of the manuscript. We designated type genomes^21^ in both lineages (see proposal of type material) and provide a detailed metabolic reconstruction for both classes below. The phylogenetic placement of the two remaining novel lineages, comprising only two MAGs each, from lake and subsurface sediments, respectively (Table S1 and Fig. S2), was not consistent among trees inferred from different models and marker sets (Fig. S4–11), and we therefore assign them the placeholder names, Asgard hot vent group (AHVG) and Asgard Lake Cootharaba group (ALCG). We foresee that the phylogeny of both lineages will be resolved as more Asgardarchaeota genomes become available.

To evaluate the placement of Sifarchaeia and Jordarchaeia with regard to eukaryotes, we inferred a tree based on 15 markers conserved in the Archaea and eukaryotes^22^. This inference confirmed previous results by placing Heimdallarchaeia as a sister group to Eukarya within Asgardarchaeota, whereas Sifarchaeia and Jordarchaeia clustered with the remaining lineages in this phylum (Fig. S13). The detection of numerous eukaryotic signature proteins (ESPs) in Sifarchaeia and Jordarchaeia (Fig. S14 and Table S4) further supports a close relationship between Asgardarchaeota and eukaryotic organisms. However, the patchy distribution of ESPs in these and other Asgardarchaeota lineages (Fig. S14), and the observed lack of organelle-like structures in the Lokiarchaeia culture^11^, suggests that the ESPs encoded in extant Asgardarchaeota are reminiscent of genes present in the last Asgard archaeal common ancestor (LAsCA) and are likely to perform different functions than their eukaryotic homologues.

### Core metabolism and electron transport

**Fig. 2:**
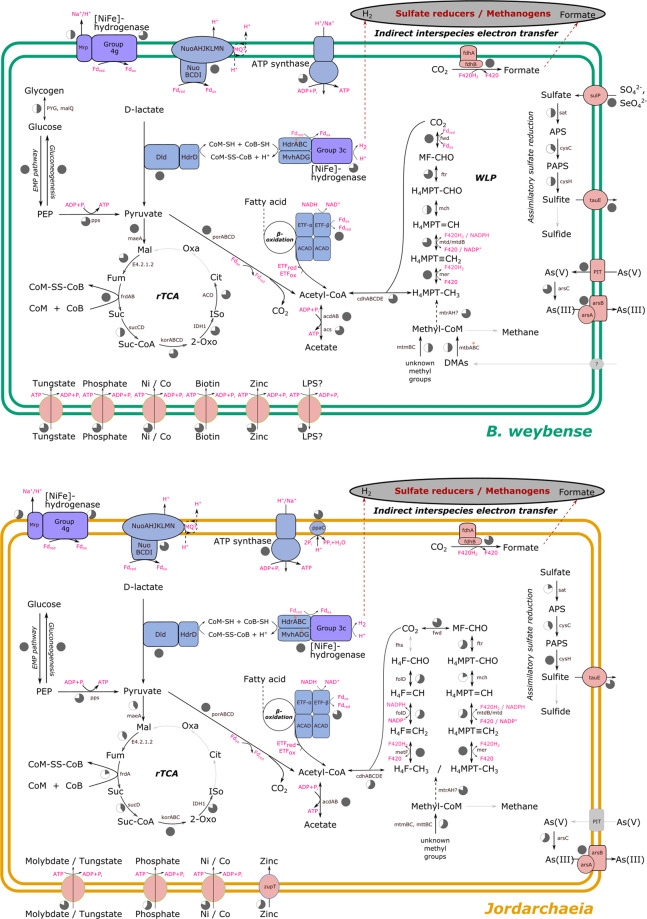
Inferred metabolism of Sifarchaeia and Jordarchaeia. Each of the arrows represents functions assigned to predicted proteins encoded in the respective genomes (Sifarchaeia, 4 MAGs, max. completeness 94.1%; Jordarchaeia, 5 MAGs, max. completeness 95.0%). Pie charts next to the arrows/enzymes indicate the proportion of MAGs that encode a certain enzyme (see Tables S4–S10 for more information). A black arrow indicates that this enzymatic step is encoded by at least one MAG of a given class (Sifarchaeia or Jordarchaeia), and a solid grey arrow indicates an enzymatic step that is missing from a given class. The reactions indicated by black dashed lines are partially encoded and/or are tentative. The dashed red lines represent potential interspecies electron transfer. Note that Sifarchaeia and Jordarchaeia genomes encode an acetyl-CoA synthetase (acs) which preferentially acts in the direction of acetate and ATP production. Abbreviations: APS- adenylyl sulphate, PAPS - 3′-Phosphoadenylyl sulphate, PEP- phosphoenolpyruvate, H4F- tetrahydrofolate, H4MPT- tetrahydromethanopterin, DMA- dimethylamine, MMA- trimethylamine, LPS- lipopolysaccharide, CoM - coenzyme M, Oxa- oxaloacetate, Cit - isocitrate, 2-Oxo - 2-oxo-glutarate, Suc-CoA - succinyl-coenzyme A, Suc -  succinate, Fum - fumarate, Mal - malate, ETF - electron transfer flavoprotein, ETFQO - ETF-ubiquinone oxidoreductase, MQ - menaquinone, Fd - ferredoxin, Pi - inorganic phosphate, PPi - inorganic pyrophosphate, red - redox, rTCA - reverse tricarboxylic acid cycle, WLP - Wood–Ljungdahl pathway.

Based on metabolic inference, we propose that Sifarchaeia and Jordarchaeia are hetero-organotrophs (Fig. 2 and Table S5–S10). This lifestyle is similar to the predicted metabolism of the cultured Lokiarchaeum *Ca*. P. syntrophicum MK-D1, where short-chain fatty acids including acetate are produced via central metabolic pathways^11^. However, unlike  MK-D1 which produces these short-chain fatty acids via the fermentation of amino acids, Sifarchaeia and Jordarchaeia appear to be mostly restricted to oxidising fatty acids or lactate to acetate (Fig. 2).

Fatty acids are likely to be utilised via the canonical β-oxidation pathway predicted in both lineages with acetate and ATP generated through acetyl-CoA synthases (Acd) (Fig. 2 and Suppl. Text). The ability to oxidise fatty acids is common in Archaea such as Archaeoglobus^23^, has been suggested for Asgardarchaeota lineages previously^5,12^, and was recently predicted in an alkane oxidising lineage^4^. Electrons derived from oxidising these fatty acids could establish a membrane potential since Sifarchaeia and Jordarchaeia encode genes for complex I (dehydrogenase) and complex V (ATP synthase) of the electron transport chain (Fig. 2). Notably, complex I lacks the reduced cofactor oxidising subunits NuoEFG, which form the NADH dehydrogenase module. Therefore, we hypothesise that energy conservation in Sifarchaeia and Jordarchaeia depends on electron transfer by reduced ferredoxin (Fig. 2), similar to the membrane-bound fpo-like complex of the acetoclastic methanogens^24^. Electrons from the Nuo complex could be transferred to menaquinone, since Sifarchaeia and Jordarchaeia encode a near-complete biosynthesis pathway for this quinone (Table S9 and Suppl. Text), and subsequently to an unidentified terminal electron acceptor, or alternatively to a membrane-bound hydrogenase for H_2_ generation. The latter has been proposed for syntrophic microorganisms^25^ including the cultured Lokiarchaeia strain MK-D1^11^. However, Sifarchaeia and Jordarchaeia likely use a divergent mechanism since they do not encode the H_2_ producing electron transfer complex FixABCD–HdrABC (Suppl. Text) identified in MK-D1 and other Asgardarchaeota^11^.

Acetate may also be generated from D-lactate by the encoded putative D-lactate dehydrogenase (Dld) via pyruvate oxidoreductase (PorABCD) and acetyl-CoA synthases (Acd) (Figs. 2 and S15). The presence of up to 9 and 13 copies of Dld genes in Sifarchaeia and Jordarchaeia MAGs, respectively, suggest that d-lactate oxidation is important in their metabolism (Table S9). Furthermore, most of these Dld genes are collocated with a heterodisulfide reductase (Hdr) subunit D complex (Fig S15a) that would allow electrons, generated from the lactate oxidation, to reduce coenzyme M (CoM) - coenzyme B (CoB) (Fig. 2 and Suppl. Text). Then a hydrogen evolving NiFe hydrogenase Hdr-Mvh would facilitate the reoxidation of the predicted CoM-CoB heterodisulfide by generating H_2_ and oxidised ferredoxin^11^ encoded in Sifarchaeia and Jordarchaeia MAGs. Alternatively, in Sifarchaeia, both coenzymes might be re-oxidised by the encoded thiol:fumarate reductase which catalyses the reduction of fumarate, with CoB and CoM as electron donors, to succinate and heterodisulfide CoM^26^. The hydrogen produced by this electron-confurcating NiFe hydrogenase Hdr-Mvh complex (Fig. 2), as proposed for strain MK-D1^11^, is likely utilised by Sifarchaeia and Jordarchaeia for indirect interspecies electron transfer. Similarly, both lineages might be able to transfer electrons via formate, catalysed by the encoded formate dehydrogenase (Fig. 2), to syntrophic partners as predicted to occur in the MK-D1 enrichment culture^11,26^. Such a symbiotic relationship would also complement the amino acid and vitamin needs of Sifarchaeia and Jordarchaeia, which lack genes encoding the biosynthesis of the amino acids proline, tyrosine and phenylalanine, and additionally alanine biosynthesis genes are missing in Jordarchaeia (Table S9). Vitamin biosynthesis genes not detected in both novel lineages include biotin, and peridoxin (Table S9).

While these organic acids appear to be key in the metabolism of these novel lineages, members of both classes encode enzymes catalysing the transfer of methyl groups, such as methylated amines, to CoM, similar to a pathway previously reported for methylotrophic methanogens^27^. However, Sifarchaeia and Jordarchaeia are missing genes for methyl-CoM reductase (Mcr), the enzyme catalysing the final step in methane formation. Instead, both novel lineages encode two catalytic subunits of a putative tetrahydromethanopterin (H_4_MPT) coenzyme-M methyltransferase (MtrAH). This predicted two-subunit enzyme differs from the eight-subunit membrane-associated complex in methanogens (Suppl. Text) and has also been reported in Methanomassiliicoccales^28^. The authors of this study proposed that *mtrAH* encodes a H_4_F/H_4_MPT-CoM methyltransferase in these hydrogen dependent methylotrophic methanogens.

Similarly, Sifarchaeia and Jordarchaeia could use this enzyme to catalyse the reverse reaction to facilitate the transfer of methyl groups from methyl-CoM to methyl-H_4_MPT, and subsequently to acetyl-coenzyme A (CoA) to be reduced to acetate for energy conservation (Fig. 2), although the bioenergetics of this potential reaction remain unclear. The putative H_4_MPT-CoM methyltransferase may also oxidise the methyl groups via the reverse archaeal Wood–Ljungdahl pathway (WLP; H_4_MPT-dependent). Alternatively, the WLP could function in the opposite direction to autotrophically fix carbon dioxide using hydrogen as an electron donor, however we did not detect genes of uptake hydrogenases, i.e., Sifarchaeia and Jordarchaeia lack genes for group 1 NiFe-hydrogenases (Table S10).

Besides the possibility for utilising methylamines, genomes of both novel classes encode enzymes to break down complex carbohydrates via glycoside hydrolases including β-galactosidase and α-amylase, and carbohydrate esterases (Table S8). The resulting glucose could be utilised via the Embden–Meyerhof–Parnas (EMP) pathway to generate pyruvate, for subsequent oxidation to acetate, or to be metabolised by the encoded partial reverse TCA cycle (Fig. 2).

### Mixed membrane lipids and the great lipid divide

Both Sifarchaeia and Jordarchaeia encode all genes for the synthesis of archaeal ether-type lipids, but in addition, Sifarchaeia encode enzymes for the biosynthesis of ester-type lipids, characteristic of Bacteria and Eukarya (Fig. S16). This finding aligns with previous reports of ester lipid biosynthetic pathways in Asgard lineages^29^, supporting the hypothesis that mixed ether-ester lipids are a shared feature among Asgardarchaeota. Subsequently, this trait could have been lost in some subordinate lineages, including Jordarchaeia (Fig. S16). Phylogenetic inference of a key ester-type lipid gene supports the finding that archaeal homologues are distinct from their bacterial counterparts^30^ and showed some Lokiarchaeia genes clustering with eukaryotic homologues, albeit with low support values (Fig. S17). The great lipid divide between bacteria and archaea has been further eroded by the discovery of ester-type lipid genes in members of the Poseidoniales (Marine Group II archaea)^31^, and functional validation of ether-type lipid genes in the Fibrobacteres–Chlorobi–Bacteroidetes (FCB) superphylum^32^. This suggests, together with the reported extensive interdomain horizontal gene transfer of several membrane lipid biosynthesis genes^30^, that the lipid divide thought to distinguish the domains of life is more permeable than previously thought.

### Transporters

Sifarchaeia and Jordarchaeia encode several ABC transporters for the uptake of essential trace compounds, including tungstate^33^, which has been shown to enhance the growth of methanogens^34^ and could provide a similar benefit to both classes (Fig. 2, S18 and Table S9). In addition, Sifarchaeia possess a low-affinity inorganic phosphate transporter that also functions as a major uptake system for arsenate^35^. To mitigate the toxicity of arsenate, both classes may be able to actively expel arsenate from their cells by reducing it to the less toxic arsenite^36^, which can then be pumped out of the cell by the ATP-consuming arsenite exporter (Fig. 2).

### Expanding metabolic capabilities by recoding of stop codons

**Fig. 3:**
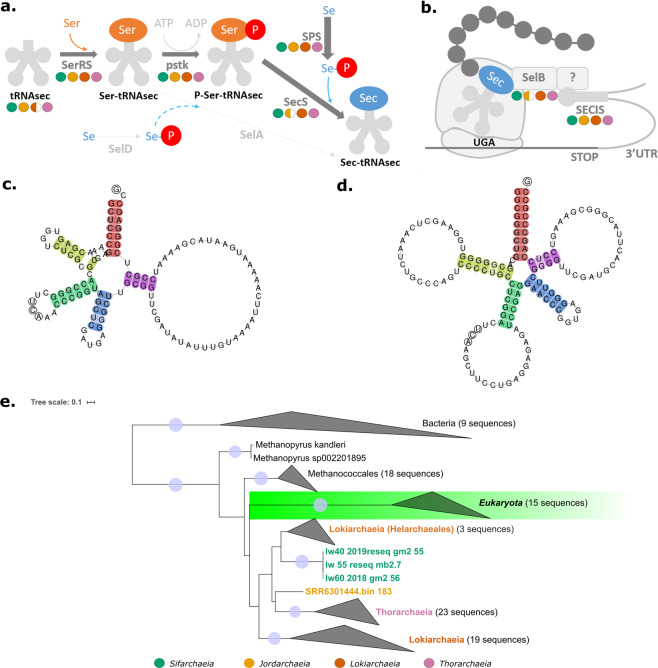
Selenocysteine recoding in Asgardarchaeota. **a** Proposed mechanism of selenocysteine biosynthesis in Asgardarchaeota. **b** Proposed selenocysteine insertion in Asgardarchaeota. Presence of genes in each class are indicated with coloured circles: filled circle – half or more of the MAGs encode the given gene; half-filled circle – less than half of the MAGs encode the given gene. Note that the eukaryotic SECIS-binding protein 2 (SBP2) is missing from all Archaea (indicated by a question mark). **c** tRNAsec in Sifarchaeia MAG “lw60_2018_gm2_56” and **d** tRNAsec in Jordarchaeia MAG “LHC_bin_1308”. Highlighted are the acceptor arm (red), D arm (yellow), anticodon arm (green), variable arm (blue) and the T arm (purple). **e** Phylogenetic tree of *selB*, inferred with IQ-TREE (PMSF C10 model) from a TrimAl-trimmed alignment of SelB genes from archaeal, bacterial and eukaryotic genomes. Tree was rooted between the bacterial and archaeal-eukaryotic clade. Asgardarchaeota sequences are highlighted with different colour labels: bright cyan - Sifarchaeia; dark yellow - Jordarchaeia; light pink - Thorarchaeia and orange - Lokiarchaeia.

Based on inferred proteins and codon usage we predict that Sifarchaeia and Jordarchaeia increase their amino acid synthesis repertoire and consequently their metabolic potential through localised recoding strategies. These include the recoding of the stop codons opal (UGA) and amber (UAG) to incorporate the rare 21st and 22nd amino acids, selenocysteine (Sec) and pyrrolysine (Pyl), respectively, through distinct recoding processes. Both novel classes encode the archaeal/eukaryotic-type Sec biosynthesis machinery (Fig. 3). We also detected a single selenocysteine t-RNA (tRNAsec) in Sifarchaeia and Jordarchaeia MAGs (Fig. 3c, d) and confirmed previous reports of this tRNA in Lokiarchaeia and Thorarchaeia^37,38^, but did not identify a tRNAsec in Heimdallarchaeia or Odinarchaeia (Table S11). Remarkably, the tRNAsec in all Sifarchaeia and some Lokiarchaeia had unusual insertions and deletions, negating previously proposed domain-specific characteristics. For example, the Sifarchaeia tRNAsec has a short 6 bp D-stem **(**Fig. 3 and S19a), a feature that has been attributed to eukaryotes and bacteria, whereas archaeal tRNAsec were thought to generally possess a 7 bp D-stem^39^. Our tRNAsec phylogeny recovered most recoded Asgardarchaeota lineages as monophyletic groups clustering with methanogens and eukaryotes, albeit with low bootstrap support values likely due to the short alignment length (Fig. S19b). The recovery of monophyletic tRNAsec groups that match the species tree suggest that horizontal gene transfers (HGTs) may not be common in the evolutionary history of tRNAsec, despite the reported frequent and extensive gene duplication of tRNAs in general^40^.

Furthermore, Sifarchaeia and Jordarchaeia, as well as Lokiarchaeia and Thorarchaeia, encode enzymes to correctly charge this tRNA in order to synthesise a functional selenocysteine tRNAsec (Sec-tRNAsec) using the archaeal/eukaryotic-type Sec biosynthesis pathway. This process involves an initial mischarging of tRNAsec with serine by seryl-tRNA synthetase, then phosphorylation by phosphoseryl-tRNA kinase and conversion into a functional tRNAsec by Sec synthase using selenophosphate formed by selenophosphate synthetase (SPS) from selenium (Fig. 3a)^41^. We found no evidence for the presence of a bacterial-type Sec biosynthesis pathway in Asgardarchaeota, despite previous reports of a bacterial Sec synthase (SelA) (Fig. 3a) in Thorarchaeia MAG SMTZ1-83^38^. Instead, we suggest that the contig harbouring SelA in this MAG is likely bacterial contamination (Table S12), leading us to posit that Asgardarchaeota rely solely on selenophosphate-dependent synthesis of Sec-tRNASec (Fig. 3a).

Sec insertion in Sifarchaeia and Jordarchaeia and all other Sec recoded Asgardarchaeota lineages could be mediated by the Sec-specific elongation factor (SelB), which connects the selenocysteine insertion sequences (SECIS), an RNA element that forms a stem-loop structure during Sec insertion (Fig. 3b and S20), to the ribosome with the help of the SECIS-binding protein 2 (SBP2). Phylogenetic analysis of SelB and SPS supports a predominantly vertical inheritance of both genes and a separation of bacterial and archaeal/ eukaryotic orthologs (Fig. 3e and S21–S22). Within the archaeal/ eukaryotic branch, the genus *Methanopyrus* was identified as the deepest branching lineage in both trees, and Asgardarchaeota formed a monophyletic sister group to eukaryotes, although with low bootstrap support. We did not detect SBP2 homologues in Asgardarchaeota, consistent with previous reports that Archaea do not encode this elongation factor, and implying that this key enzyme evolved after eukaryogenesis^37,41^. We found 12–25 and 19–25 predicted SECIS elements (the site where Sec insertion occurs) in Sifarchaeia and Jordarchaeia MAGs to facilitate synthesis of two and three selenoproteins in Sifarchaeia and Jordarchaeia repestively (Table S13). The detected selenoproteins were located 30–500 bases upstream of the corresponding SECIS element (Fig. S20a), a distance range previously observed in Archaea and Eukarya^37,42^. All three selenoproteins detected in Sifarchaeia and Jordarchaeia, a heterodisulfide reductase (Hdr) subunit A, a peroxiredoxin (Prx), and a F420-non-reducing hydrogenase iron-sulfur (Mvh) subunit D, are also present in Lokiarchaeia^37^. This suggests that selenoproteins are common to all Asgardarchaeota, which likely depend on the increased catalytic activity of Sec-containing proteins, such as HdrA, as part of their energy conservation strategies (Fig. S2). Indeed, it has been experimentally verified that selenoproteins can provide up to a hundred times increased catalytic activity over cysteine, its sulphur-containing analogue^43^. Furthermore, the selective advantage of selenoenzymes is not restricted to increased efficiency but may also include the ability to function on a broader range of substrates ^44^, and under challenging conditions such as oxidative stress^45^. In the case of Sifarchaeia, Jordarchaeia, and Lokiarchaeia, the Sec-containing protein in the encoded HdrABC-MvhADG-NiFe-hydrogenase complex (Fig. 2) may increase the efficiency of this H_2_ evolving electron-confurcating enzyme complex. Further support for a Sec-enhanced metabolism among Asgardarchaeota are sulphate permeases (SulP), encoded in three Sifarchaeia and several Lokiarchaeia genomes, and predicted to import sulphate and related oxyanions such as selenate, the oxidised form of selenium^46–48^. Subsequently, selenate can be reduced and incorporated into proteins during translation as selenocysteine^49^.

### The first non-methanogenic archaeal Pyl recoding

We detected a second recoding solely present in Sifarchaeia which affects the amber (UAG) stop codon and could allow this class to use the rare 22nd amino acid pyrrolysine (Pyl). The presence of a Pyl tRNA, all required Pyl biosynthesis genes, and specific Pyl-encoded proteins suggests that this recoding provides Sifarchaeia with an efficient mechanism for methylamine utilisation, despite an unusually high UAG stop codon usage.

**Fig. 4:**
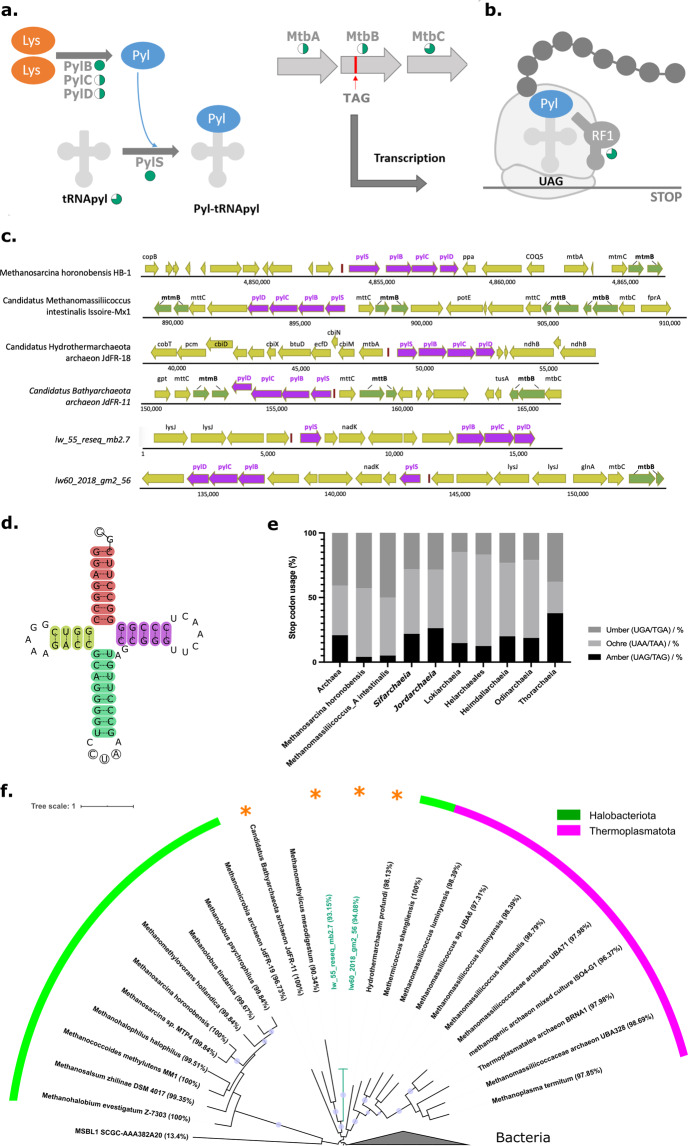
Pyrrolysine recoding machinery and stop codon usage. **a** Proposed pyrrolysine (Pyl) biosynthesis in Sifarchaeia. **b** Proposed Pyl insertion in Sifarchaeia. The proportion of Sifarchaeia MAGs bearing Pyl-recoding machinery genes is indicated with bright cyan pie charts. **c** Gene neighbourhood of the Pyl cluster. Gene names are labelled above the corresponding CDS. Pyl cluster genes (pylSBCD) are highlighted in dark pink, and pyrrolysine-containing genes are highlighted in green. **d** tRNApyl in Sifarchaeia. The highlighted regions are the acceptor arm (red), the D arm (yellow), the CUA anticodon arm (green) and the T arm (purple). The acceptor stem in Sifarchaeia displayed a GC tail, which is distinct from previously reported archaeal and bacterial tRNApyl which have a CCA tail. **e** Stop codon usage in Asgardarchaeota, and two recoded lineages of methanogens. **f** Maximum-likelihood tree (IQtree with 100 bootstraps replicates) based on a concatenated alignment of PylSBCD genes. Purple circles represent branches with bootstrap support over 0.9. The two Sifarchaeia sequences are highlighted with cyan branches and labels. Genome completeness values calculated by CheckM are provided in brackets after each organism name. All taxa for which we report a Pyl cluster for the first time, i.e. *Ca*. Bathyarchaeota archaeon JdFR-11, *Ca*. Hydrothermarchaeum profundi and Sifarchaeia are indicated with an orange asterisk. See Fig. S24 for a rooted PylSBCD tree and Fig. S25–28 for individual gene trees.

Sifarchaeia encode a complete Pyl encoding system including all three Pyl biosynthesis proteins (PylB, PylC, PylD) and a pyrrolysyl-tRNA synthetase (PylS) to charge the pyrrolysine tRNA (tRNApyl, pylT) (Fig. 4a)^50^. Unlike selenocysteine (Fig. 3b), no specific proteins or insertion sequences are required for the tRNApyl insertion, which has been proposed to directly compete with the translation termination release factor for UAG codons (Fig. 4b)^51^. While Pyl genes in Archaea usually form an uninterrupted pylTSBCD cluster, Sifarchaeia show a pattern similar to *Methanohalobium evestigatum*^52^, in which the pylS gene is ~6 Kb distant from pylBCD, separated by a NAD kinase and several hypothetical proteins (Fig. 4c). The tRNApyl of Sifarchaeia, encoded by pylT (Fig. S23), is located upstream of pylS and displays a classic cloverleaf secondary structure with an unusual acceptor stem tail that discriminates the Sifarchaeia tRNApyl from the CCA tails of previously reported archaeal and bacterial homologues (Fig. 4d)^53^. Remarkably, the reported low usage (<6%) of the UAG stop codon in Pyl-containing Archaea^51,54^ does not apply to Sifarchaeia. Instead 21% of their CDSs are terminating with UAG (Fig. 4e and Table S14), a percentage corresponding to UAG frequencies of Pyl-encoding bacteria^55^. How a mis-specification of Pyl-tRNApyl to the frequent UAG stop sense codons is avoided remains unknown, although possible mechanisms exist (see below).

Pyl recoding has only been reported previously in archaeal methanogens belonging to the phyla Thermoplasmatota and Halobacteriota, the class Methanomethylicia (Verstraetearchaeota, *sensu* NCBI taxonomy), and from the candidate lineage *Persephonarchaea MSBL1*^54,56,57^. Thereby, experimental validations of Pyl synthesis have been focused on the genus *Methanosarcina* (Halobacteriota)^58–60^. Several bacterial phyla, including Firmicutes and Desulfobacterota, also possess Pyl recoding thought to be acquired from Archaea via multiple HGTs^61^, but this recoding is absent in eukaryotes^53^. In addition to Sifarchaeia, we identified PylSBCD genes for the first time in Hydrothermarchaeota and Bathyarchaeia representatives by screening GTDB genomes^7^, and most gene phylogenies support a novel cluster containing both representatives, together with Methanomethylicia, and Sifarchaeia (Fig. 4f and S24–S28).

The major role of Pyl recoding in Archaea, methanogenic and non-methanogenic alike, is methylamine utilisation, since Pyl is foremost incorporated in the active sites of methyltransferases^54,62^. Indeed, Sifarchaeia encode several methyltransferases, including monomethylamine methyltransferase (MtmB) and dimethylamine methyltransferase (MtbB), with the latter possessing a Pyl recoding, making it the only in-frame UAG stop codon in Sifarchaeia (Table S15 and Fig. 4c). Thereby, MtbB, together with Methylcobamide:CoM methyltransferase (MtbA), could methylate the cognate corrinoid protein (MtbC), which in turn methylates coenzyme M (CoM)^52^. This cascade of encoded methyl transfers could allow Sifarchaeia to convert methyl groups directly to acetate for energy conservation (see above). Hence, maintaining the Pyl-recoding seems essential, since MtbB requires Pyl, which was hypothesised to activate and orient methylamines as substrates for the corrinoid protein MtbC^52^. How Sifarchaeia, with their high percentage of UAG stop codons, control the specificity of Pyl insertions versus protein termination remains to be determined, however, it has been suggested that environmental conditions such as the presence of methylamines could selectively activate Pyl biosynthesis^51^. Indeed, the Firmicute *Acetohalobium arabaticuma* was recently found to expand its genetic code to include Pyl only in the presence of trimethylamines (TMA), but to down-regulate the transcription of the entire Pyl operon when TMA was absent^55^.

### Recoding evolutionary history in Asgardarchaeota

**Fig. 5:**
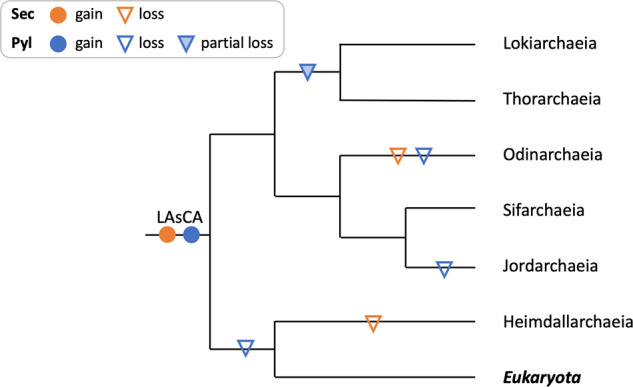
Proposed evolutionary history of pyrrolysine and selenocysteine recoding in Asgardarchaeota. Cladogram, based on the maximum-likelihood tree of 15 ribosomal protein markers (Fig. S13), showing inferred gain and loss of selenocysteine (Sec) and pyrrolysine (Pyl) recoding from the last Asgard archaeal common ancestor (LAsCA) to extant taxa in the Asgardarchaeota including eukaryotes. Partial loss is defined as the loss of Pyl biosynthesis and insertion genes while retaining the Pyl-tRNA.

While the evolutionary history of Pyl encoding is still debated, a structure-based phylogeny suggested that PylS was present in the last universal common ancestor (LUCA)^61,63^. Similarly, it has been argued that Sec recoding is an ancient archaeal trait considering the highly conserved nature of the Sec incorporation machinery^37^, and the fact that the genes involved are not always physically linked in an operon, which impedes its propagation between lineages via horizontal transfer^64^. Our Pyl and Sec trees indicate primarily vertical evolution of these genes (Figs. S20–28), suggesting that HGT is an infrequent event in the evolution of both traits in archaea. Therefore, we suggest that the last Asgardarchaeota common ancestor possessed both the Pyl and Sec recoding. Subsequently, Pyl was lost in the branch leading to Heimdallarchaeia and eukaryotes, and also in Jordarchaeia and Odinarchaeia (Fig. 5). Lokiarchaeia and Thorarchaeia also lack the Pyl gene cluster (Figs. 5 and Fig. 1 and Table S15), but we detected tRNApyl sequences in genomes from both lineages which could be remnants of an ancient Pyl trait that has since been lost. The roles of these tRNAs are unknown, however, they could function as sources of various small noncoding RNA species^65^. Sec recoding, on the other hand, remained present in most Asgardarchaeota lineages and was only lost in Heimdallarchaeia and Odinarchaeia (Fig. 5). Maintaining these presumably ancient recodings could be driven by selective metabolic advantages, i.e. the catalytic advantages of Sec-containing enzymes and the importance of Pyl for active sites of methyltransferases (see above).

### Inferred ecology of novel Asgardarchaeota lineages

The low relative abundances of Asgardarchaeota in our available samples (0.1–2.67%, Fig S2) impeded visualisation and multi-omics approaches, and limited the interpretations of ecological roles of Sifarchaeia and Jordarchaeia to the analysis of physicochemical metadata, taxonomic and functional community profiles, and features inferred from genomic reconstructions.

Based on our chemical analysis, we found that the sulphate levels in lake Weyba sediments, from which Sifarchaeia MAGs were recovered, were higher than in a neighbouring lake (Table S16) and comparable to levels in anoxic deep sea sediments^4,66^, which suggested that this site is a suitable habitat for sulphate reducers. Indeed, we detected dissimilatory sulphite reductase (*dsrAB*) genes, encoding a key enzyme in sulphate reduction^67^, in Lake Weyba metagenome assemblies (Table S17). Furthermore, SSU rRNA gene-based community profiles of Sifarchaeia-containing samples revealed the presence of taxa assigned to sulphate-reducing bacteria (SRB), including Desulfobacterota (formerly Deltaproteobacteria), with a combined relative abundance of up to 31.5% (Table S18). Given that the only cultured Asgardarchaeum *Ca*. Prometheoarchaeum syntrophicum strain MK-D1 has been maintained in co-cultures with a methanogen or with the SRB *Halodesulfovibrio*^11^, it is tempting to speculate that Sifarchaeia form a similar syntrophic relationship with SRB by providing formate and hydrogen equivalents while receiving certain amino acids and vitamins, which this novel lineage cannot synthesise (Table S9). While methanogens were absent from Lake Weyba metagenomes (Table S18), this result is not surprising, since SRB are believed to outcompete methanogens under non-limiting sulphate concentrations due to the increased energetic efficiency in acquiring common substrates over methanogenesis^68^. Nevertheless, we detected a low number of methyl-CoM reductase (*mcr*)-like genes in Lake Weyba metagenomes, which however, were assigned exclusively to Helarchaeales and other Lokiarchaeia lineages. Helarchaeales have been inferred to possess the potential to anaerobically oxidise short-chain hydrocarbons^4^ and are therefore unlikely to represent a hydrogen-consuming, syntrophic partner for Sifarchaeia. In addition, it has been concluded that the absence of a classical membrane-bound hydrogenase in Helarchaeales eliminates the possibility that hydrogen is a major syntrophic electron carrier^4^.

Community profiles of Jordarchaeia-containing samples vary considerably, but all include <0.9% methanogens (Table S17 and S18) compared to a slightly higher percentage (2.5%) of SRB. These results were consistent with a lower number of Mcr genes compared to *dsr* genes in these samples. Nevertheless, members of both groups, methanogens and SRB, could function as a syntrophic partner for Jordarchaeia. Additionally, hydrogenotrophy is pervasive in geothermal systems, particularly among members of the Aquificales and diverse archaea^69^, providing additional potential metabolic partners for thermophilic Jordarchaeia.

## Conclusion

In the present study, we applied taxonomic rank normalisation to genome phylogenies including 71 novel Asgardarchaeota genomes and propose two novel *Candidatus* classes, Sifarchaeia and Jordarchaeia, which have the potential to convert C1 compounds into organic products as methylotrophic acetogens. Thereby, both classes utilise a methanogen-like pathway but do not encode homologues of the key enzyme methyl-CoM reductase (Mcr). This absence, together with the inferred Mcr-like enzymes in Helarchaeales^4^, and our detection of an McrA-like gene in Lokiarchaeia outside the order Helarchaeales (Fig. S29), suggests that pathways for the utilisation of methane and other hydrocarbon gases, or remnants thereof, played an important role in the evolution of Asgardarchaeota. We further reveal recoding as an ancient trait in this phylum, which allows the incorporation of the rare amino acids selenocysteine (Sec) and pyrrolysine (Pyl) into selected proteins, possibly yielding benefits from enhanced catalytic properties of Sec- and Pyl-containing enzymes. Thereby, Pyl, which is restricted to Sifarchaeia (see note added in proof), with remnant tRNAs in Thorarchaeia and Lokiarchaeia, likely supports efficient methylamine utilisation, and possibly represents another relic from a methylotrophic methanogen or methanotrophic ancestor. Next to Desulfobacterota, living as endosymbionts in a gutless marine oligochaete^70^, Sifarchaeia are only the second lineage inferred to encode both, Sec and Pyl containing proteins. Considering that Sifarchaeia and the symbiotic Desulfobacterota were recovered from anaerobic marine sediments, this type of environment may be a hotspot for stop codon recodings.

Our results support previous reports of a close relationship between Asgardarchaeota and eukaryotes, based on phylogenetic inferences, the detection of various encoded eukaryotic signature proteins and of enzymes for the biosynthesis of bacterial/eukaryotic-type ester lipids in Sifarchaeia and Jordarchaeia and other lineages in this phylum. We envision that future recoveries of additional Asgardarchaeota MAGs, in concert with culture-based approaches, will further fuel phylogenomic and metabolic reconstructions and lead to the experimental verification of encoded functions, thereby ultimately shedding more light on the origin of eukaryotes.

### Note added in proof

During the final stages of review of this manuscript, three papers were published that collectively describe seven new Asgard phyla (and a number of subordinate lineages) based on 39 novel MAGs: Hermodarchaeota^71^, Sifarchaeota^72^, Kariarchaeota, Hodarchaeota, Borrarchaeota, Baldrarchaeota and Wukongarchaeota^73^. These genomes have not been included in the analyses presented in our study due to their recent publication, however, an additional phylogenetic inference indicates that one of our new classes is synonymous with Sifarchaeota and Borrarchaeota (Fig. S30). Due to its publication priority, we have used Sifarchaeota as the base name, noting that this lineage represents a class (Sifarchaeia; see proposal of higher ranks) according to rank normalisation, which we use throughout this manuscript. We also propose the intermediate ranks of family and order, and a corrected spelling of the genus *Ca*. Sifarchaeotum^72^. Furthermore, the species represented by MAG “lw60_2018_gm2_56” in our study belongs to the genus *Ca*. Borrarchaeum proposed by Liu et al.^73^, which in turn belongs to the class Sifarchaeia (Fig. S30). We propose the name *Ca*. Borrarchaeum weybense for this species, see proposal of type material. Additionally, we confirmed pyrrolysine recoding in other members of *Ca*. family Borrarchaeaceae, but not in the two other MAGs representing the class Sifarchaeia (Fig. S30) suggesting that this type of recoding has been lost in some members of this class.

### Proposal of type material

#### Candidatus Borrarchaeum weybense

*Candidatus* Borrarchaeum weybense (wey.ben’se. N.L. neut. adj. *weybense* of or pertaining to Lake Weyba, a saltwater lake in Queensland, Australia). Inferred to be a hetero-organotroph with genetic code expansions (recodings) allowing the incorporation of the rare 21st and 22nd amino acids selenocysteine and pyrrolysine. This uncultured lineage is represented by the genome “lw60_2018_gm2_56”, NCBI BioSample SAMN19461863, recovered from Lake Weyba sediments, and defined as high-quality draft MAG^74^ with an estimated completeness of 94.08% and 3.74% contamination, the presence of a 23S, 16S and 5S rRNA gene and 16 tRNAs.

#### Candidatus Jordarchaeum gen. nov

*Candidatus* Jordarchaeum (Jord.ar.chae’um. N.L. neut. n. *archaeum* archaeon; N.L. neut. n. *Jordarchaeum* an archaeon named after Jord, the goddess of the earth in North mytholody). Inferred to be a hetero-organotroph with genetic code expansions, i.e., recoding, allowing the incorporation of the rare 21st amino acid selenocysteine. Type species: *Candidatus* Jordarchaeum madagascariense.

#### Candidatus Jordarchaeum madagascariense

*Candidatus* Jordarchaeum madagascariense (ma.da.ga.scar.i.en’se. N.L. neut. adj. *madagascariense* of or pertaining to Madagascar, an island country in the Indian Ocean). This uncultured lineage is represented by the genome “EB_bin_7”, NCBI BioSample SAMN19461862, recovered from elephant bird fossils in Madagascar, with an estimated completeness of 95.02% and a contamination of 2.41%, the presence of a 23S, 16S and 5S rRNA gene and 6 tRNAs.

### Descriptions of higher taxonomic ranks

Description of ***Candidatus*** **Sifarchaeaceae fam. nov**. *Ca*. Sifarchaeaceae (Sif.ar.chae.ace’ae. N.L. neut. n. *Sifarchaeum*, Candidatus generic name; -*aceae*, ending to designate a family; N.L. fem. pl. n. *Sifarchaeaceae*, the *Sifarchaeum* family). The family is circumscribed based on concatenated protein phylogeny and rank normalisation approach as per Parks et al. Type genus is *Candidatus Sifarchaeum* (Sifarchaeotum (sic)) with the type species *Candidatus Sifarchaeum* subterraneum (Sifarchaeotum subterraneus (sic)) based on the genome “CR_Bin_142”, GenBank assembly accession **GCA_016292335.1**. Inferred to be a hetero-organotroph lineage.

Description of ***Candidatus*** **Jordarchaeaceae fam. nov**. *Ca*. Jordarchaeaceae (Jord.ar.chae. ace’ae. N.L. neut. n. *Jordarchaeum*, Candidatus generic name; -*aceae*, ending to designate a family; N.L. fem. pl. n. *Jordarchaeaceae*, the *Jordarchaeum* family). The family is circumscribed based on concatenated protein phylogeny and rank normalisation approach as per Parks et al. Type genus is *Candidatus* Jordarchaeum. The description is the same as for *Candidatus* Jordarchaeum gen. nov.

Description of *Ca*. ***Candidatus*** **Sifarchaeales ord. nov**. Sifarchaeales (Sif.ar.chae.a’les. N.L. neut. n. *Sifarchaeum*, Candidatus generic name; -*ales*, ending to designate an order; N.L. fem. pl. n. *Sifarchaeales*, the *Sifarchaeum* order). The order is circumscribed based on concatenated protein phylogeny and rank normalisation approach as per Parks et al. Type genus is *Candidatus Sifarchaeum* (Sifarchaeotum (sic)). Inferred to be a hetero-organotroph lineage.

Description of *Ca*. ***Candidatus*** **Jordarchaeales ord. nov**. Jordarchaeales (Jord.ar.chae.a’les. N.L. neut. n. *Jordarchaeum*, Candidatus generic name; -*ales*, ending to designate an order; N.L. fem. pl. n. *Jordarchaeaceae*, the *Jordarchaeum* order). The order is circumscribed based on concatenated protein phylogeny and rank normalisation approach as per Parks et al. Type genus is *Candidatus* Jordarchaeum. The description is the same as for *Candidatus* Jordarchaeum gen. nov

Description of *Ca*. ***Candidatus*** **Sifarchaeia class. nov**. Sifarchaeia (Sif.ar.chae’i.a. N.L. neut. n. *Sifarchaeum*, Candidatus generic name; -*ia*, ending to designate a class; N.L. neut. pl. n. *Sifarchaeia*, the *Sifarchaeum* class). The class is circumscribed based on concatenated protein phylogeny and rank normalisation approach as per Parks et al. Type order is *Candidatus* Sifarchaeales. The description is the same as for *Candidatus* Sifarchaeales ord. nov.

Description of ***Candidatus*** **Jordarchaeia class. nov**. *Ca*. Jordarchaeia (Jord.ar.chae’i.a. N.L. neut. n. *Jordarchaeum*, Candidatus generic name; -*ia*, ending to designate a class; N.L. neut. pl. n. *Jordarchaia*, the *Jordarchaeum* class). The class is circumscribed based on concatenated protein phylogeny and rank normalisation approach as per Parks et al. Type order is *Candidatus* Jordarchaeales. The description is the same as for *Candidatus* **Jordarchaeales ord.nov**.

## Methods

### Small subunit rRNA gene in silico survey

The SSU rRNA gene survey was based on the SILVA SSU database (release 132, Ref NR 99)^16^ (https://www.arb-silva.de/). We extracted the habitat information (field ‘habitat_slv’, ‘isolation_source’ and ‘lat_lon’ in SILVA ARB database) and manually removed habitat entries whose details are duplicated or ambiguous. The remainder of the habitat entries were grouped into seven categories: ‘sediment marine’, ‘sediment freshwater’, ‘sediment other’, ‘microbial mats/biofilms’, ‘soil/permafrost’ and ‘other”.

### Sample collection and DNA extraction

#### Sunshine Coast Lakes sediment

Lake sediment samples from Lake Cootharaba (LC) (−26.28°, 152.99°) and Lake Weyba (LW) (−26.44°, 153.06°) were sampled using sterilised one-metre PVC pipes. LC sediments at depths from 5 cm to 25 cm and LW sediments at depths from 5 cm to 60 cm were sampled in 5 cm intervals in December 2018 and November 2019. Salinity of lake water was recorded using a Seawater Digital Refractometer (Milwaukee, US). Collected sediments were flash frozen in alcohol and dry ice, and delivered to ALS Environmental testing, Brisbane, Australia for chemical analysis. DNA was extracted within four hours of sampling using the PowerSoil DNA Isolation kit (MoBio, USA) following the manufacturer’s protocol.

#### Hikurangi subduction margin sediment

Deep-sea sediment samples of Hikurangi Subduction Margin were sampled by the International Ocean Discovery Program (IODP) Expedition 375 scientists onboard^75^. Sampling holes were drilled at four sites: U1518 (an active fault near the deformation front; sampling depths range from 0 mbsf to 494.90 mbsf), U1519 (the upper plate above the high-slip slow slip event source region; sampling depths range from 0 mbsf to 640.00 mbsf), U1520 (the incoming sedimentary succession in the Hikurangi Trough; sampling depths range from 0 mbsf to 1045.75 mbsf) and U1526 (atop the Tūranganui Knoll Seamount; sampling depths range from 0 m to 83.60 mbsf)^76^. Sediment cores were sub-sampled shipboard using 5 ml syringes, which were stored and shipped on dry ice until they reached the laboratory and were then stored at −80 °C until DNA extraction. To minimise possible contamination, we trimmed off the outer centimetre of each sample and used the inner sediment core for DNA extraction. To optimise DNA extraction for these low biomass samples, 300 mg sediments were first mixed with G2 DNA/RNA Enhancer beads (Ampliqon, Denmark). The subsequent DNA extraction steps were conducted using the PowerSoil DNA Isolation kit (MoBio, USA) following the manufacturer’s protocol.

#### Geothermal spring sediments

Geothermal spring sediments (top 1 cm) were collected from Little Hot Creek, near Mammoth Lakes, CA, USA, from LHC4 (N37°41.436′, W118°50.653′; 81.1 °C; pH = 6.83) and Jinze Pool located in Dientan, Tengchong County, China (N23.44138°, E98.46004°; 78.2 °C; pH = 6.65). Subsamples were stored and shipped on dry ice until they reached the laboratory and were then stored at −80 °C till DNA extraction. DNA was then extracted from freshly thawed sediment samples using the FastDNA™ SPIN Kit for Soil (MP Biomedicals, Santa Ana, CA) following the manufacturer’s protocol. The physicochemical conditions in Little Hot Creek (LHC4) and Jinze Pool are described in detail elsewhere^77,78^.

### Shotgun sequencing

For the Hikurangi Subduction Margin and Sunshine Coast lake samples Illumina Nextera XT libraries were constructed and shotgun sequenced using NextSeq 500/550 High Output v2 2 × 150 bp paired end chemistry. For the geothermal spring sediments Truseq short-insert paired-end libraries were constructed with an average insert size of 270 bp and sequenced on the Illumina HiSeq 2000/2500 1T platform.

### Public data acquisition

Potential Asgardarchaeota containing metagenomes were identified in the NCBI Sequence Read Archive (SRA) using SingleM (https://github.com/wwood/singlem). This software uses single-copy marker genes to search for public metagenomes containing reads that match a bacterial or archaeal lineage of interest. The search for Asgardarchaeota reads yielded matches for seven corresponding study IDs (SRP029382, SRP061771, ERP013176, SRP077065, SRP049601, DRP003377 and SRP098167) in the SRA database (Table S1). Information from all NCBI sequencing runs from each study was collected, but only shotgun metagenomic sequence runs were downloaded for our analysis.

### Small subunit rRNA gene community profiles

To obtain microbial community profiles, we aligned the reads of all shotgun sequenced samples to the SILVA 132_99 database^16^ and classified the reads into operational taxonomic units (OTUs) using CommunityM (https://github.com/dparks1134/CommunityM) under default settings.

### Metagenome assembly, binning and bin dereplication

The raw reads generated from the Sunshine Coast Lake and Hikurangi Subduction Margin sediment DNA were first processed using SeqPrep (https://github.com/jstjohn/SeqPrep) under default settings to merge overlapping paired-end reads and trim adaptors. Pre-processed paired-end reads were then assembled using metaSPAdes genome assembler v3.13.0^79^ with default settings. The raw reads from the Geothermal spring sediments were assembled using ALLPATHS^80^. Reads obtained from SRA were assembly using metaSPAdes with default settings. BamM (http://ecogenomics.github.io/BamM/) was then used to map sequences back to the assemblies. Next, binning was performed with uniteM (https://github.com/dparks1134/unitem) using selected methods (metabat_sensitive, metabat2, maxbin_107, maxbin_40 and groopM) under the default settings. CheckM^81^ was then applied to calculate estimated completeness, contamination as well as strain heterogeneity. For metagenome-assembled genomes (MAGs) binned via multiple binning methods, the average nucleotide identity (ANI) was calculated, and MAG pairs with ANI >99% were de-replicated by keeping the MAG with the highest quality, defined as completeness − 4 * contamination).

### Phylogenomics, rank normalisation and pangenomics

A total of 143 Asgardarchaeota genomes, including MAGs recovered from samples in this study, extracted from public SRA datasets, and downloaded from Genbank^82^ with an estimated quality (completeness − 4 × contamination) over 40% were included in the downstream analysis. The multiple-sequence alignment of selected MAGs was generated using gtdb-tk^83^ based on 122 archaeal-specific marker proteins (Table S2). Maximum likelihood (ML) phylogenies for archaeal genomes were inferred using IQ-Tree 1.6.9^84^ under the LG + C10 + F + G + PMSF model. Statistical support was estimated on a set of 1480 archaeal genomes (including 1377 non-Asgard archaea GTDB species representatives from GTDB release 05-RS95) using 100 bootstraps replicated under the same model (Figs. 1 and S4–5**)**. In addition, ML trees of trimmed alignments, from which we removed compositionally biased sites to increase tree accuracy for distantly related sequences prior to concatenation, using BMGE^85^ or Divvier^86^, were evaluated with the same method (Figs. S6–7).

To further confirm the phylogenetic placement of Asgardarchaeota lineages, three additional ribosomal protein marker sets were used to create alignments: 16 ribosomal proteins defined in Hug et al.^19^, a subset of 23 proteins used by Rinke et al.^20^ and a subset of 53 from the 56 top ranked archaeal marker proteins assessed in Dombrowski et al.^18^. Proteins were aligned to Pfam and TIGRfam HMMs using HMMER 3.1b2 (http://hmmer.org) with default parameters. The alignments were subjected to phylogenomic analysis using IQ-Tree 1.6.9^84^ under the LG + C10 + F + G + PMSF model (Fig. S8–10). Bayesian trees were inferred with Phylobayes^87^ for a subset of 44 genomes (incl. 34 Asgardarchaeota) under the CAT + GTR + G4 model (Fig. S11). Four independent Markov chains were run for ~43,000 generations. After a burn-in of 10%, convergence was achieved for all chains (maxdiff < 0.1). All phylogenetic trees inferred in this study are summarised in Table S2. Trees were viewed and annotated by iTOL^88^.

The ranks of Asgardarchaeota lineages were normalised with the tool PhyloRank (https://github.com/dparks1134/PhyloRank) based on the relative evolutionary divergence (RED) values, as implemented in the Genome Taxonomy Database (GTDB)^6,7^; https://gtdb.ecogenomic.org/). In brief, PhyloRank linearly interpolates the RED values of internal nodes according to lineage-specific rates of evolution under the constraints of the root being defined as zero and the RED of all present taxa being defined as one. To account for the influence of the root placement on RED values PhyloRank roots a tree multiple times, at the midpoint of each phylum with two or more classes. The RED of a taxon is then calculated as the median RED over all these tree rootings, excluding the tree in which the taxon was the outgroup. The RED intervals for each rank were defined as the median RED value ±0.1 to serve as a guide for the normalisation of taxonomic ranks from genus to phylum in GTDB. Taxonomic assignments follow the naming formation and hierarchy of standard taxonomic categories based on their nomenclature types defined by the International Code of Nomenclature of Prokaryotes and recent proposals to amend the Code^89–91^. We also consider recommendations on quality standards for genomes considered as types see Chuvochina et al.^21^ and references therein.

For example, the recent proposal to formalise the rank of phylum under the Code provision^90^ with the addendum by Whitman et al.^91^ defines that phylum names are to be formed by the addition of the suffix –*ota*, such as Asgardarchaeota. A detailed description of the archaeal GTDB taxonomy including nomenclature curation workflows is provided in Rinke et al.^6^.

Pangenomic analysis of selected Asgardarchaeota MAGs was conducted with Anvi’o version 6.2^92^ following its pangenomics workflow with option “–min-occurrence 2”.

To review the evolutionary relationship between Asgardarchaeota and eukaryotes, we used GraftM^93^ for the identification of orthologues of 15 ribosomal proteins used in a previous studies^1,22^. Eukaryotic hits were confirmed according to their NCBI annotation. The collected sequences for each marker gene were aligned with MAFFT v7.455^94^ and concatenated. The concatenated alignment was then trimmed by TrimAl v1.4^95^ with ‘-gappyout’ selection. Maximum-likelihood tree was calculated by IQ-TREE^84^ under ‘LG + C60 + F + G + PMSF’ model. Statistical branch support was calculated using 100 bootstraps under the same model.

### Proposed type material

MAGs proposal as type material were selected considering MIMAG standards^74^ and following the recommended practice for proposing nomenclature type material^21^.

### Metabolic annotation

Genes of all MAGs were predicted using Prokka^96^ with the extensions “-kingdom archaea --metagenome” and annotated with EnrichM (https://github.com/geronimp/enrichM) against KEGG orthologs, EC, CAzy, Pfam and TIGRFAM databases for metabolic reconstruction. Predicted genes in major pathways were confirmed by querying the NCBI non-redundant (nr) protein database. Interpro IPR domains were assigned using InterProScan 5.31^97^.

#### Hydrogenase

We collected [NiFe]-, [FeFe]- and [Fe]- hydrogenase sequences from the study of Greening et al.^98^ to create a Blast database, which was used to query the 143 Asgardarchaeota genomes to search for potential hydrogenase genes. The sequence hits with e-values < 1e-20, scores >100, and sequence identities >30% were then submitted to HydDB^99^ for further identification of hydrogenase subgroups.

#### Lipid membrane biosynthetic genes

KEGG orthologs of ester/ether lipid biosynthesis genes were used to investigate the potential of membrane lipid synthesis in Asgardarchaeota. To calculate the phylogenetic tree of glycerophosphoryl diester phosphodiesterase (UgpQ), we included genes used from a previous study of UgpQ phylogeny^29^. Eukaryotic UgpQ sequences were obtained from UniprotKB (http://www.uniprot.org) based on assignments to PF03009, including only sequences categorised as “Protein Existence [PE]” with the UniprotKB levels “Evidence at protein level” and/or “Evidence at transcript level”. Asgardarchaeota UgpQ homologues were identified with blastp^100^ against KO K01126 by only retaining sequences with a maximum e-value of 1e-30. Collected UgpQ sequences were aligned using HMMER 3.1b2 (http://hmmer.org) against Pfam PF03009.

In addition, as the lipopolysaccharide ABC transporter genes were exclusively detected in Sifarchaeia MAGs, we inferred phylogenetic trees to rule out the possibility of mis-annotation. Sequences of lipopolysaccharide transport system ATP-binding protein (TagH, COG1134) and lipopolysaccharide transport system permease protein (TagG, COG1682) were collected from the NCBI conserved domain database. Collected sequences together with Sifarchaeia hits for each COG were aligned using MAFFT v7.455^94^, respectively. Maximum-likelihood trees of UgpQ, TagH, and TagG were initially inferred by FastTreeMP^101^ with Wag+Gamma model and subsequently with IQtree^84^ under ‘LG + C60 + F + G + PMSF’ model with 100 bootstraps.

#### ESP identification

High-quality Asgardarchaeota genomes (completeness>90%; <10% contamination; *n* = 38) were selected to search for eukaryotic signature proteins (ESPs) listed in the annotation table in Zaremba-Niedzwiedzka et al.^1^ The analysis was limited to high-quality MAGs in order to minimise false negative hits. The resulting information was used to complete the ESP presence/absence table (Table S4). We used Prodigal^102^ for gene prediction and hypothetical genes were annotated by InterProScan 5.31^97^ to screen for ESP homologues with certain IPR domains. As for ESPs denoted by the COG database, we downloaded sequences for each COG entry from the NCBI conserved domain database^103^. The COG sequences were passed to GraftM 0.13.1^93^ to create GraftM packages, which were then used to query Asgardarchaeota genes, with ‘graftM create’ and ‘graftM graft’ functions under default settings, respectively. Hits were further confirmed by blastp^100^ against the NCBI non-redundant protein database (https://blast.ncbi.nlm.nih.gov/Blast.cgi).

#### Selenocysteine encoding system

We used Secmarker 0.4^39^ with the Infernal score threshold of 40 to detect the presence of tRNAsec in the Asgardarchaeota genomes and all archaeal and bacterial GTDB release 04-RS89 genus-dereplicated genomes. The detected tRNAsec sequences were aligned with MAFFT v7.455^94^ and trimmed by a minimum consensus of 40%^104^. Maximum-likelihood tree of tRNAsec was inferred using IQtree with 100 bootstraps under the VM + F + I + G4 model, which was selected by IQ-TREE’s ModelFinder module^84^. Seblastian^105^ with default settings was applied to search for both selenocysteine insertion sequences and selenoproteins in Asgardarchaeota MAGs. The detected selenoproteins were verified by comparing the annotations to the corresponding Prokka-annotated genes with similar positions.

Genes encoding enzymes responsible for selenocysteine biosynthesis and insertion were decided by annotation methods described above. Additionally, as the Thorarchaeota MAG “SMTZ1-83” is the only Asgardarchaeota genome proposed to encode SelA^38^, we blasted the genes present on the contig (LRSK01000263.1) containing selA, using blastp^100^ under NCBI non-redundant protein sequences database. The results are shown in Table S12, and reveal that this contig is most likely a contamination.

Since homologues of genes encoding SelB and SPS have been reported in archaeal, bacterial and eukaryotic genomes^37^ (Mariotti et al.), we hypothesised that these genes might be valuable to better understand the evolution of selenocysteine recoding. Bacterial and eukaryotic SelB and SPS sequences were selected and downloaded from UniprotKB (http://www.uniprot.org) to cover diverse taxonomic groups. Archaeal SelB and SPS sequences were collected from the order Methanococcales, two *Methanopyrus* genomes, and Asgardarchaeota, whose genomes were reported to be tRNAsec-positive. The collected gene sequences were aligned with MAFFT v7.455^94^ and trimmed by TrimAl v1.4^95^ with ‘-automated1’ selection. Maximum-likelihood trees were calculated by IQ-TREE^84^ under ‘LG + C10 + F + G + PMSF’ model with 100 bootstraps.

#### Pyrrolysine encoding system

The presence of tRNApyl in Asgardarchaeota MAGs was determined by Prokka 1.14.6^96^. All genes of tRNApyl containing contigs of Thorarchaeia and Lokiarchaeota MAGs were compared against NCBI nr with blastp^100^ to screen out possible contamination (Table S19).

Genes encoding enzymes responsible for pyrrolysine (Pyl) biosynthesis (PylS, PylB, PylC, PylD) and insertion (RF1) were detected by annotation methods described above. To explore the evolution of the Pyl system, we collected protein sequences of PylSBCD cluster genes (PylSc, PylSn, PylB, PylC, PylD) from the GTDB release 03-RS86 genus-dereplicated genomes. This was achieved by hmmsearch (Sean R. Eddy, http://hmmer.org) against HMM models of TIGR03912 (pyrrolysine--tRNA ligase, N-terminal region), TIGR02367 (pyrrolysine--tRNA ligase, C-terminal region), TIGR03910 (pyrrolysine biosynthesis radical SAM protein), TIGR03909 (pyrrolysine biosynthesis protein PylC), and TIGR03911 (pyrrolysine biosynthesis protein PylD). Homologues of PylB, PylC and PylD that were not located on the same contigs were excluded, since these genes encode enzymes for pyrrolysine biosynthesis, and were only reported to be in close proximity. All genomes with at least two Pyl genes, which equals 50% of the required genes, were included in the downstream analysis. The collected sequences for each gene were aligned with MAFFT v7.455^94^ and trimmed by TrimAl v1.4^95^ with ‘-automated1’ selection. The PylS alignment was created by concatenating sequences of PylSn and PylSc. Maximum-likelihood trees were calculated by IQ-TREE^84^ under ‘LG + C10 + F + G + PMSF′ model with 100 bootstraps. Then we concatenated the above alignments in the order of pylSBCD, with the absence of certain genes represented by gaps. The contaminated alignment was trimmed by TrimAl v1.4^95^ with ‘-gt 0.4’ selection and further trimmed to exclude columns with less than 40% of consensus. Sequences with <80% remaining amino acids were removed, resulting in a final alignment of 62 protein sequences with 1103 columns. A maximum-likelihood tree was calculated with IQ-TREE^84^ under ‘LG + C10 + F + G + PMSF’ model with 100 bootstraps.

To search for Pyl-containing genes, we applied a strategy described previously^106^. In brief, we compared the annotation of each UAG-terminating CDS in all Sifarchaeia MAGs with the annotation of its downstream neighbouring CDS. In cases of matching annotations, both CDS were fused in silico as a unique CDS and predicted as potentially Pyl incorporating.

#### d-lactate dehydrogenases

Gene annotations of the encoded putative d-lactate dehydrogenase (Dld) KEGG orthologs in Sifarchaeia and Jordarchaeia MAGs were verified using Pfam and TIGRfam HMMs (Table S20).

## Supplementary information

Supplementary Tables

Supplementary Figures

Supplementary Text

## Data Availability

The raw reads and genome sequences from the metagenomes described in this study are available at NCBI under multiple BioProjects: PRJNA678545 (Sunshine Coast lakes) and PRJNA678552 (Hikurangi Subduction Margin). Genome sequences assembled and binned from public metagenomes and described in this study are available at NCBI under the BioProject PRJNA678817. All datasets generated and/or analysed during this study, including genome sequences, are available in our data repository at Zenodo.

## References

[1] Zaremba-Niedzwiedzka K, Caceres EF, Saw JH, Bäckström D, Juzokaite L, Vancaester E (2017). Asgard archaea illuminate the origin of eukaryotic cellular complexity. Nature.

[2] Spang A, Eme L, Saw JH, Caceres EF, Zaremba-Niedzwiedzka K, Lombard J (2018). Asgard archaea are the closest prokaryotic relatives of eukaryotes. PLOS Genet.

[3] Spang A, Saw JH, Jørgensen SL, Zaremba-Niedzwiedzka K, Martijn J, Lind AE (2015). Complex archaea that bridge the gap between prokaryotes and eukaryotes. Nature.

[4] Seitz KW, Dombrowski N, Eme L, Spang A, Lombard J, Sieber JR (2019). Asgard archaea capable of anaerobic hydrocarbon cycling. Nat Commun.

[5] Cai, M, Liu Y, Yin X, Zhou Z, Friedrich MW, Richter-Heitmann T, et al. Diverse Asgard archaea including the novel phylum Gerdarchaeota participate in organic matter degradation. Sci. China Life Sci. (2020) 10.1007/s11427-020-1679-1. 10.1007/s11427-020-1679-1 32201928

[6] Rinke, C et al. A standardized archaeal taxonomy for the Genome Taxonomy Database. Nat Microbiol. 2021;1.in press. 10.1038/s41564-021-00918-8 34155373

[7] Parks DH, Chuvochina M, Waite DW, Rinke C, Skarshewski A, Chaumeil PA (2018). A standardized bacterial taxonomy based on genome phylogeny substantially revises the tree of life. Nat Biotechnol.

[8] Cunha VD, Gaia M, Gadelle D, Nasir A, Forterre P (2017). Lokiarchaea are close relatives of Euryarchaeota, not bridging the gap between prokaryotes and eukaryotes. PLoS Genet.

[9] Narrowe AB, Spang A, Stairs CW, Caceres EF, Baker BJ, Miller CS (2018). Complex evolutionary history of translation elongation factor 2 and diphthamide biosynthesis in archaea and parabasalids. Genome Biol Evol.

[10] Caceres, EF et al. Near-complete Lokiarchaeota genomes from complex environmental samples using long and short read metagenomic analyses. bioRxiv. 2019. 10.1101/2019.12.17.879148.

[11] Imachi H, Nobu MK, Nakahara N, Morono Y, Ogawara M, Takaki Y (2020). Isolation of an archaeon at the prokaryote–eukaryote interface. Nature.

[12] Spang, A, Stairs CW, Dombrowski N, Eme L, Lombard J, Caceres EF, et al. Proposal of the reverse flow model for the origin of the eukaryotic cell based on comparative analyses of Asgard archaeal metabolism. Nat Microbiol. 2019;1. 10.1038/s41564-019-0406-9. 10.1038/s41564-019-0406-9 30936488

[13] Bulzu P-A, Andrei AŞ, Salcher MM, Mehrshad M, Inoue K, Kandori H (2019). Casting light on Asgardarchaeota metabolism in a sunlit microoxic niche. Nat Microbiol.

[14] MacLeod F, Kindler GS, Wong HL, Chen R, Burns BP (2019). Asgard archaea: diversity, function, and evolutionary implications in a range of microbiomes. AIMS Microbiol.

[15] Zhang R-Y, Zou B, Yan YW, Jeon CO, Li M, Cai M (2020). Design of targeted primers based on 16S rRNA sequences in meta-transcriptomic datasets and identification of a novel taxonomic group in the Asgard archaea. BMC Microbiol.

[16] Quast C, Pruesse E, Yilmaz P, Gerken J, Schweer T, Yarza P (2013). The SILVA ribosomal RNA gene database project: improved data processing and web-based tools. Nucl. Acids Res..

[17] Parks DH, Rinke C, Chuvochina M, Chaumeil PA, Woodcroft BJ, Evans PN (2017). Recovery of nearly 8,000 metagenome-assembled genomes substantially expands the tree of life. Nat Microbiol.

[18] Dombrowski N, Williams TA, Sun J, Woodcroft BJ, Lee JH, Minh BQ (2020). Undinarchaeota illuminate DPANN phylogeny and the impact of gene transfer on archaeal evolution. Nat Commun.

[19] Hug LA, Baker BJ, Anantharaman K, Brown CT, Probst AJ, Castelle CJ (2016). A new view of the tree of life. Nat Microbiol.

[20] Rinke, C, Schwientek P, Sczyrba A, Ivanova NN, Anderson IJ, Cheng J-F, et al. Insights into the phylogeny and coding potential of microbial dark matter. Nature. 2013;499:431–7. 10.1038/nature12352 23851394

[21] Chuvochina M, Rinke C, Parks DH, Rappé MS, Tyson GW, Yilmaz P (2019). The importance of designating type material for uncultured taxa. Syst Appl Microbiol.

[22] Castelle CJ, Wrighton KC, Thomas BC, Hug LA, Brown CT, Wilkins MJ (2015). Genomic expansion of domain archaea highlights roles for organisms from new phyla in anaerobic carbon cycling. Curr Biol.

[23] Klenk H-P, Clayton RA, Tomb JF, White O, Nelson KE, Ketchum KA (1997). The complete genome sequence of the hyperthermophilic, sulphate-reducing archaeon Archaeoglobus fulgidus. Nature.

[24] Welte C, Deppenmeier U (2011). Membrane-bound electron transport in methanosaeta thermophila▿. J Bacteriol.

[25] Nobu MK, Narihiro T, Hideyuki T, Qiu YL, Sekiguchi Y, Woyke T (2015). The genome of Syntrophorhabdus aromaticivorans strain UI provides new insights for syntrophic aromatic compound metabolism and electron flow. Environ Microbiol.

[26] Nozhevnikova AN, Russkova YI, Litti YV, Parshina SN, Zhuravleva EA, Nikitina AA (2020). Syntrophy and interspecies electron transfer in methanogenic microbial communities. Microbiology.

[27] Lyu Z, Shao N, Akinyemi T, Whitman WB (2018). Methanogenesis. Curr Biol.

[28] Speth, DR & Orphan, VJ. Metabolic marker gene mining provides insight in global mcrA diversity and, coupled with targeted genome reconstruction, sheds further light on metabolic potential of the Methanomassiliicoccales. PeerJ. 2018;6:e5614. 10.7717/peerj.5614 PMC6147122 30245936

[29] Villanueva L, Schouten S, Damsté JSS (2017). Phylogenomic analysis of lipid biosynthetic genes of Archaea shed light on the ‘lipid divide’. Environ Microbiol.

[30] Coleman GA, Pancost RD, Williams TA (2019). Investigating the origins of membrane phospholipid biosynthesis genes using outgroup-free rooting. Genome Biol Evol.

[31] Rinke C, Rubino F, Messer LF, Youssef N, Parks DH, Chuvochina M (2019). A phylogenomic and ecological analysis of the globally abundant Marine Group II archaea (Ca. Poseidoniales ord. nov.). ISME J.

[32] Villanueva, L, Bastiaan von Meijenfeldt FA, Westbye AB, Yadav S, Hopmans EC, Dutilh BE, et al. Bridging the membrane lipid divide: bacteria of the FCB group superphylum have the potential to synthesize archaeal ether lipids. ISME J. 2020:1–15. 10.1038/s41396-020-00772-2. 10.1038/s41396-020-00772-2 PMC7852524 32929208

[33] Kletzin A, Adams MWW (1996). Tungsten in biological systems. FEMS Microbiol Rev.

[34] Dridi B, Khelaifia S, Fardeau M-L, Ollivier B, Drancourt M (2012). Tungsten-enhanced growth of Methanosphaera stadtmanae. BMC Res Notes.

[35] Rosenberg H, Gerdes RG, Chegwidden K (1977). Two systems for the uptake of phosphate in Escherichia coli. J Bacteriol.

[36] Slyemi D, Bonnefoy V (2012). How prokaryotes deal with arsenic†. Environ Microbiol Rep.

[37] Mariotti M, Lobanov AV, Manta B, Santesmasses D, Bofill A, Guigó R (2016). Lokiarchaeota marks the transition between the archaeal and eukaryotic selenocysteine encoding systems. Mol Biol Evol.

[38] Liu Y, Zhou Z, Pan J, Baker BJ, Gu JD, Li M (2018). Comparative genomic inference suggests mixotrophic lifestyle for Thorarchaeota. ISME J.

[39] Santesmasses, D, Mariotti, M & Guigó, R. Computational identification of the selenocysteine tRNA (tRNASec) in genomes. PLoS Comput Biol. 2017;13:e1005383. 10.1371/journal.pcbi.1005383 PMC5330540 28192430

[40] Widmann J, Harris JK, Lozupone C, Wolfson A, Knight R (2010). Stable tRNA-based phylogenies using only 76 nucleotides. RNA.

[41] Rother M, Quitzke V (2018). Selenoprotein synthesis and regulation in Archaea. Biochim Biophys Acta.

[42] Rother M, Resch A, Gardner WL, Whitman WB, Böck A (2001). Heterologous expression of archaeal selenoprotein genes directed by the SECIS element located in the 3′ non-translated region. Mol Microbiol.

[43] Kim H-Y, Fomenko DE, Yoon Y-E, Gladyshev VN (2006). Catalytic advantages provided by selenocysteine in methionine-s-sulfoxide reductases. Biochemistry.

[44] Gromer S, Johansson L, Bauer H, Arscott LD, Rauch S, Ballou DP (2003). Active sites of thioredoxin reductases: why selenoproteins?. Proc Natl Acad Sci USA.

[45] Snider GW, Ruggles E, Khan N, Hondal RJ (2013). Selenocysteine confers resistance to inactivation by oxidation in thioredoxin reductase: comparison of selenium and sulfur enzymes. Biochemistry.

[46] Aguilar-Barajas E, Díaz-Pérez C, Ramírez-Díaz MI, Riveros-Rosas H, Cervantes C (2011). Bacterial transport of sulfate, molybdate, and related oxyanions. Biometals.

[47] Lindblow-Kull C, Kull FJ, Shrift A (1985). Single transporter for sulfate, selenate, and selenite in Escherichia coli K-12. J Bacteriol.

[48] Turner RJ, Weiner JH, Taylor DE (1998). Selenium metabolism in Escherichia coli. Biometals.

[49] Mangiapane E, Pessione A, Pessione E (2014). Selenium and selenoproteins: an overview on different biological systems. Curr Protein Pept Sci.

[50] Blight SK, Larue RC, Mahapatra A, Longstaff DG, Chang E, Zhao G (2004). Direct charging of tRNA CUA with pyrrolysine in vitro and in vivo. Nature.

[51] Zhang Y, Baranov PV, Atkins JF, Gladyshev VN (2005). Pyrrolysine and selenocysteine use dissimilar decoding strategies. J. Biol. Chem..

[52] Gaston MA, Jiang R, Krzycki JA (2011). Functional context, biosynthesis, and genetic encoding of pyrrolysine. Curr Opin Microbiol.

[53] Tharp JM, Ehnbom A, Liu WR (2017). tRNAPyl: Structure, function, and applications. RNA Biol.

[54] Brugère J-F, Atkins JF, O’Toole PW, Borrel G (2018). Pyrrolysine in archaea: a 22nd amino acid encoded through a genetic code expansion. Emerg Top Life Sci.

[55] Prat, L, Heinemann IU, Aerni HR, Rinehart J, O'Donoghue P, Söll D. Carbon source-dependent expansion of the genetic code in bacteria. Proc Natl Acad Sci USA. 2012. 10.1073/pnas.1218613110. 10.1073/pnas.1218613110 PMC3529041 23185002

[56] Vanwonterghem, I, Evans PN, Parks DH, Jensen PD, Woodcroft BJ, Hugenholtz P, et al. Methylotrophic methanogenesis discovered in the archaeal phylum Verstraetearchaeota. Nat Microbiol. 2016;1:16170. 10.1038/nmicrobiol.2016.170 27694807

[57] Guan Y, Haroon MF, Alam I, Ferry JG, Stingl U (2017). Single-cell genomics reveals pyrrolysine-encoding potential in members of uncultivated archaeal candidate division MSBL1. Environ Microbiol Rep.

[58] Mahapatra A, Patel A, Soares JA, Larue RC, Zhang JK, Metcalf WW (2006). Characterization of a Methanosarcina acetivorans mutant unable to translate UAG as pyrrolysine. Mol Microbiol.

[59] Longstaff DG, Larue RC, Faust JE, Mahapatra A, Zhang L, Green-Church KB (2007). A natural genetic code expansion cassette enables transmissible biosynthesis and genetic encoding of pyrrolysine. Proc Natl Acad Sci USA.

[60] Heinemann IU, O'Donoghue P, Madinger C, Benner J, Randau L, Noren CJ (2009). The appearance of pyrrolysine in tRNAHis guanylyltransferase by neutral evolution. Proc Natl Acad Sci USA.

[61] Borrel, G, Gaci N, Peyret P, O'Toole PW, Gribaldo S, Brugère J-F. Unique characteristics of the pyrrolysine system in the 7th order of methanogens: implications for the evolution of a genetic code expansion cassette. Archaea. 2014;2014:374146. 10.1155/2014/374146 PMC3941956 24669202

[62] Krzycki JA (2004). Function of genetically encoded pyrrolysine in corrinoid-dependent methylamine methyltransferases. Curr Opin Chem Biol.

[63] Kavran JM, Gundllapalli S, O'Donoghue P, Englert M, Söll D, Steitz TA (2007). Structure of pyrrolysyl-tRNA synthetase, an archaeal enzyme for genetic code innovation. Proc Natl Aacd Sci USA.

[64] Copeland PR (2005). Making sense of nonsense: the evolution of selenocysteine usage in proteins. Genome Biol.

[65] Rashad S, Niizuma K, Tominaga T (2019). tRNA cleavage: a new insight. Neural Regen Res.

[66] Wang Y, Feng X, Natarajan VP, Xiao X, Wang F (2019). Diverse anaerobic methane- and multi-carbon alkane-metabolizing archaea coexist and show activity in Guaymas Basin hydrothermal sediment. Environ Microbiol.

[67] Dahl C, Kredich NM, Deutzmann R, Trlfper HGY (1993). 1993. Dissimilatory sulphite reductase from Archaeoglobus fulgidus: physico-chemical properties of the enzyme and cloning, sequencing and analysis of the reductase genes. Microbiology.

[68] Dar SA, Kleerebezem R, Stams AJM, Kuenen JG, Muyzer G (2008). Competition and coexistence of sulfate-reducing bacteria, acetogens and methanogens in a lab-scale anaerobic bioreactor as affected by changing substrate to sulfate ratio. Appl Microbiol Biotechnol.

[69] Spear JR, Walker JJ, McCollom TM, Pace NR (2005). Hydrogen and bioenergetics in the Yellowstone geothermal ecosystem. Proc Natl Acad Sci USA.

[70] Zhang Y, Gladyshev VN (2007). High content of proteins containing 21st and 22nd amino acids, selenocysteine and pyrrolysine, in a symbiotic deltaproteobacterium of gutless worm Olavius algarvensis. Nucleic Acids Res.

[71] Zhang J-W, Dong H-P, Hou L-J, Liu Y, Ou Y-F, Zheng Y-L, et al. Newly discovered Asgard archaea Hermodarchaeota potentially degrade alkanes and aromatics via alkyl/benzyl-succinate synthase and benzoyl-CoA pathway. ISME J. 2021:1–18. 10.1038/s41396-020-00890-x. 10.1038/s41396-020-00890-x PMC8163825 33452484

[72] Farag IF, Zhao R & Biddle JF. “Sifarchaeota” a novel Asgard phylum from Costa Rica sediment capable of polysaccharide degradation and anaerobic methylotrophy. Appl Environ Microbiol. 2021. 10.1128/AEM.02584-20. 10.1128/AEM.02584-20 PMC8091018 33608286

[73] Liu, Y, Makarova KS, Huang W-C, Wolf YI, Nikolskaya AN, Zhang X, et al. Expanded diversity of Asgard archaea and their relationships with eukaryotes. Nature. 2021:1–5. 10.1038/s41586-021-03494-3. 10.1038/s41586-021-03494-3 PMC11165668 33911286

[74] Bowers RM, Kyrpides NC, Stepanauskas R, Harmon-Smith M, Doud D, Reddy T (2017). Minimum information about a single amplified genome (MISAG) and a metagenome-assembled genome (MIMAG) of bacteria and archaea. Nat Biotechnol.

[75] Scientists, E. International ocean discovery program expedition 375 preliminary report: Hikurangi subduction margin coring and observatories unlocking the secrets of slow slip through drilling to sample and monitor the forearc and subducting plate. Integrated Ocean Drilling Program: Preliminary Reports 1–38 (2018) 10.14379/iodp.pr.375.2018.

[76] Wallace, LM, Saffer, DM, Barnes, PM, Pecher, IA, Petronotis, KE, LeVay, LJ Hikurangi subduction margin coring, logging, and observatories. Proceedings of the International Ocean Discovery Program, **372B/375**, (2019).

[77] Vick TJ, Dodsworth JA, Costa KC, Shock EL, Hedlund BP (2010). Microbiology and geochemistry of Little Hot Creek, a hot spring environment in the Long Valley Caldera. Geobiology.

[78] Hou W, Wang S, Dong H, Jiang H, Briggs BR, Peacock JP (2013). A Comprehensive Census of Microbial Diversity in Hot Springs of Tengchong, Yunnan Province China Using 16S rRNA Gene Pyrosequencing. PLOS ONE.

[79] Nurk S, Meleshko D, Korobeynikov A, Pevzner PA (2017). metaSPAdes: a new versatile metagenomic assembler. Genome Res.

[80] Butler J, MacCallum I, Kleber M, Shlyakhter IA, Belmonte MK, Lander ES (2008). ALLPATHS: De novo assembly of whole-genome shotgun microreads. Genome Res.

[81] Parks DH, Imelfort M, Skennerton CT, Hugenholtz P, Tyson GW (2015). CheckM: assessing the quality of microbial genomes recovered from isolates, single cells, and metagenomes. Genome Res.

[82] Benson DA, Cavanaugh M, Clark K, Karsch-Mizrachi I, Lipman DJ, Ostell J (2017). GenBank. Nucleic Acids Res.

[83] Chaumeil, P-A, Mussig, AJ, Hugenholtz, P & Parks, DH GTDB-Tk: a toolkit to classify genomes with the Genome Taxonomy Database. *Bioinformatics* (2019) 10.1093/bioinformatics/btz848. 10.1093/bioinformatics/btz848 PMC7703759 31730192

[84] Nguyen L-T, Schmidt HA, von Haeseler A, Minh BQ (2015). IQ-TREE: A Fast and Effective Stochastic Algorithm for Estimating Maximum-Likelihood Phylogenies. Mol Biol Evol.

[85] Criscuolo A, Gribaldo S (2010). BMGE (Block Mapping and Gathering with Entropy): a new software for selection of phylogenetic informative regions from multiple sequence alignments. BMC Evolutionary Biology.

[86] Ali, RH, Bogusz, M & Whelan, S Identifying clusters of high confidence homologies in multiple sequence alignments. *Mol Biol Evol*10.1093/molbev/msz142. 10.1093/molbev/msz142 PMC6933875 31209473

[87] Lartillot N, Philippe H (2004). A Bayesian Mixture Model for Across-Site Heterogeneities in the Amino-Acid Replacement Process. Mol Biol Evol.

[88] Letunic I, Bork P (2011). Interactive Tree Of Life v2: online annotation and display of phylogenetic trees made easy. Nucleic Acids Research.

[89] Whitman WB (2016). Modest proposals to expand the type material for naming of prokaryotes. Int. J. Syst. Evol. Microbiol..

[90] Oren A, da Costa MS, Garrity GM, Rainey FA, Rosselló-Móra R, Schink B (2015). Proposal to include the rank of phylum in the International Code of Nomenclature of Prokaryotes. International Journal of Systematic and Evolutionary Microbiology.

[91] Whitman WB, Oren A, Chuvochina M, da Costa MS, Garrity GM, Rainey FA (2018). Proposal of the suffix –ota to denote phyla. Addendum to ‘Proposal to include the rank of phylum in the International Code of Nomenclature of Prokaryotes’. International Journal of Systematic and Evolutionary Microbiology.

[92] Delmont TO, Eren AM (2018). Linking pangenomes and metagenomes: the Prochlorococcus metapangenome. PeerJ.

[93] Boyd JA, Woodcroft BJ, Tyson GW (2018). GraftM: a tool for scalable, phylogenetically informed classification of genes within metagenomes. Nucleic Acids Res.

[94] Katoh K, Standley DM (2013). MAFFT Multiple Sequence Alignment Software Version 7: Improvements in Performance and Usability. Mol Biol Evol.

[95] Capella-Gutiérrez S, Silla-Martínez JM, Gabaldón T (2009). trimAl: a tool for automated alignment trimming in large-scale phylogenetic analyses. Bioinformatics.

[96] Seemann T (2014). Prokka: rapid prokaryotic genome annotation. Bioinformatics.

[97] Jones P, Binns D, Chang HY, Fraser M, Li W, McAnulla C (2014). InterProScan 5: genome-scale protein function classification. Bioinformatics.

[98] Greening C, Biswas A, Carere CR, Jackson CJ, Taylor MC, Stott MB (2016). Genomic and metagenomic surveys of hydrogenase distribution indicate H2 is a widely utilised energy source for microbial growth and survival. ISME J.

[99] Søndergaard D, Pedersen CNS, Greening C (2016). HydDB: A web tool for hydrogenase classification and analysis. Scientific Reports.

[100] Altschul SF, Madden TL, Schäffer AA, Zhang J, Zhang Z, Miller W (1997). Gapped BLAST and PSI-BLAST: a new generation of protein database search programs. Nucleic Acids Res.

[101] Price MN, Dehal PS, Arkin AP (2010). FastTree 2 – Approximately Maximum-Likelihood Trees for Large Alignments. PLoS ONE.

[102] Hyatt D, Chen GL, Locascio PF, Land ML, Larimer FW, Hauser LJ (2010). Prodigal: prokaryotic gene recognition and translation initiation site identification. BMC Bioinformatics.

[103] Tatusov RL, Galperin MY, Natale DA, Koonin EV (2000). The COG database: a tool for genome-scale analysis of protein functions and evolution. Nucleic Acids Res.

[104] Ludwig W, Strunk O, Westram R, Richter L, Meier H, Yadhukumar (2004). ARB: a software environment for sequence data. Nucleic Acids Research.

[105] Mariotti M, Lobanov AV, Guigo R, Gladyshev VN (2013). SECISearch3 and Seblastian: new tools for prediction of SECIS elements and selenoproteins. Nucleic Acids Res.

[106] Borrel G, Parisot N, Harris HM, Peyretaillade E, Gaci N, Tottey W (2014). Comparative genomics highlights the unique biology of Methanomassiliicoccales, a Thermoplasmatales-related seventh order of methanogenic archaea that encodes pyrrolysine. BMC Genomics.

