# Correction: Recoding of stop codons expands the metabolic potential of two novel Asgardarchaeota

**DOI:** 10.1038/s43705-021-00048-6

**Published:** 2022-01-24

**Authors:** Jiarui Sun, Paul N. Evans, Emma J. Gagen, Ben J. Woodcroft, Brian P. Hedlund, Tanja Woyke, Philip Hugenholtz, Christian Rinke

**Affiliations:** 1 Australian Centre for Ecogenomics, School of Chemistry and Molecular Biosciences, The University of Queensland, St Lucia, QLD Australia; 2 School of Earth and Environmental Sciences, The University of Queensland, St Lucia, QLD Australia; 3 Centre for Microbiome Research, School of Biomedical Sciences, Queensland University of Technology (QUT), Translational Research Institute, Woolloongabba, Australia; 4 School of Life Sciences and Nevada Institute of Personalized Medicine, University of Nevada, Las Vegas, NV USA; 5 DOE Joint Genome Institute, Berkeley, CA USA

**Keywords:** Metagenomics, Metagenomics

Correction to: *ISME Communications* 10.1038/s43705-021-00032-0, published online 28 June 2021

The original version of this article unfortunately contained mistakes. There was a mix-up among the ORCIDs and the corresponding authors

The original article has been corrected.

