# Supplementary material for: Recoding of stop codons expands the metabolic potential of two novel Asgardarchaeota lineages
Source: ISME Commun. 2021 Jun 28;1:30. doi: 10.1038/s43705-021-00032-0 (PMC9723677; doi:10.1038/s43705-021-00032-0)
Supplement: Supplementary file 2 — Supplementary Figures [file 43705_2021_32_MOESM2_ESM.docx]

**Supplementary Figures**

**
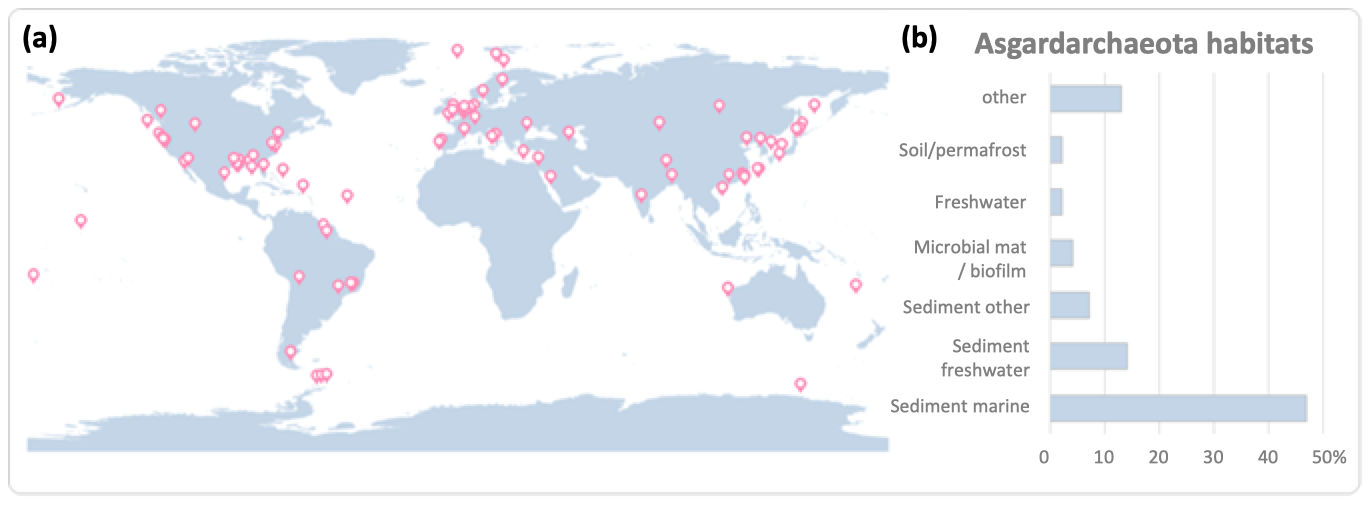
**

**Fig. S1 | Global survey of Asgardarchaeota habitats. (a)** Our *in silico* SSU rRNA gene survey of public data (SILVA release 132, Ref NR 99) revealed 99 habitats around the globe that contained Asgardarchaeota sequences and hence qualified as potential mining sites for the metagenomic recovery of novel lineages. **(b)** Asgardarchaeota habitat types. Most 16S rRNA gene sequences assigned to Asgardarchaeota were detected in marine and freshwater sediment.


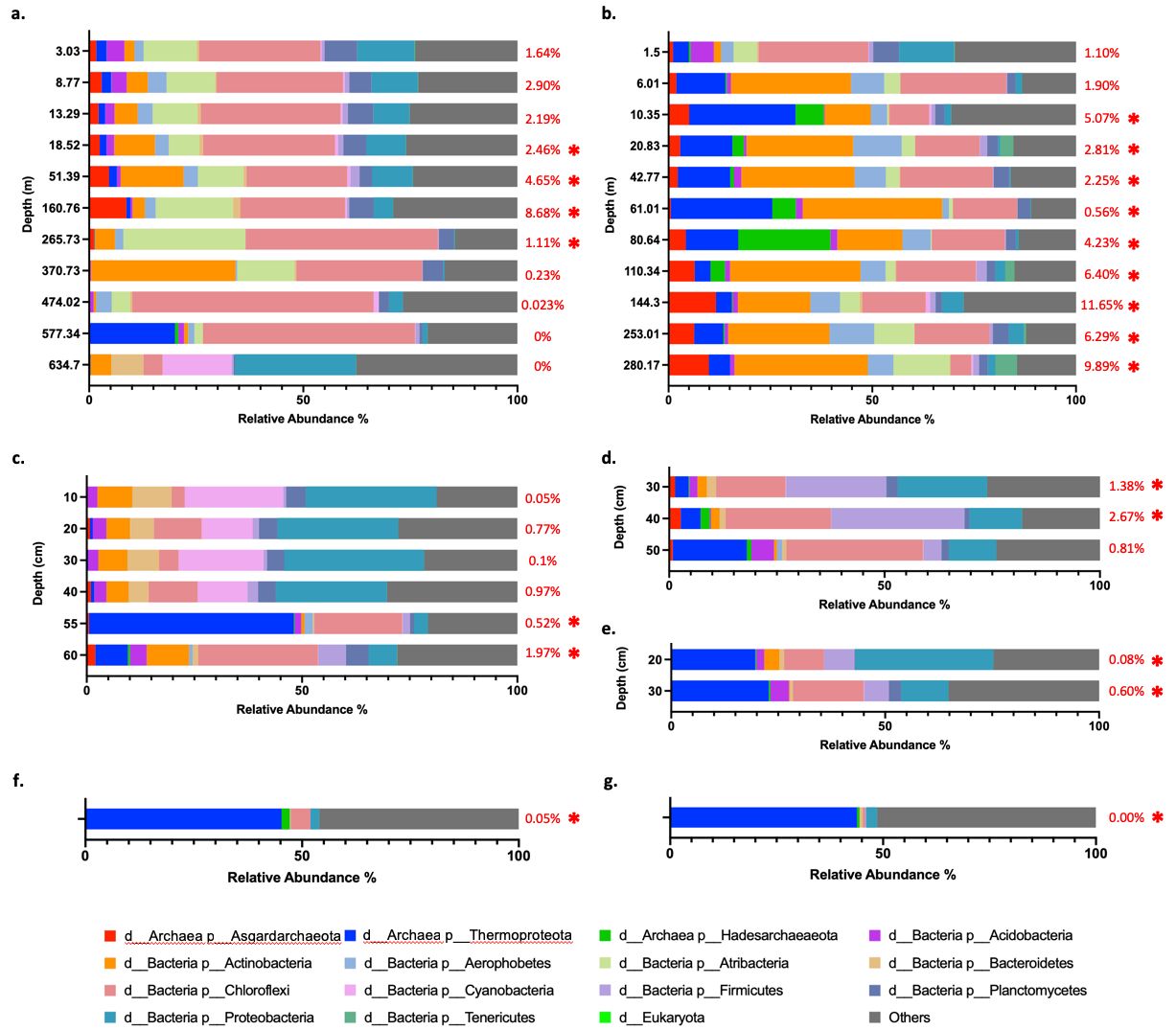


**Fig. S2 | SSU rRNA gene amplicon-based community profiles of Asgardarchaeota sampling sites.** (a) Hikurangi core U1519, (b) Hikurangi core U1520, (c) Lake Weba core2018 (d) Lake Weyba core2019, (e). Lake Cootharaba core2019, (f) Little Hot Creek sediment, (g) Jinze hot spring sediment. There is no depth information for Little Hot Creek and Jinze hot spring sediment. Relative abundances of Asgardarchaeota are provided to the right of each community profile in red font. Samples from which Asgardarchaeota MAGs were recovered are labelled with a red asterisk.


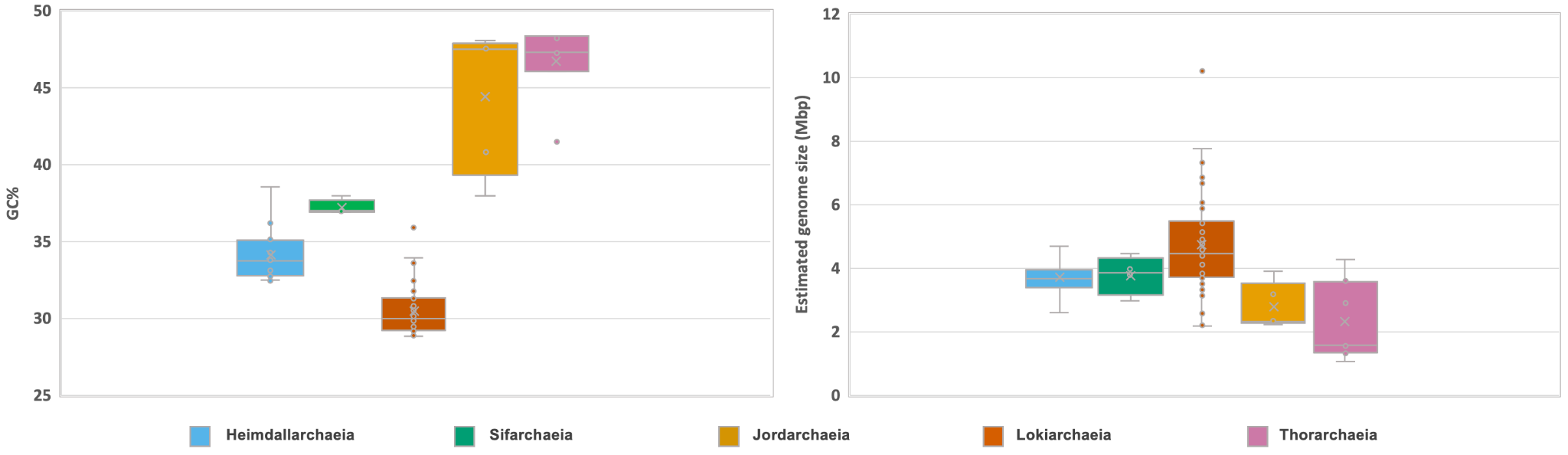


**Fig. S3 | GC content and estimated genome size of Asgardarchaeota MAGs. (a)** %GC content of the MAGs recovered in this study, assigned to the 5 Asgardarchaeota classes. **(b)** Estimated genome size of the MAGs, assigned to Asgardarchaeota classes.

**
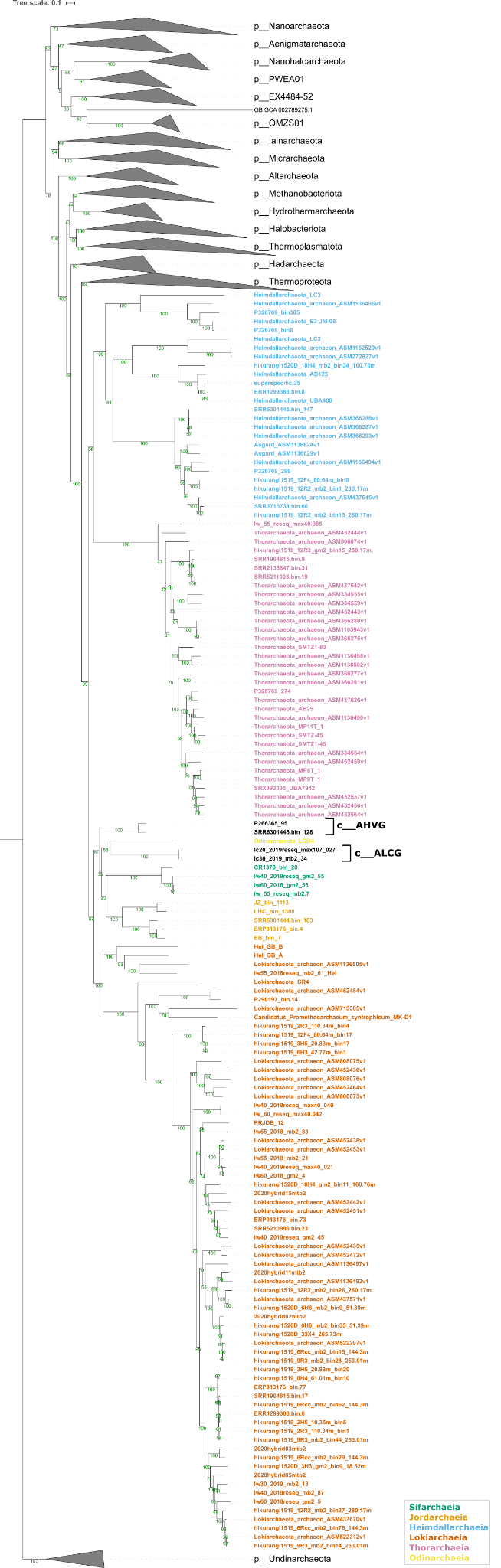
**

**Fig. S4 | Phylogenomic tree of 122 protein markers against GTDB release r95.** The alignment was based on a concatenated set of 122 protein markers (subsampled to 42 sites each, resulting in a total alignment length of 5124 sites) from 1780 taxa. These taxa encompass 143 Asgardarchaeota MAGs, including 71 recovered in this study, and 1377 non-Asgard archaea species representatives from GTDB release 05-RS95. Maximum-likelihood analysis was performed using IQ-TREE under the LG+C10+F+G+PMSF model. The tree is rooted on the Undinarchaeota. Numbers at branches indicate bootstrap statistical support (100 replicates). Asgardarchaeota classes are indicated with different color labels: Bright cyan - Sifarchaeia; dark yellow - Jordarchaeia; Light pink - Thorarchaeia; orange - Lokiarchaeia; Sky blue - Heimdallarchaeia; Light yellow - Odinarchaeia. Tree corresponds to the file “1.3_v3.0_143x_r95_100xBT_PMSFc10.tree”, details are shown in Table S2.


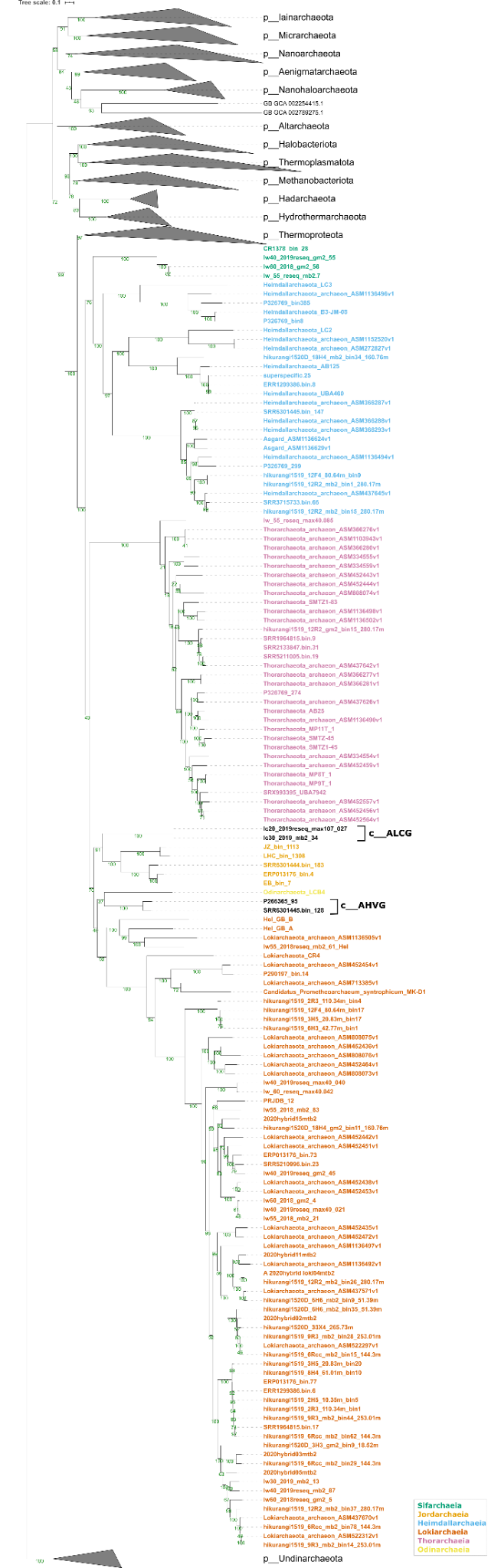


**Fig. S5 | Phylogenomic tree of 122 protein markers against GTDB r89 taxa.** The alignment was based on a concatenated set of 122 protein markers (subsampled to 42 sites each, resulting in a total alignment length of 5124 sites) from 1378 taxa. These taxa encompass 143 Asgardarchaeota MAGs, including 71 recovered in this study, and 1234 non-Asgard archaea species representatives from GTDB release 04-RS89. Maximum-likelihood analysis was performed using IQ-TREE under the LG+C10+F+G+PMSF model. The tree is rooted on the Undinarchaeota. Numbers at branches indicate bootstrap statistical support (100 replicates). Asgardarchaeota classes are indicated with different color labels: Bright cyan - Sifarchaeia; dark yellow - Jordarchaeia; Light pink - Thorarchaeia; orange - Lokiarchaeia; Sky blue - Heimdallarchaeia; Light yellow - Odinarchaeia. Tree corresponds to the file “2.3_v3.0_143x_r89_100xBT_PMSFc10.tree”, details are shown in Table S2.


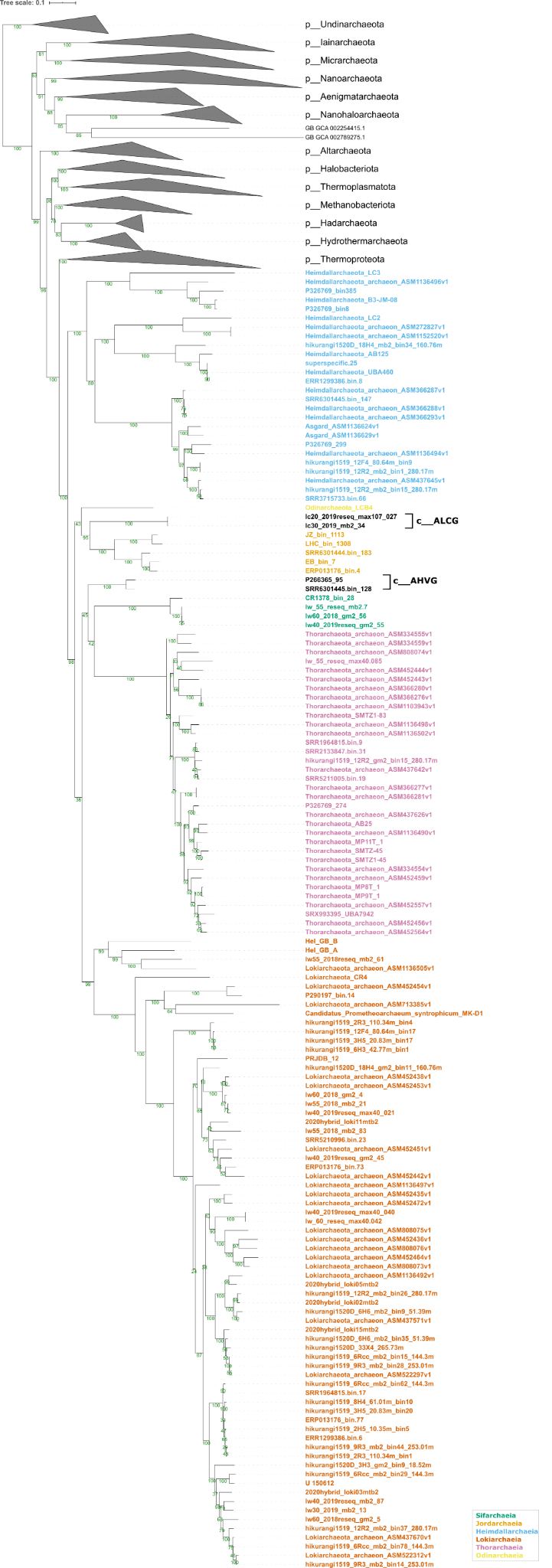


**Fig. S6 | Phylogenomic tree of 122 bmge-trimmed protein markers against GTDB r89 taxa.** The alignment was based on a concatenated set of 122 protein markers with compositionally biased sites removed using bmge (settings: -s FAST -h 0.55) for 143 Asgardarchaeota MAGs and archaeal representatives of all non-Asgard species in GTDB release r89 (1377 taxa, 7529 sites). Maximum-likelihood analysis was performed using IQ-TREE under the LG+C10+F+G+PMSF model. The tree is rooted on the Undinarchaeota. Numbers at branches indicate bootstrap statistical support (100 replicates). Asgardarchaeota classes are indicated with different color labels: Bright cyan - Sifarchaeia; dark yellow - Jordarchaeia; Light pink - Thorarchaeia; orange - Lokiarchaeia; Sky blue - Heimdallarchaeia; Light yellow - Odinarchaeia. Tree corresponds to the file “4.3_143x_r89_bmge_unfiltered_PMSFc10_100xBT.tree”, details are shown in Table S2.

**
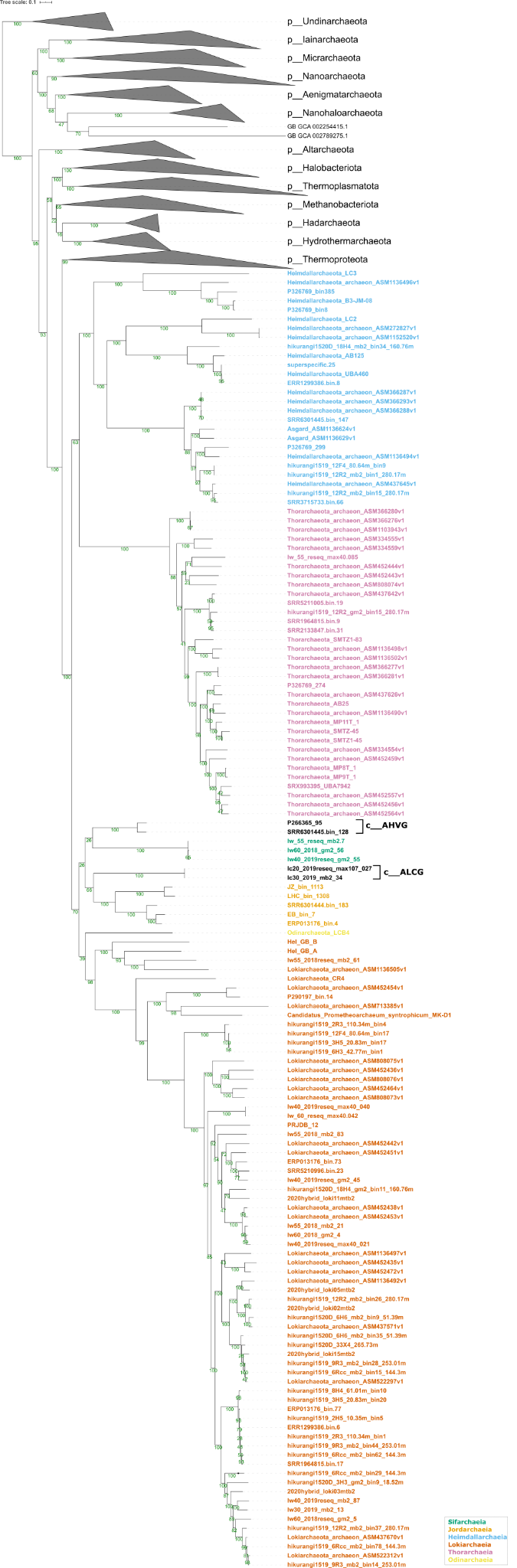
**

**Fig. S7 | Phylogenomic tree of 122 divvier-trimmed protein markers against GTDB r89 taxa.** The alignment was based on a concatenated set of 122 protein markers with compositionally biased sites removed using divvier (settings: -divvy -mincol 4 -divvygap) and then subsampled to 42 amino acids per marker for 143 Asgardarchaeota MAGs and archaeal representatives of all non-Asgard species in GTDB release r89 (1377 taxa, 5074 sites). Maximum-likelihood analysis was performed using IQ-TREE under the LG+C10+F+G+PMSF model. The tree is rooted on the Undinarchaeota. Numbers at branches indicate bootstrap statistical support (100 replicates). Asgardarchaeota classes are indicated with different color labels: Bright cyan - Sifarchaeia; dark yellow - Jordarchaeia; Light pink - Thorarchaeia; orange - Lokiarchaeia; Sky blue - Heimdallarchaeia; Light yellow - Odinarchaeia. Tree corresponds to the file “5.5_143x_r89_divvier_divvy_filtered_PMSFc10_100xBT.tree”, details are shown in Table S2.

**
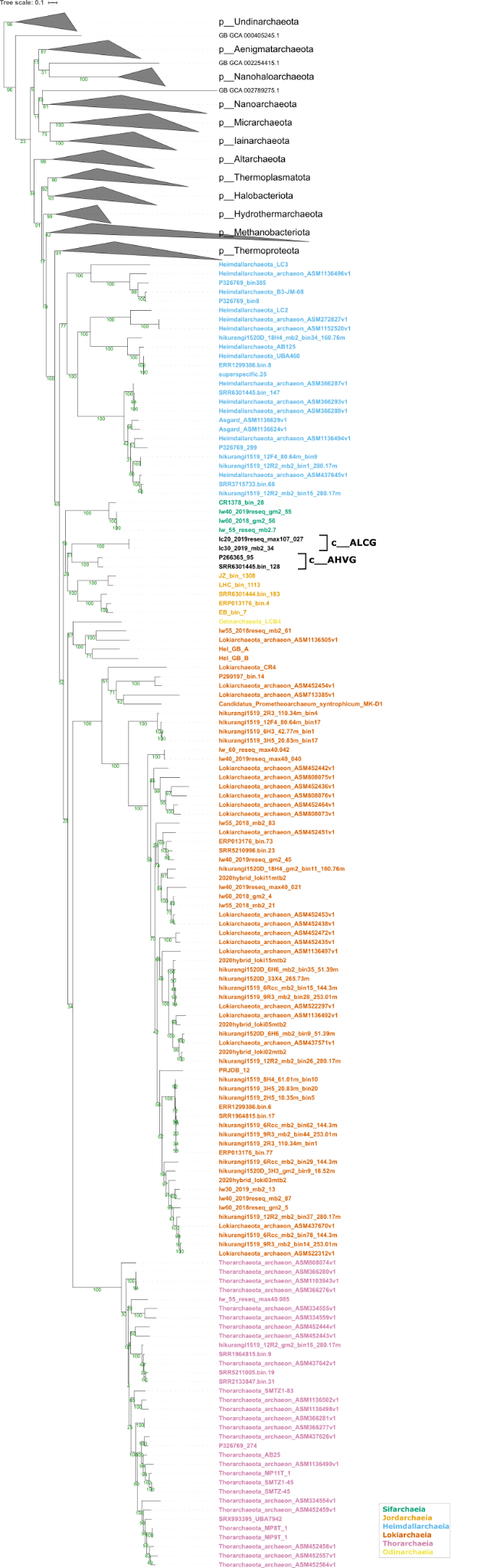
**

**Fig. S8 | Phylogenomic tree of 16 ribosomal protein markers against GTDB r89 taxa.** The alignment was based on a concatenated set of 16 protein markers for 143 Asgardarchaeota MAGs and archaeal representatives of all non-Asgard species in GTDB release r89 (1377 taxa, 2008 sites). Maximum-likelihood analysis was performed using IQ-TREE under the LG+C10+F+G+PMSF model. The tree is rooted on the Undinarchaeota. Numbers at branches indicate bootstrap statistical support (100 replicates). Asgardarchaeota classes are indicated with different color labels: Bright cyan - Sifarchaeia; dark yellow - Jordarchaeia; Light pink - Thorarchaeia; orange - Lokiarchaeia; Sky blue - Heimdallarchaeia; Light yellow - Odinarchaeia. Tree corresponds to the file “6.3_v3.0_r89_RP1_PMSFc10_100xBT.tree”, details are shown in Table S2.


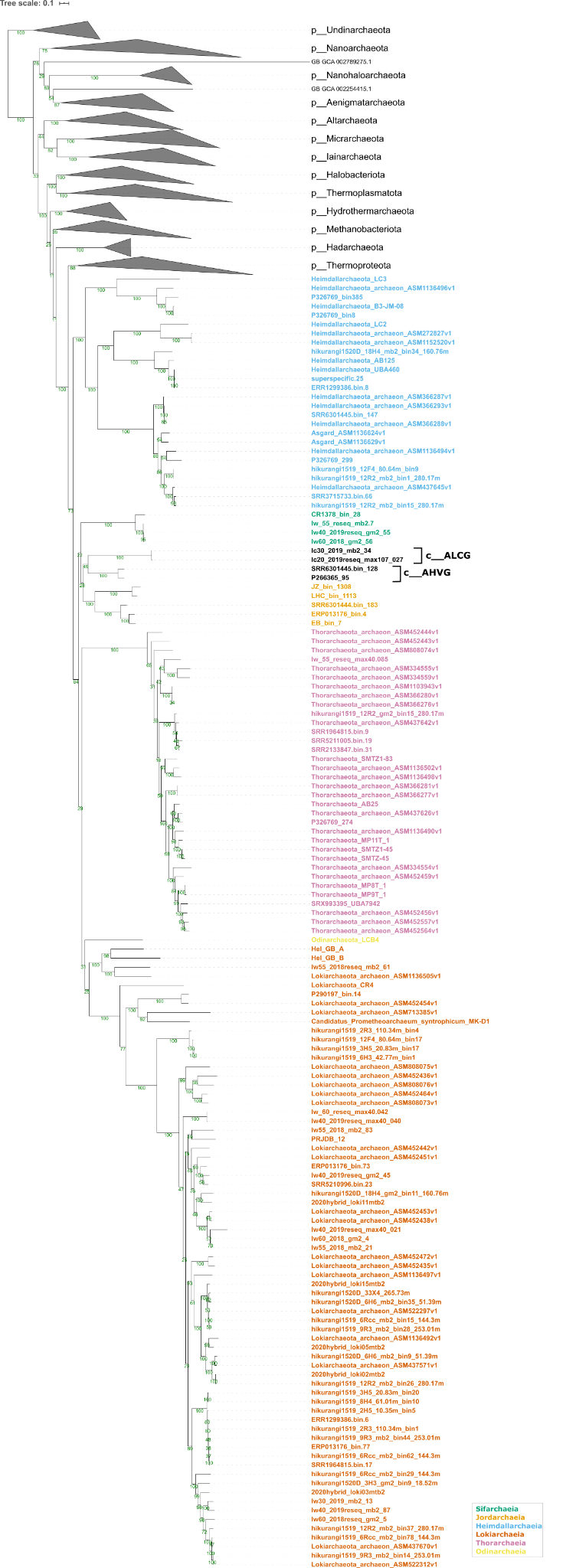


**Fig. S9 | Phylogenomic tree of 23 ribosomal protein markers against GTDB r89 taxa.** The alignment was based on a concatenated set of 23 protein markers for 143 Asgardarchaeota MAGs and archaeal representatives of all non-Asgard species in GTDB release r89 (1377 taxa, 3034 sites). Maximum-likelihood analysis was performed using IQ-TREE under the LG+C10+F+G+PMSF model. The tree is rooted on the Undinarchaeota. Numbers at branches indicate bootstrap statistical support (100 replicates). Asgardarchaeota classes are indicated with different color labels: Bright cyan - Sifarchaeia; dark yellow - Jordarchaeia; Light pink - Thorarchaeia; orange - Lokiarchaeia; Sky blue - Heimdallarchaeia; Light yellow - Odinarchaeia. Tree corresponds to the file “7.3_v3.0_r89_RP2_PMSFc10_100xBT.tree”, details are shown in Table S2.

**
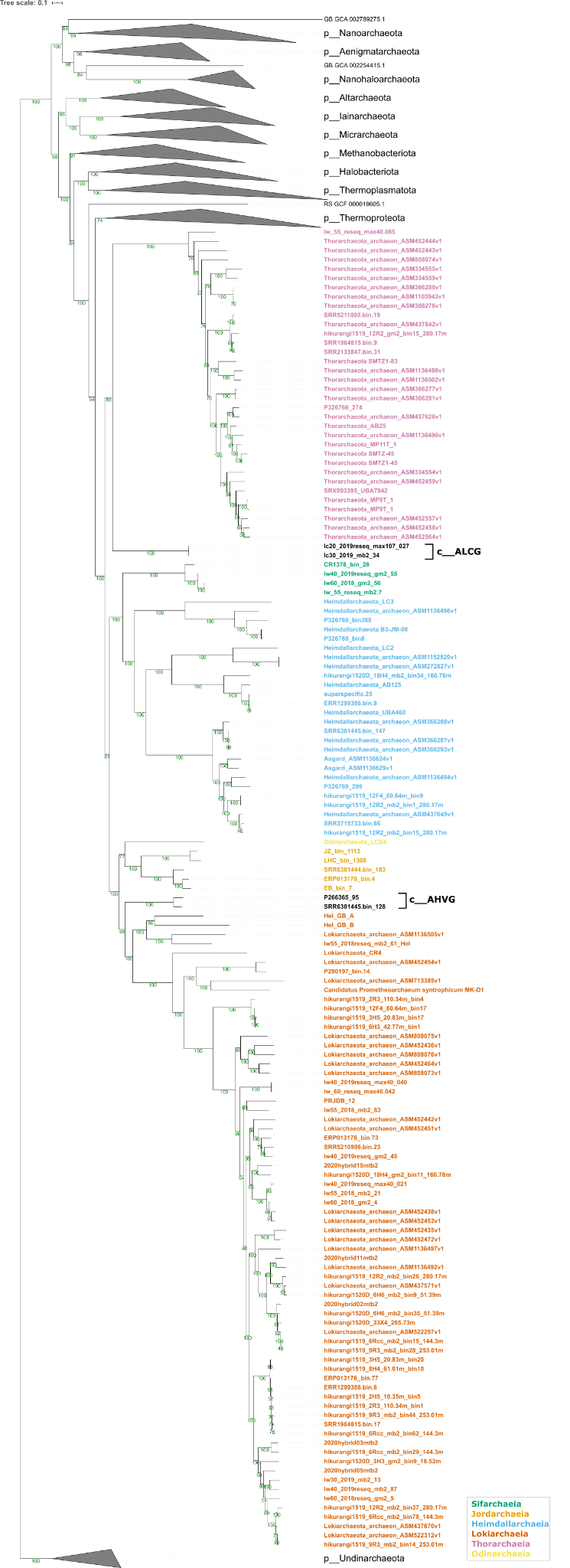
**

**Fig. S10 | Phylogenomic tree of 53 ribosomal protein markers against GTDB r89 taxa.** The alignment was based on a concatenated set of 53 protein markers for 143 Asgardarchaeota MAGs and archaeal representatives of all non-Asgard species in GTDB release r89 (1377 taxa, 13540 sites). Maximum-likelihood analysis was performed using IQ-TREE under the LG+C10+F+G+PMSF model. The tree is rooted on the Undinarchaeota. Numbers at branches indicate bootstrap statistical support (100 replicates). Asgardarchaeota classes are indicated with different color labels: Bright cyan - Sifarchaeia; dark yellow - Jordarchaeia; Light pink - Thorarchaeia; orange - Lokiarchaeia; Sky blue - Heimdallarchaeia; Light yellow - Odinarchaeia. Tree corresponds to the file “8.3_143x_r89_53xMarkers_PMSFc10_100xBT.tree”, details are shown in Table S2.

**
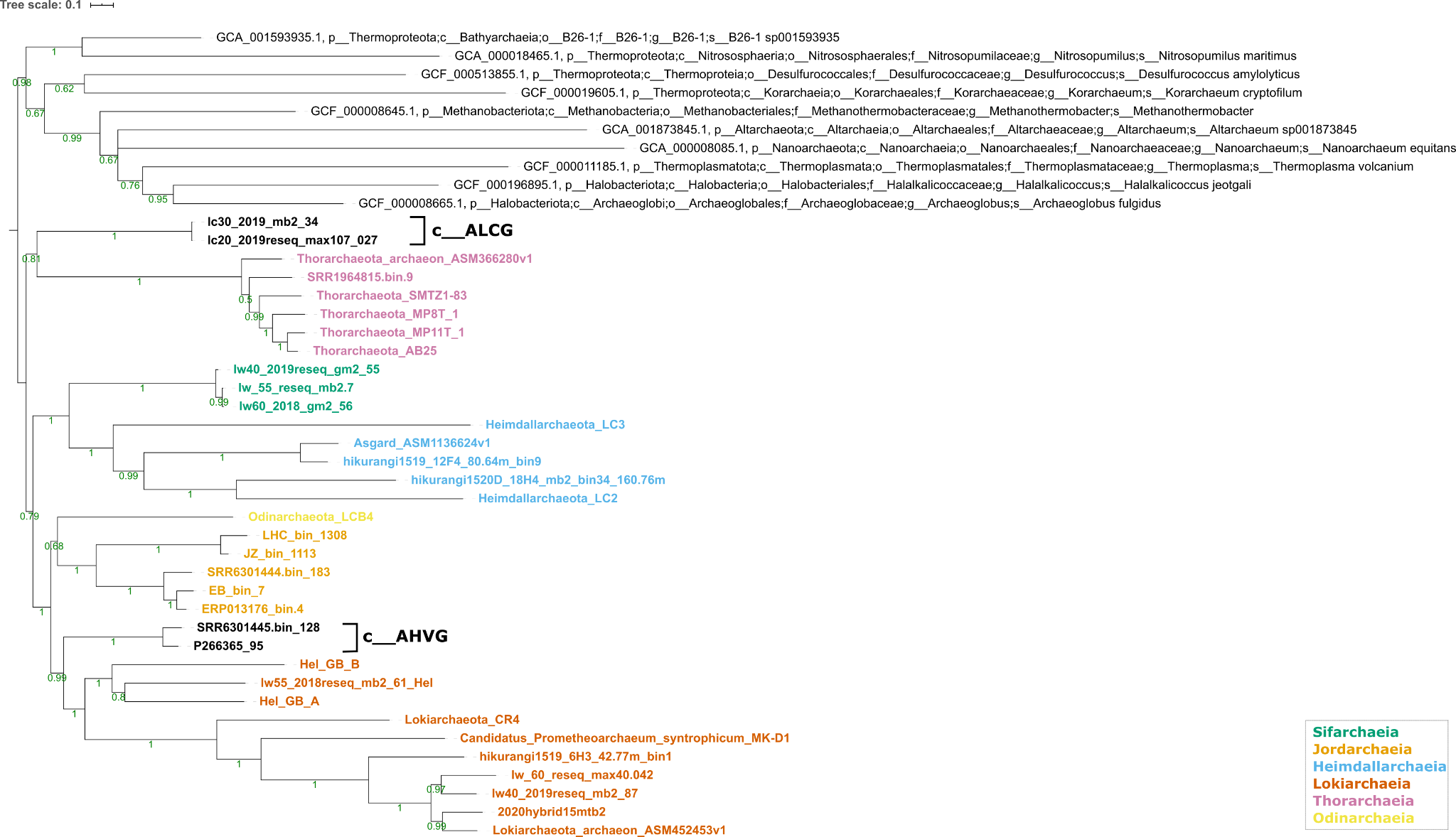
**

**Fig. S11 | Phylogenomic tree of 53 ribosomal protein markers against GTDB r89 taxa.** The alignment was based on a concatenated set of 53 protein markers for 143 Asgardarchaeota MAGs and archaeal representatives of all non-Asgard species in GTDB release r89 (1377 taxa, 13540 sites). Bayesian analysis was performed using PhyloBayes under the CAT+GTR model. The tree is rooted in between Asgardarchaeota and other Archaea. Numbers at branches indicate bayesian support values. Asgardarchaeota classes are indicated with different color labels: Bright cyan - Sifarchaeia; dark yellow - Jordarchaeia; Light pink - Thorarchaeia; orange - Lokiarchaeia; Sky blue - Heimdallarchaeia; Light yellow - Odinarchaeia. Tree corresponds to the file “8.9_genomes45x_53xMarkers_bayesian.tree”, details are shown in Table S2.

**
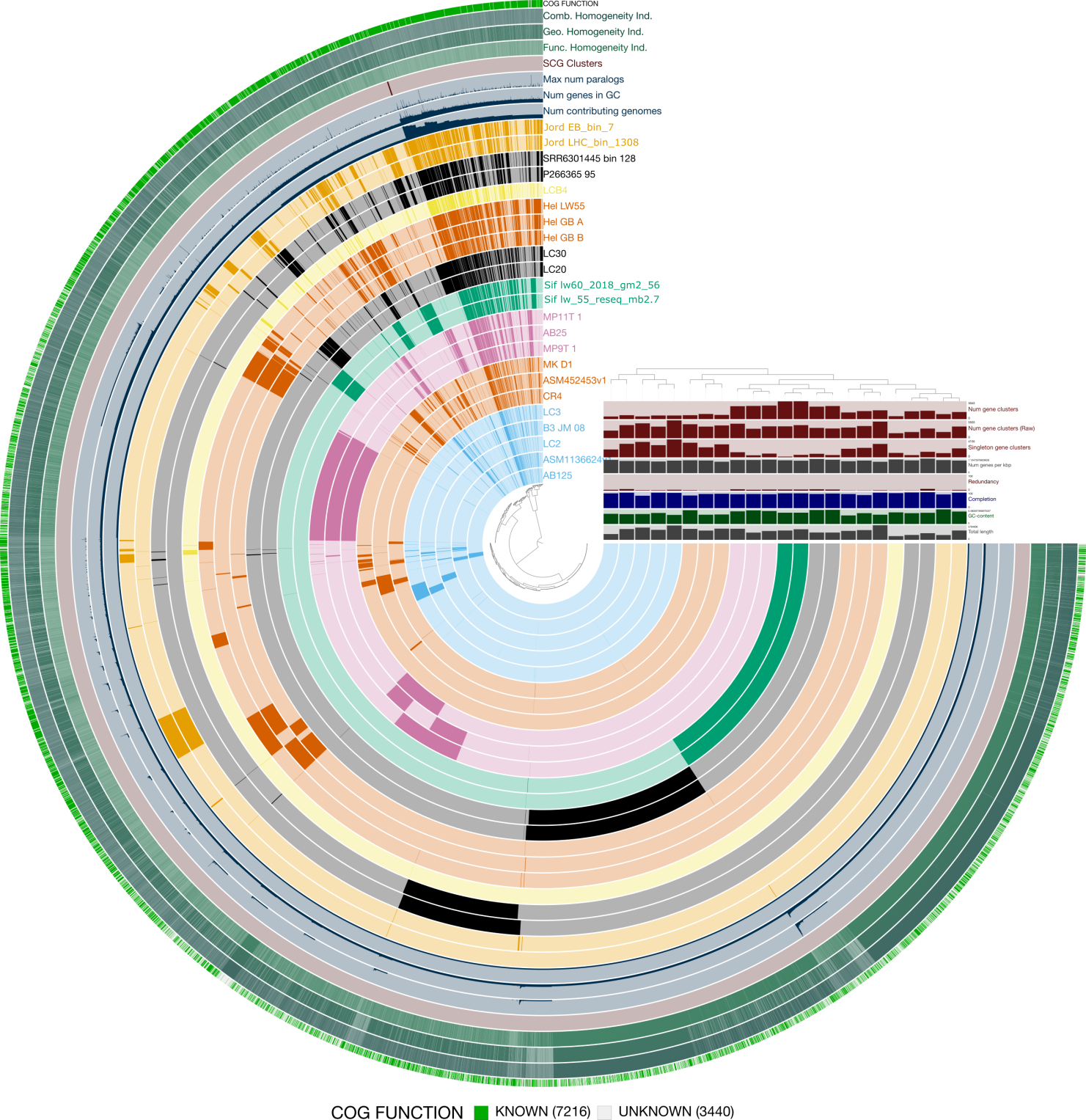
**

**Fig. S12 | Pangenomic analysis of protein clusters within Asgardarchaeota genomes.** The analysis was performed with Anvi’o (option ‘--min-occurrence 2’), see methods. The inner tree is based on protein clusters of COG functions. Asgardarchaeota classes are indicated with different color layers: Bright cyan - Sifarchaeia; dark yellow - Jordarchaeia; Light pink - Thorarchaeia; orange - Lokiarchaeia; Sky blue - Heimdallarchaeia; Light yellow - Odinarchaeia; Black - Asgard hydrothermal vent group (AHVG) and Asgard Lake Cootharaba group (ALCG).


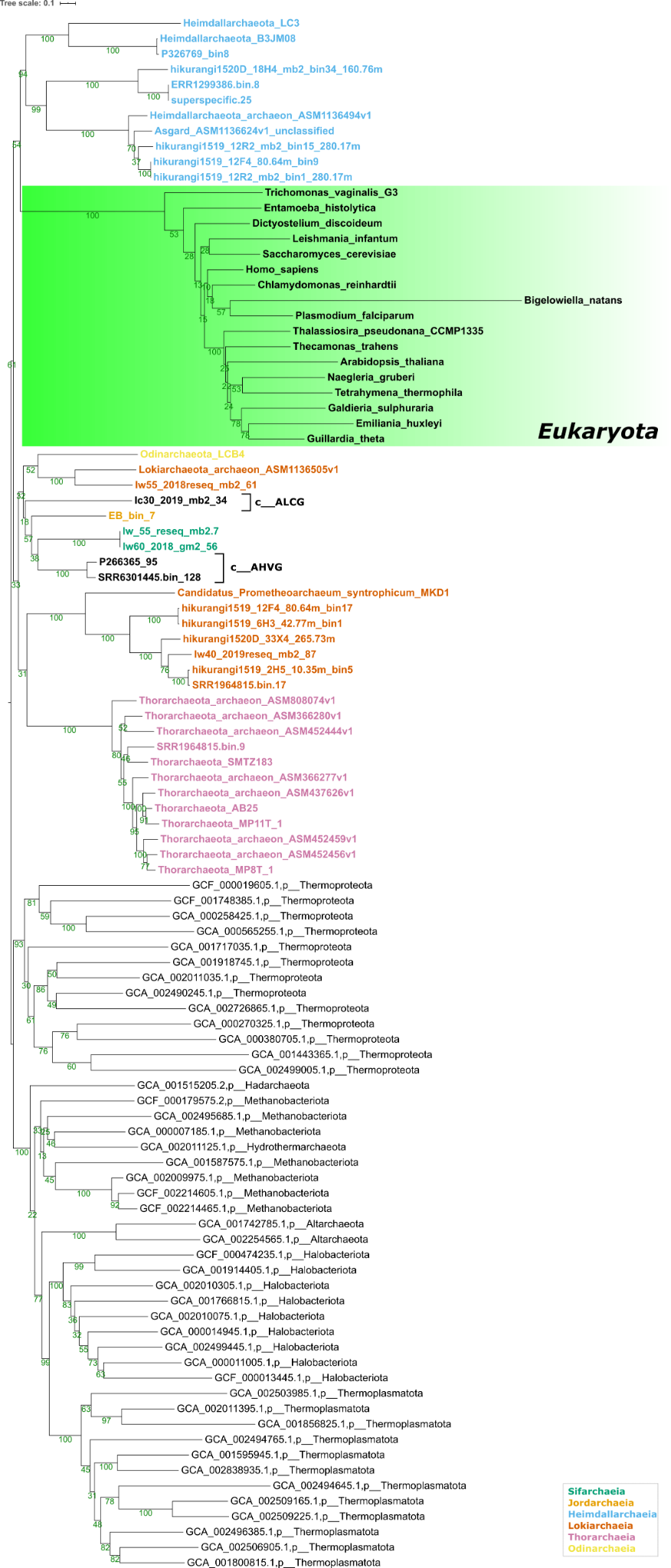


**Fig. S13 | Phylogenomic tree of 15 COG ribosomal protein markers.** The alignment (101 taxa, 1788 columns) was based on a concatenated set of 15 ribosomal protein markers based on Clusters of Orthologous Groups of proteins (COGs). Maximum-likelihood analysis was performed using IQ-TREE under the LG+C60+F+G+PMSF model. The tree is rooted between Asgardarchaeota and the rest of archaea phyla. Numbers at branches indicate bootstrap statistical support (100 replicates). Asgardarchaeota classes are indicated with different color labels: Bright cyan - Sifarchaeia; dark yellow - Jordarchaeia; Light pink - Thorarchaeia; orange - Lokiarchaeia; Sky blue - Heimdallarchaeia; Light yellow - Odinarchaeia. Eukaryotic genomes are highlighted with a green square. Tree corresponds to the file “1_2Domain_PMSFc10.tree”.

**
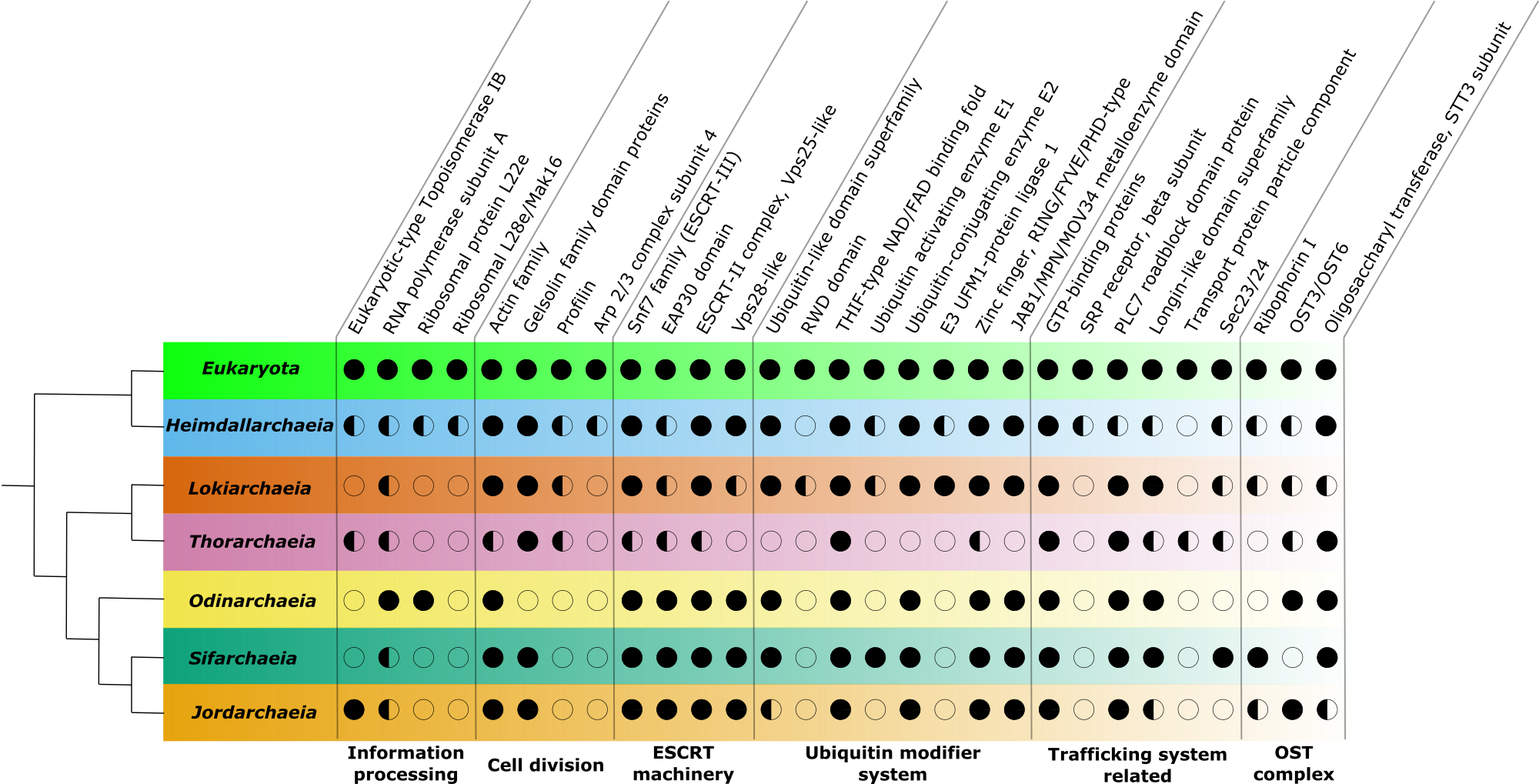
**

**Fig. S14 | Distribution of eukaryotic signature proteins in Asgardarchaeota.** The schematic representation of Asgardarchaeota classes and Eukaryota is based on the archaeal-eukaryotic tree (**Fig. S13**). This figure summarises the presence of eukaryotic signature proteins (ESPs) based on MAGs with completeness > 90%. Black circles indicate that all high-completeness MAGs in a given class encode the ESP homologs; black semicircles represent the detection of certain ESP homologs from at least one MAG; and white circles indicate no detection of a certain ESP.

**(a)**

**
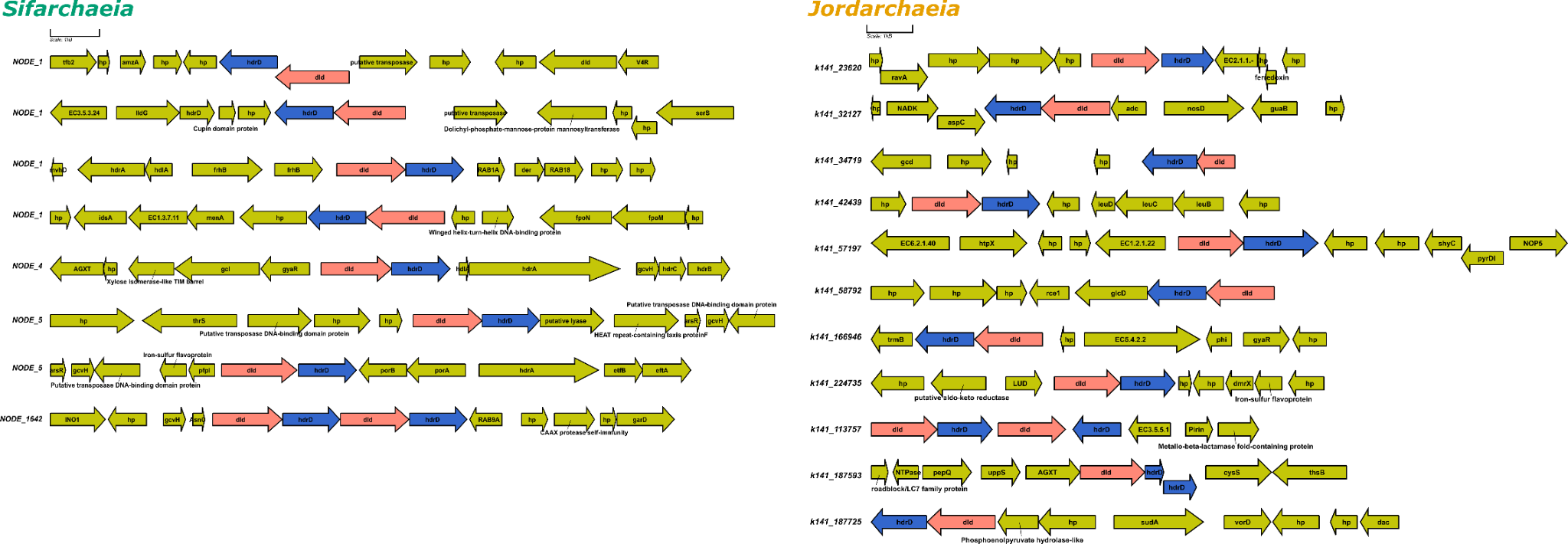
**

**(b)
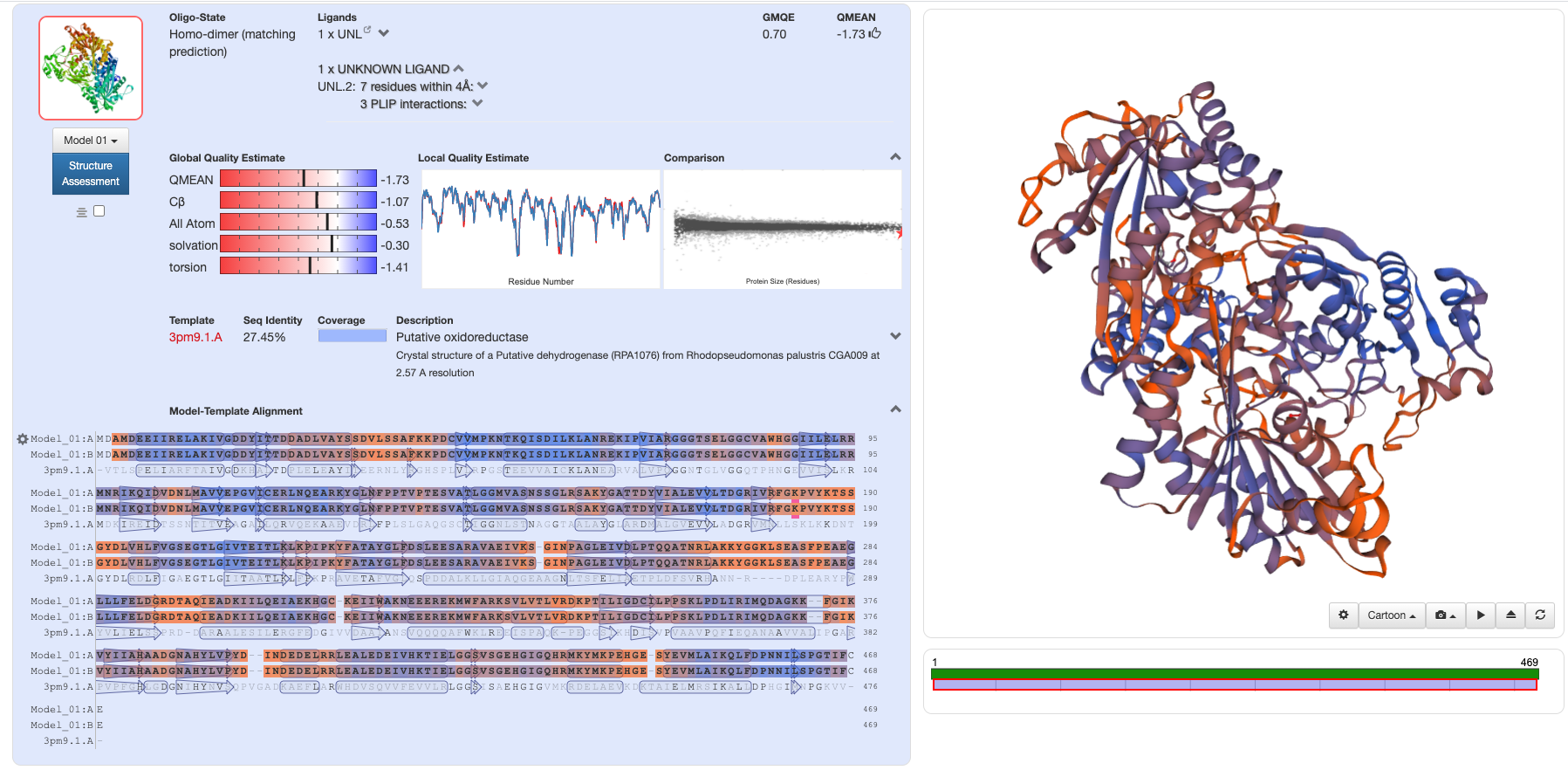
**

**(c)**

**
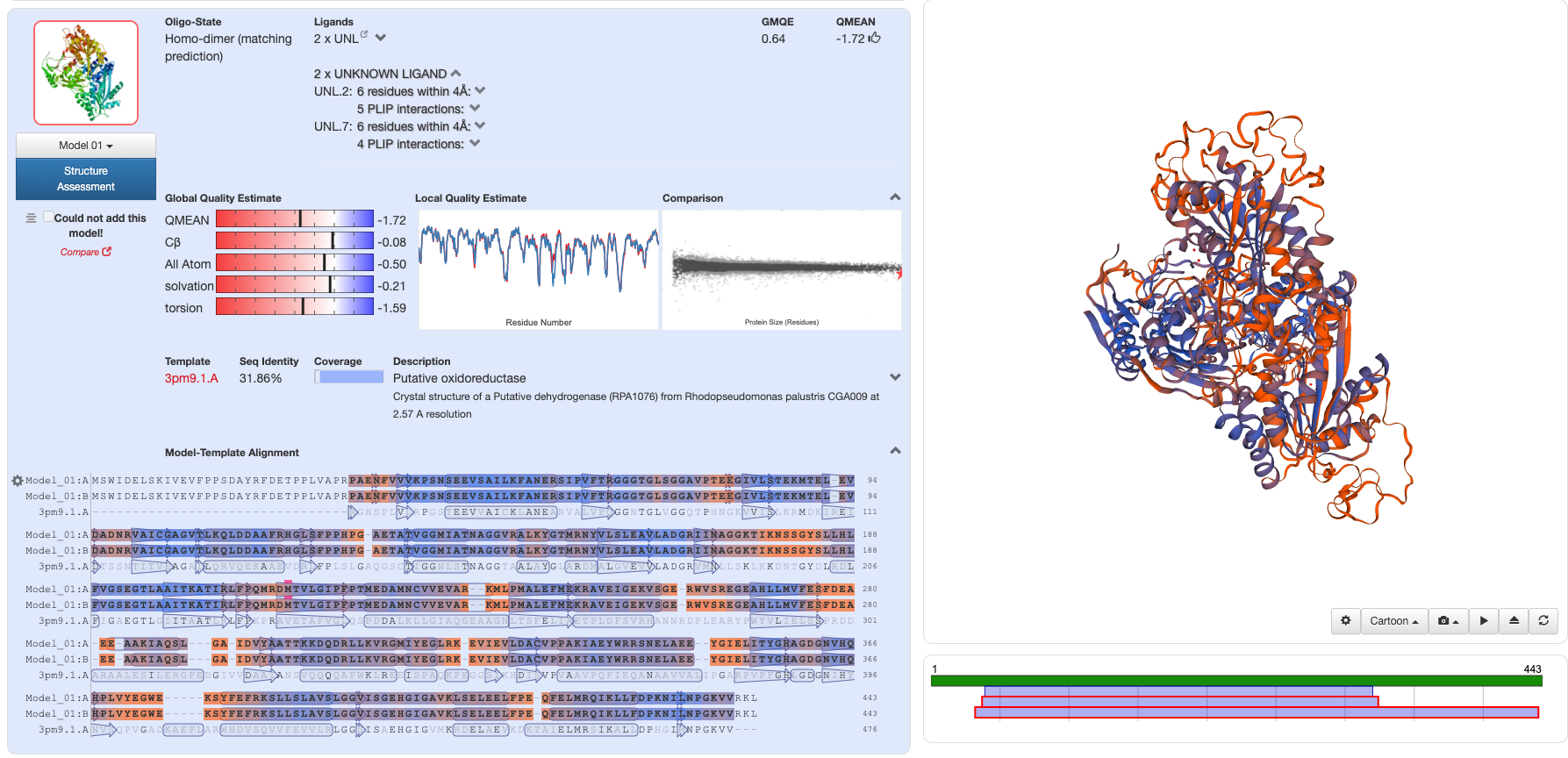
**

**(d)**

**
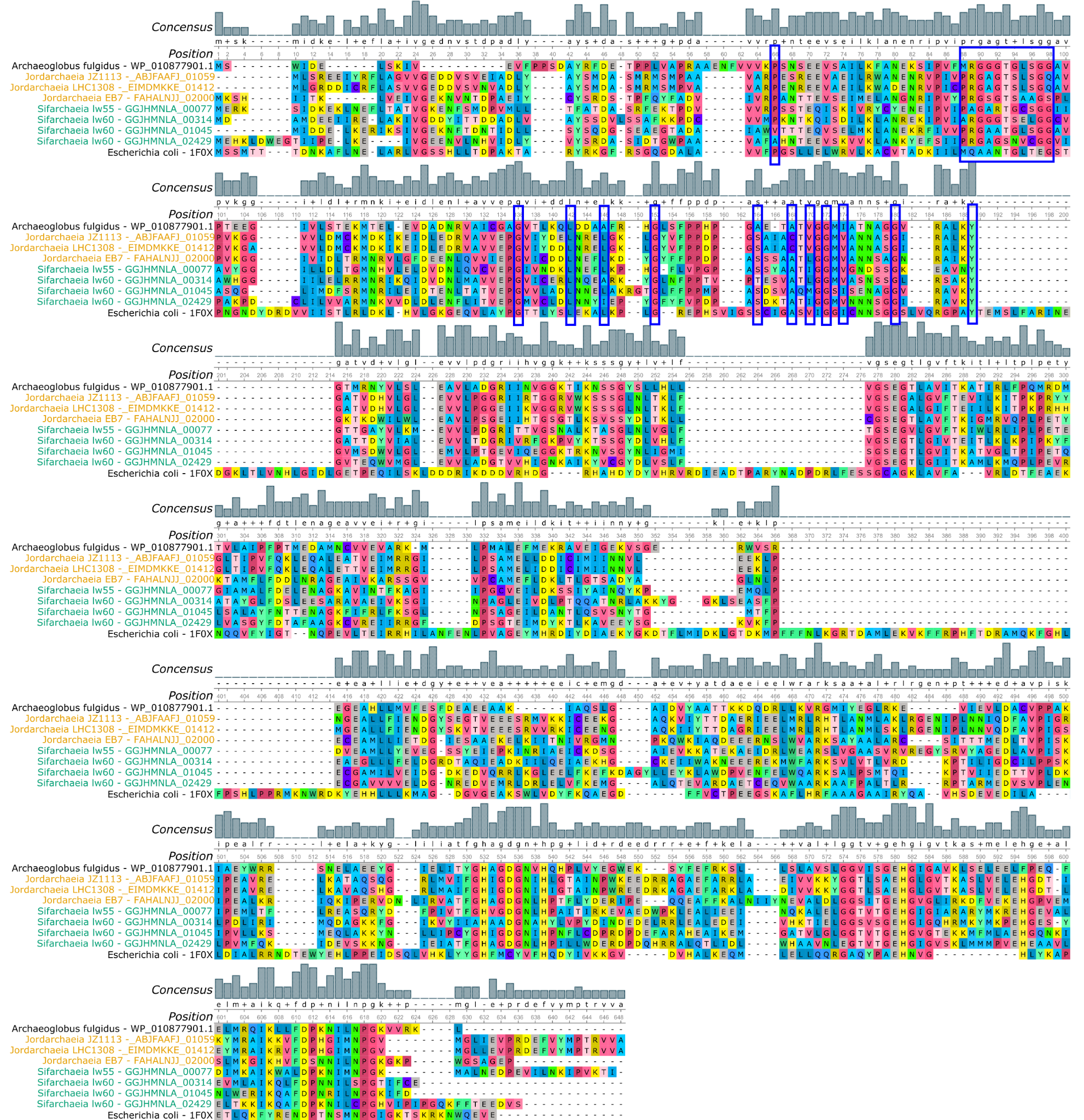
**

**(e)**

**
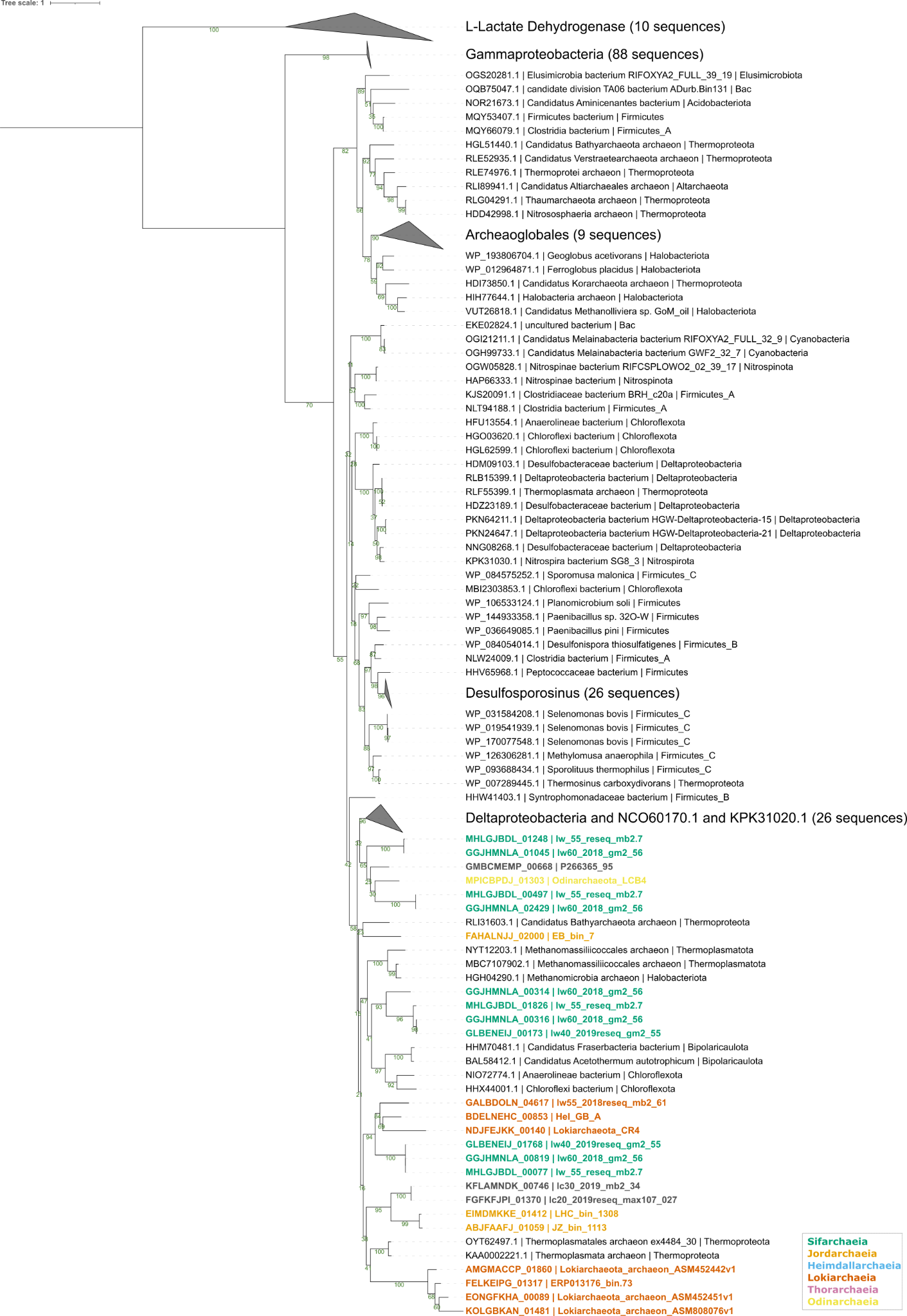
**

**Fig. S15 | D-lactate dehydrogenase (Dld). a**. Gene neighbourhood of D-lactate dehydrogenase-heterodisulfide reductase subunit D complex (Dld-HdrD). Up to five neighbouring genes upstream and downstream of each Dld-HdrD gene cluster from the Sifarchaeia type genome (lw60_2018_gm2_56; 9 copies) and the Jordarchaeia type genome (EB_bin_7 ; 13 copies) are shown. Dld genes are highlighted in light red, and HdrD genes are highlighted in dark blue. **b,c**. 3D structures of Sifarchaeia Dld sequence GGJHMNLA_008314 (b) and Archaeoglobus fulgidus Dld sequence AAB90435.1 (c). The protein structure modelling was predicted by SWISS-MODEL (<https://swissmodel.expasy.org/>). **d**. Multiple sequence alignment of D-lactate dehydrogenase sequences. Previously proposed FAD-binding sites (Dym, et al., 2000) are highlighted in blue boxes. **e.** Phylogenetic tree of Dld. Tree was inferred with IQ-TREE (LG+C10+F+G+PMSF model) from a 511-position alignment (after removing columns of less than 40% consensus) with bootstrap support values (green numbers at internal nodes ) based on 100 trees under the same model. Tree was rooted on the group containing ten L-Lactate dehydrogenase sequences. Asgardarchaeota sequences are represented with different color labels: Bright cyan - Sifarchaeia; dark yellow - Jordarchaeia; Light pink - Thorarchaeia; orange - Lokiarchaeia.

**
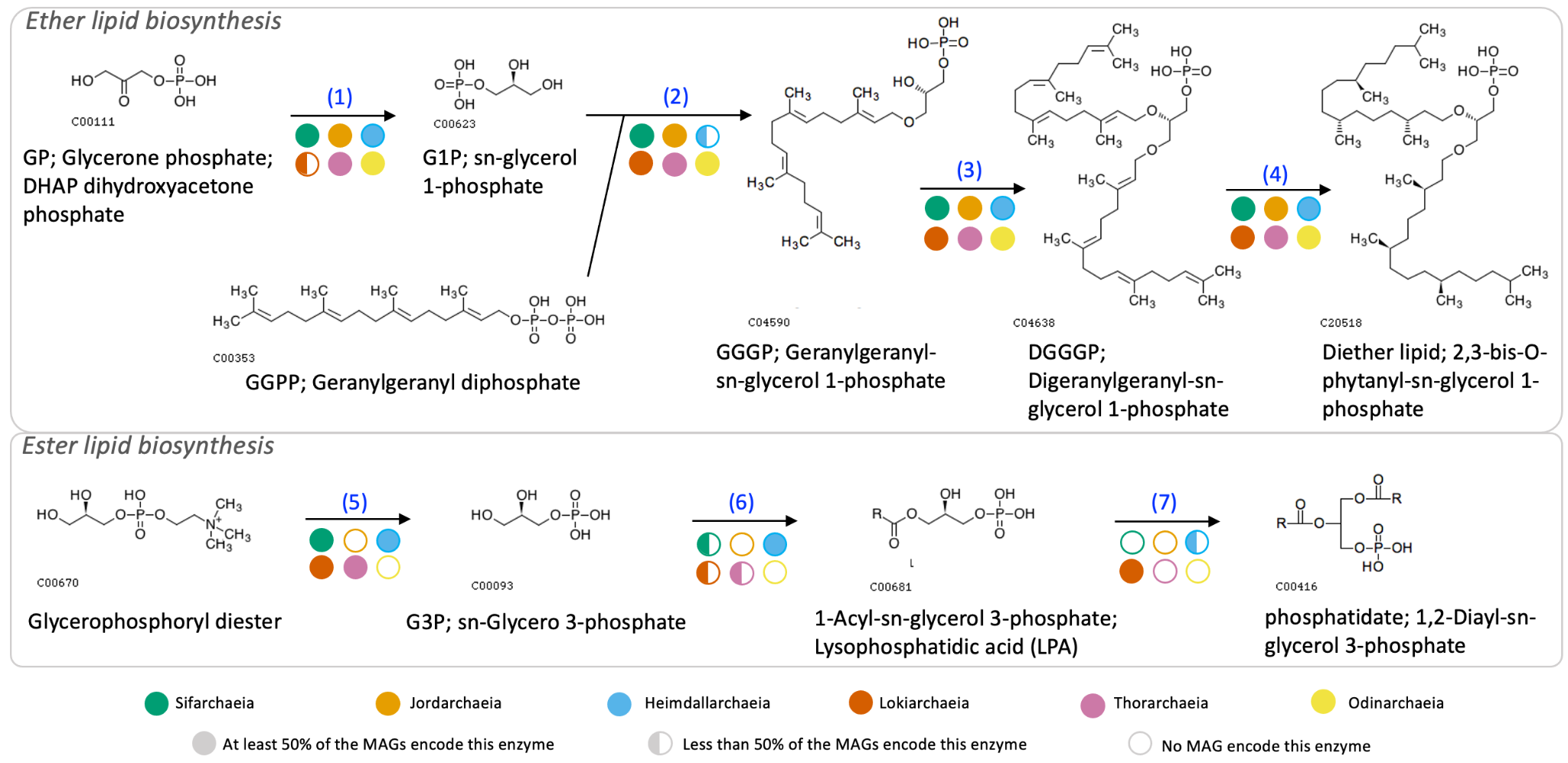
**

**Fig. S16 | Biosynthetic pathway of membrane lipids in Asgardarchaeota.** Archaeal membranes are composed of branched hydrocarbon chains which are attached to glycerol by ester linkages, whereas bacteria and eukaryotes synthesize membranes with unbranched fatty acid chains with ester-bond glycerol. Genes involved in both types of lipid biosynthesis have been reported previously for several Asgardarchaeota groups (Coleman et al., 2019) and were identified in Sif-and Jordarchaeia MAGs. Enzymes catalyzing biosynthesis reactions are shown as blue numbers: 1 - araM, egsA; glycerol-1-phosphate dehydrogenase [NAD(P)+] [EC:1.1.1.261] (K00096). 2 - DGGGP synthase; phosphoglycerol geranylgeranyltransferase [EC:2.5.1.41] (K17104). 3 - DGGGPL; GGR; E2.5.1.42; geranylgeranylglycerol-phosphate geranylgeranyltransferase [EC:2.5.1.42] (K17105). 4 - DGGGPL; GGR; digeranylgeranylglycerophospholipid reductase [EC:1.3.1.101 1.3.7.11] (K17830). 5 – glpQ/ugpQ; glycerophosphoryl diester phosphodiesterase [EC:3.1.4.46] (K01126). 6 - plsY; acyl phosphate:glycerol-3-phosphate acyltransferase [EC:2.3.1.275] (K08591). 7 - plsC; 1-acyl-sn-glycerol-3-phosphate acyltransferase [EC:2.3.1.51] (K00655). Enzymes encoded by MAGs in each Asgardarchaeota class are shown as pie charts with different colors. Detailed metabolic information for the MAGs is available in **Table S4**.

**
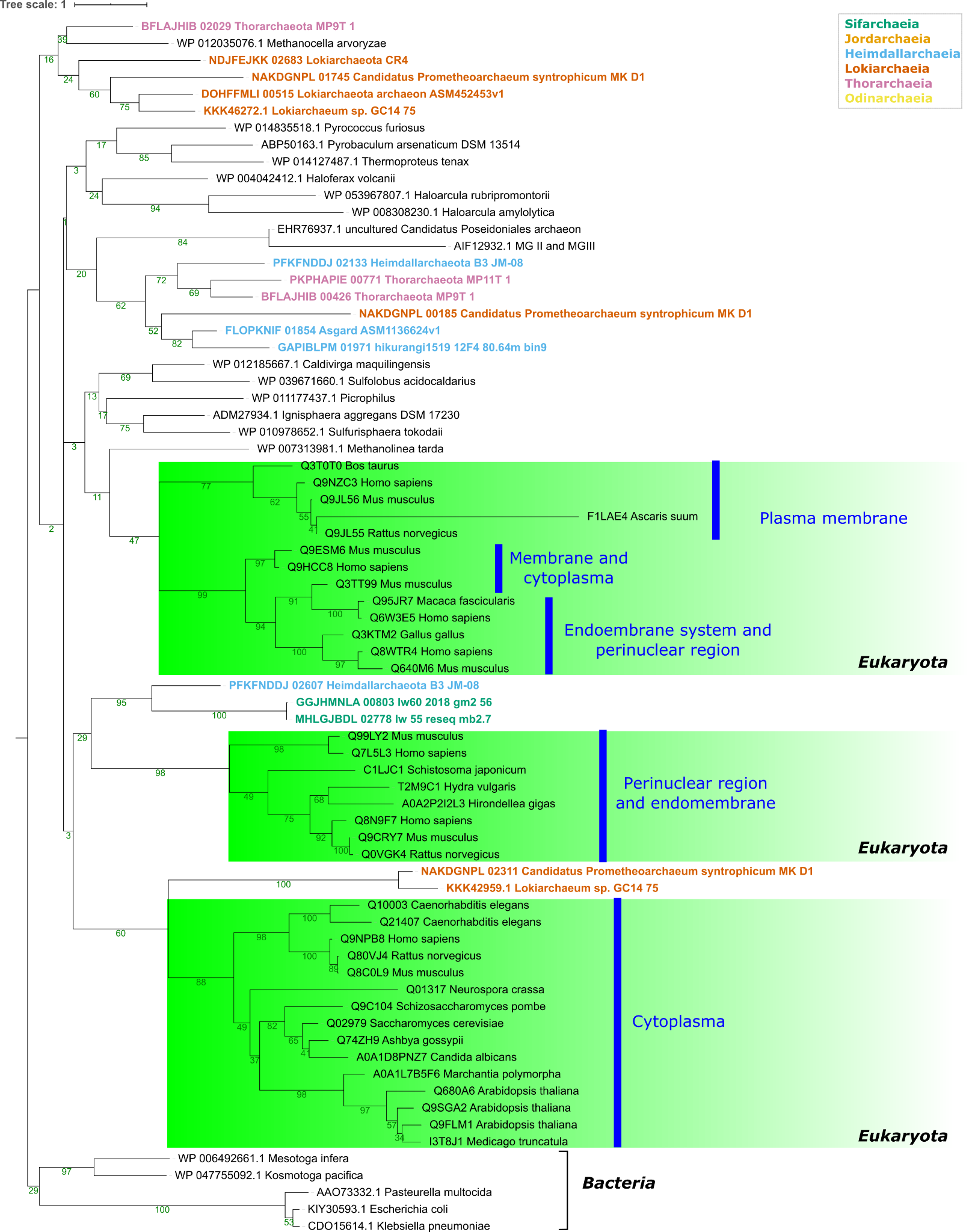
**

**Fig. S17| Phylogenetic tree of key ester-type lipid gene, glycerophosphoryl diester phosphodiesterase (glpQ/ugpQ).** Tree was inferred with IQ-TREE (LG+C10+F+G+PMSF model) from a 583-position alignment with bootstrap support values (green numbers under branches) based on 100 trees under the same model. Tree was rooted on the Bacteria. Eukaryotic genes are highlighted with green squares, with Uniprot “subcellular location” added in blue font. Asgardarchaeota sequences are represented with different color labels: Bright cyan - Sifarchaeia; dark yellow - Jordarchaeia; Light pink - Thorarchaeia; orange - Lokiarchaeia.

**(a)**
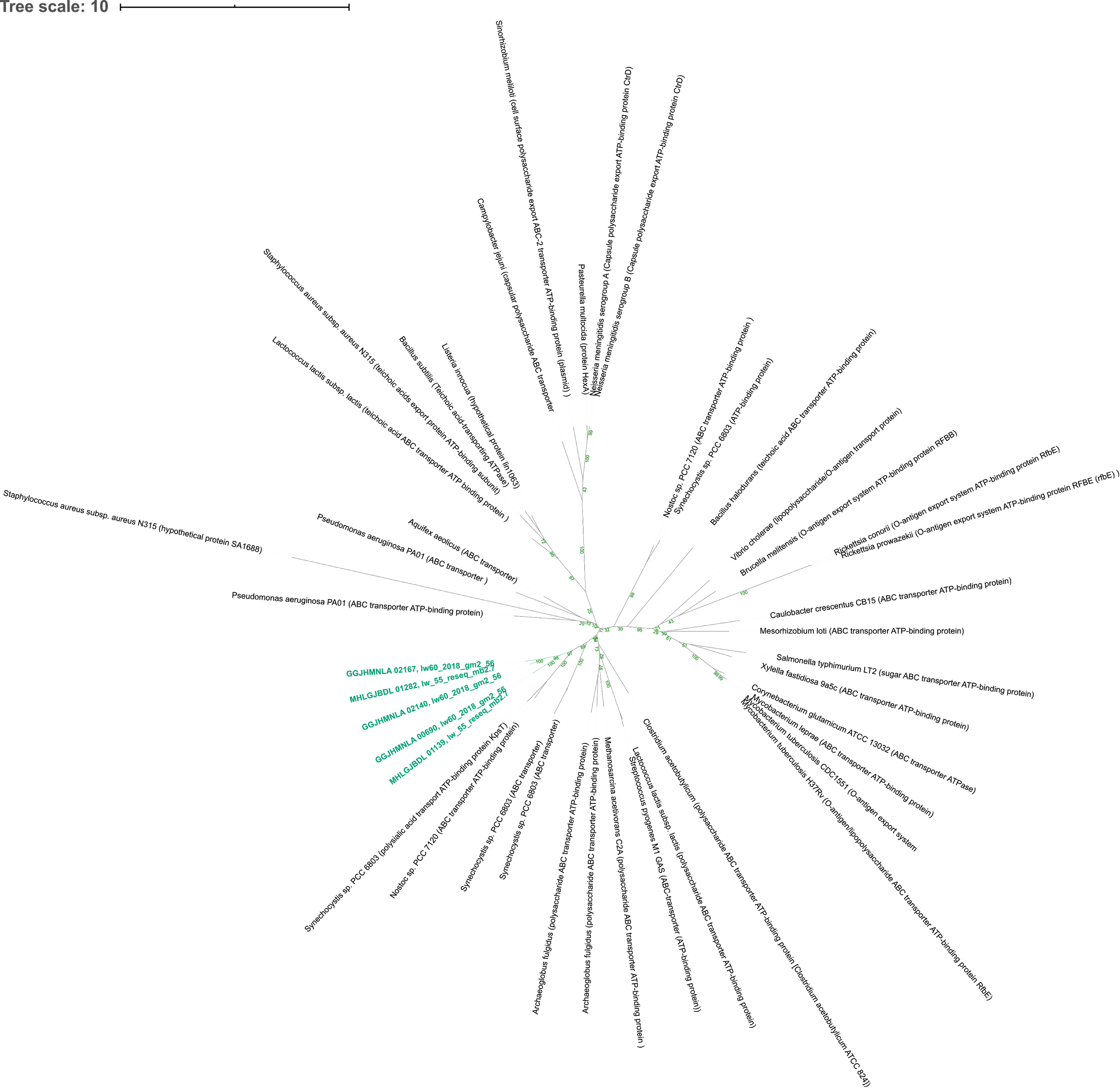


**(b)**

**
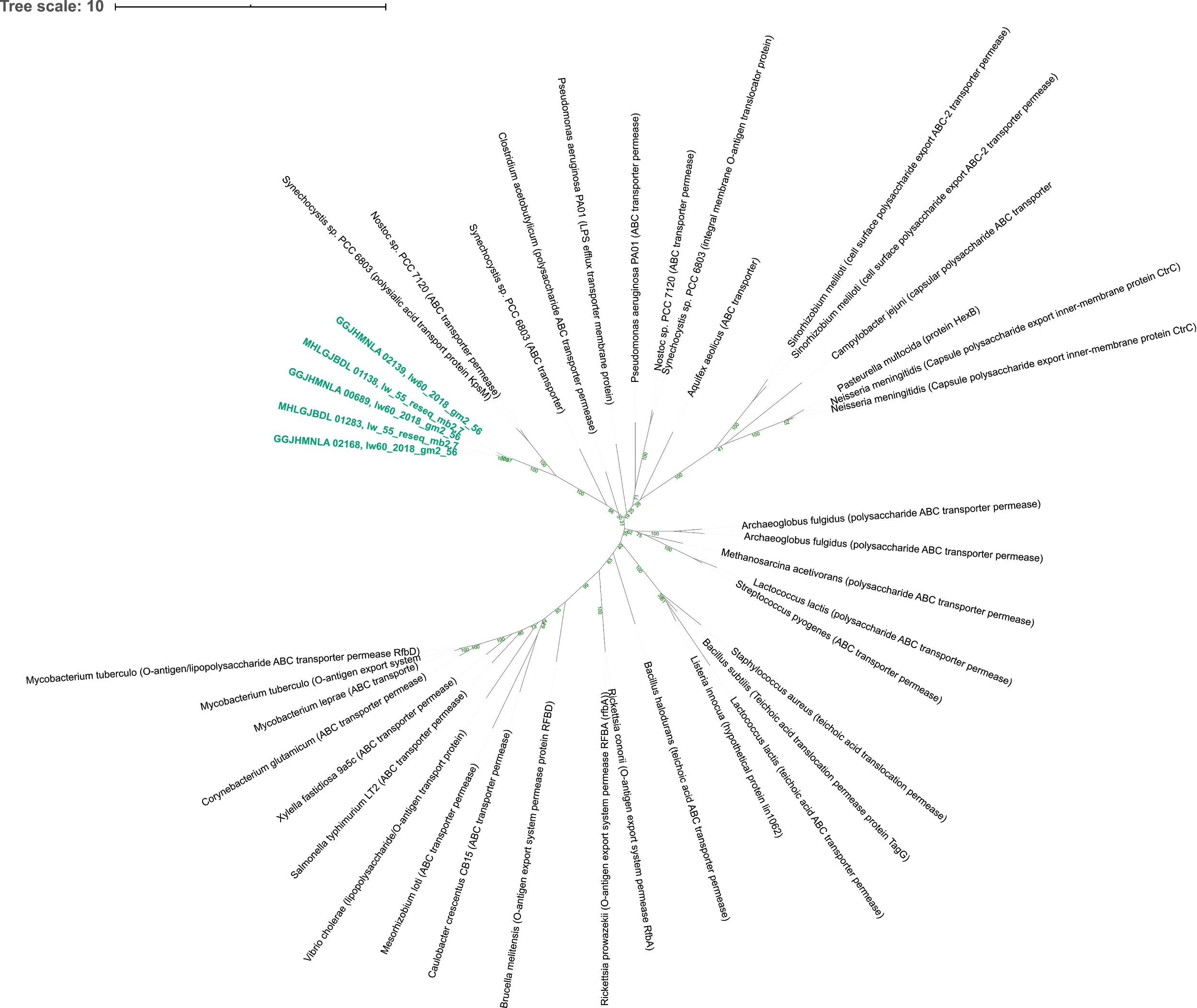
**

**Fig. S18 | Phylogenetic trees of TagGH ABC-type transporters.** **a.** Phylogenetic tree of TagH (COG1134), encoding the ABC-type polysaccharide/polyol phosphate transport system ATPase component, inferred from an 693-position alignment**. b.** Phylogenetic tree of TagG (COG1682), encoding the ABC-type polysaccharide/polyol phosphate export permease, inferred from a 360-position alignment. Both unrooted trees were reconstructed using IQ-TREE with the LG+C10+F+G+PMSF model. Bootstrap supported values were calculated based on 100 bootstrap trees and indicated by the numbers next to each branch. Sifarchaeia sequences were highlighted in bright cyan. NCBI protein definitions for reference sequences are indicated inside brackets following the organism name.

**(a)… .
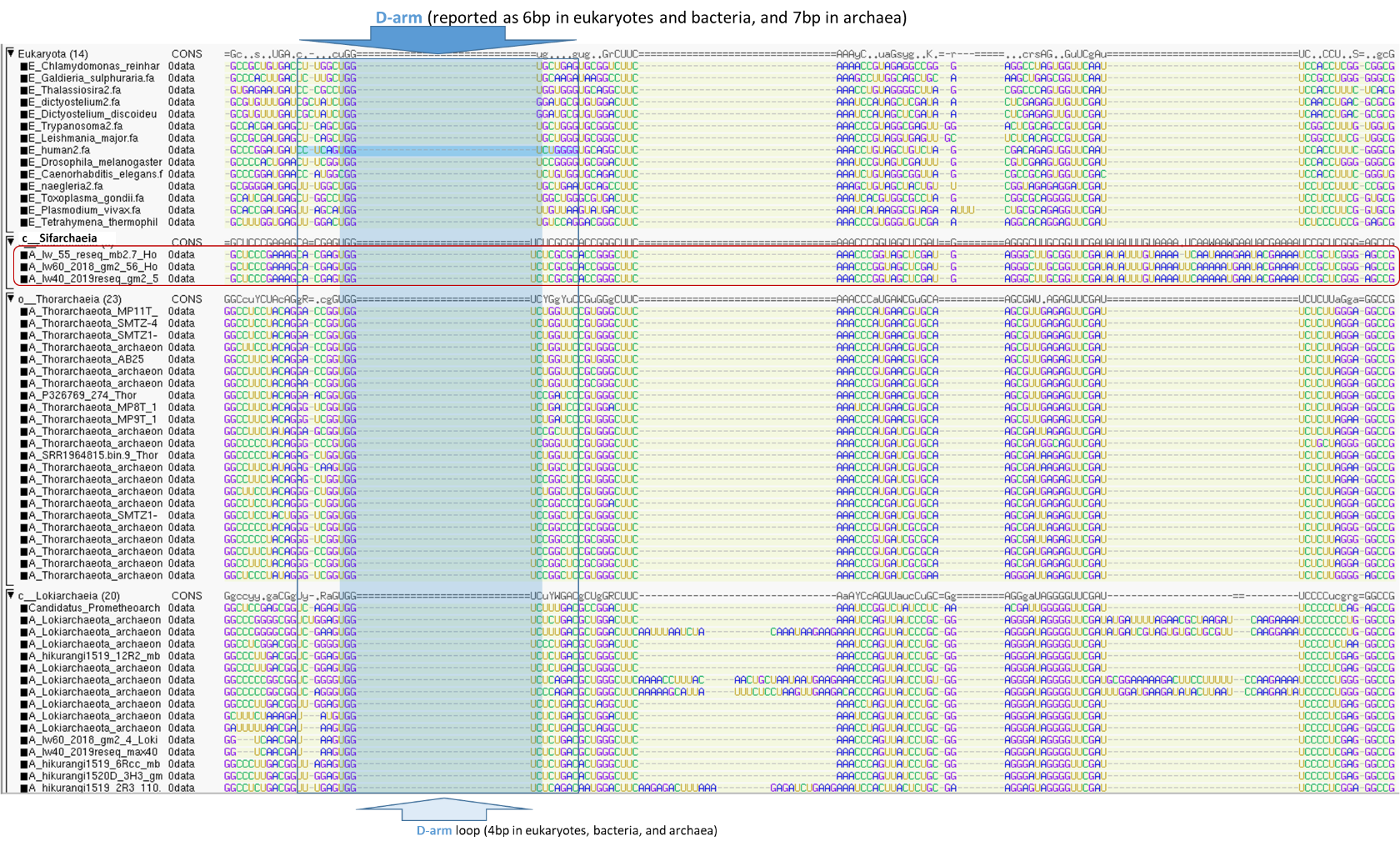

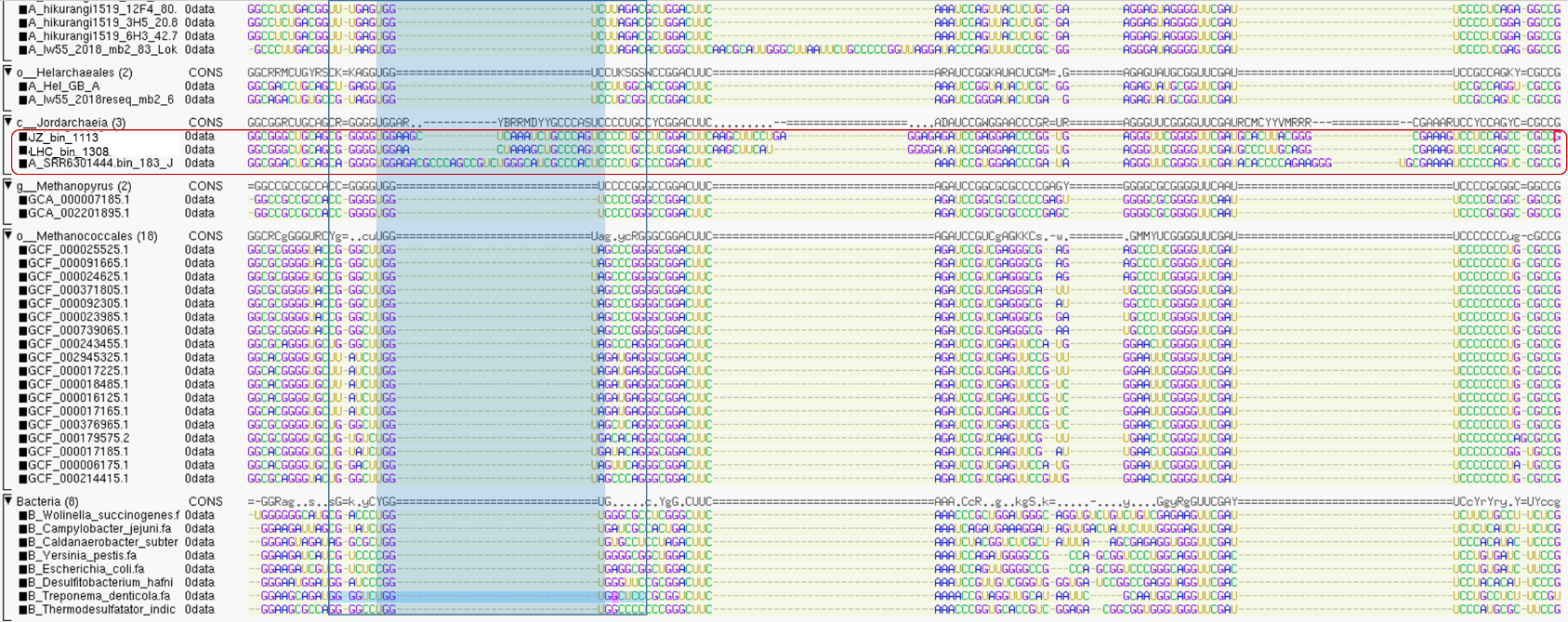
**

**(b)**

**
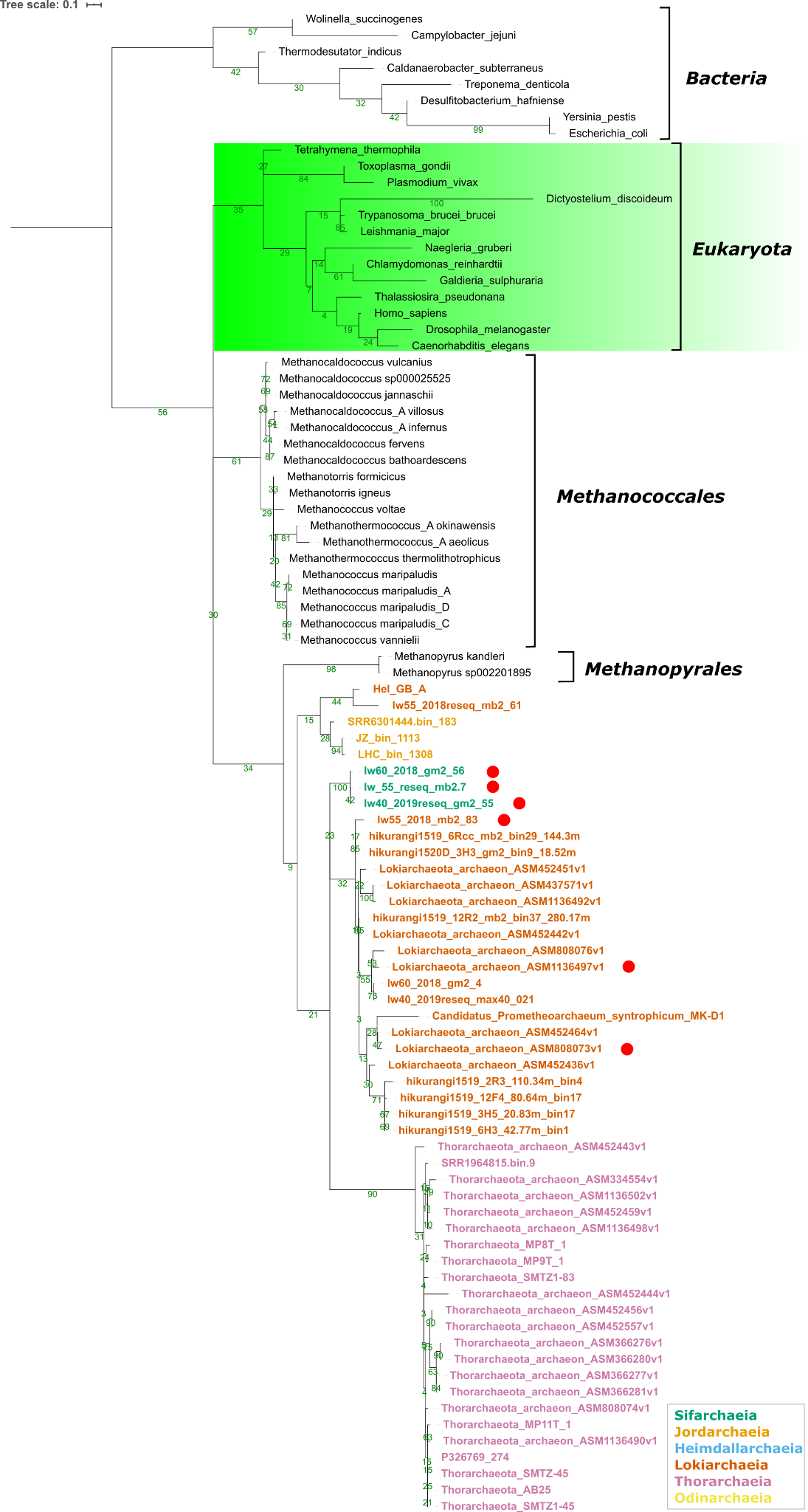
**

**Fig. S19 | Sequence alignment and phylogenetic tree of selenocysteine tRNA (tRNA-Sec). a.** Sequence alignment of tRNA-Sec. **Sif**- and Jordarchaeia sequences are highlighted with red rectangles. **b.** Phylogenetic tree of selenocysteine tRNA (tRNA-Sec) sequences. Tree was inferred with IQ-TREE (TVM+F+I+G4) from a trimmed alignment (consensus 40 to 100%) of 196 columns, and rooted at the bacterial node. Bootstrap support values are based on 100 IQ-TREE trees under the same model. The ‘eukaryotic-type’ tRNAsec (with 6bp D-stems) are indicated with red dots. Note that while bootstrap supports are low throughout the tree, most Asgardarchaeota lineages were recovered as monophyletic. These consistencies between the selenocysteine t-RNA tree and the protein maker genome trees are, despite the short sequence length, the reported frequent horizontal gene transfers and extensive paralogy through gene duplication in tRNAs, not overly surprising. In particular, since a previous study found the tRNA-Sec trees had the best correlation (r = 0.91), among all tRNA trees, with universal rRNA trees derived from the same genomes (Widmann et al., 2010).


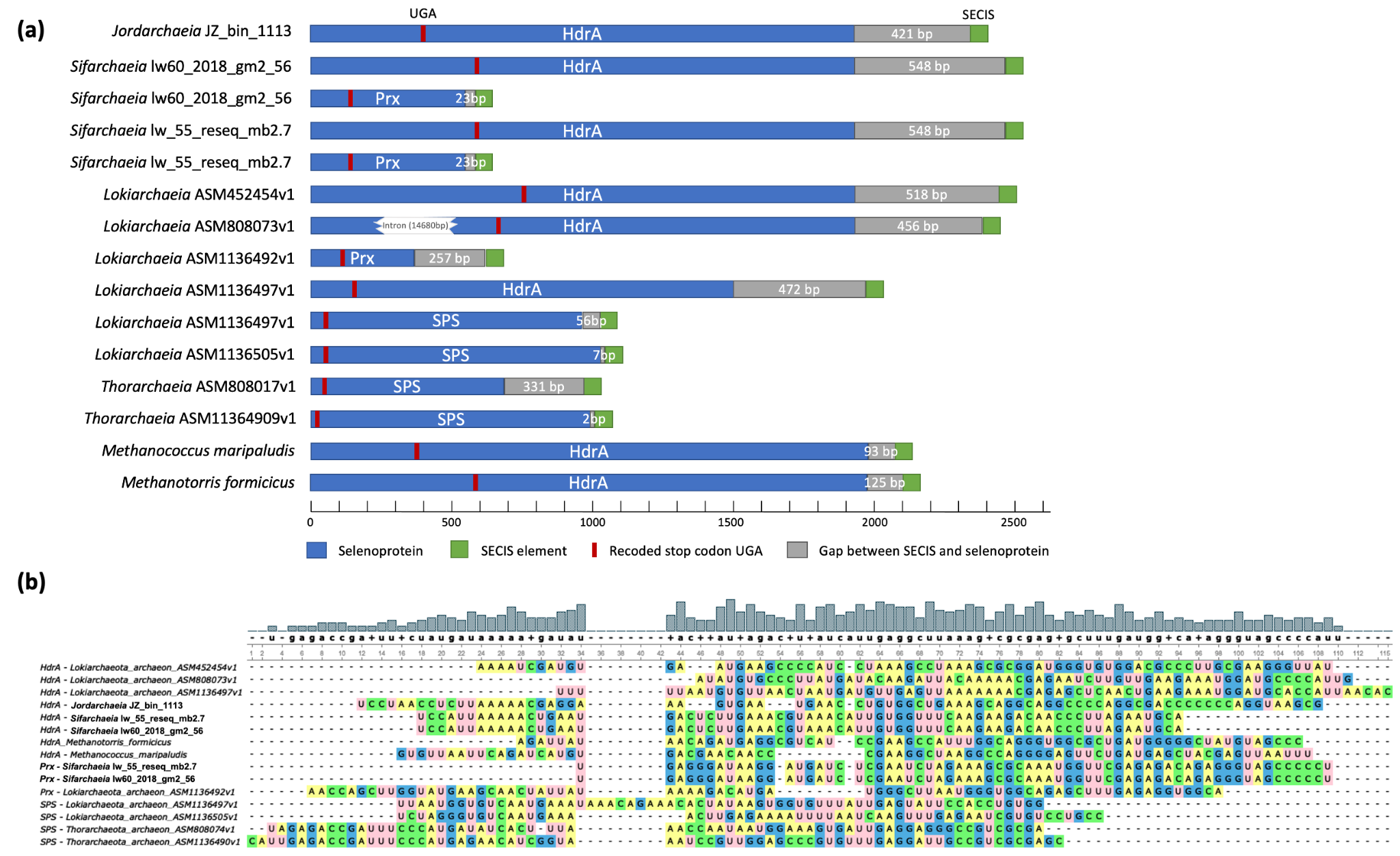


**Fig. S20 | SECIS elements detected in Asgardarchaeota. a.** Locations of SECIS elements and Selenoproteins in Asgardarchaeota MAGs. Selenoproteins and their corresponding SECIS elements were detected by Seblastian (Mariotti et al., 2013) and are represented as blue boxes and green boxes, respectively. We selected *Methanococcus maripaludis* and *Methanotorris formicicus* as the representatives of the class Methanococcales. Although two representative genomes of class Methanopyrales encode the Sec-encoding system, Seblastian failed to identify their selenoproteins. The lengths of all SECIS elements shown in this figure are between 59 bp and 75 bp. Detailed information can be found in **Table S13**. **b.** Sequence alignment of the SECIS elements shown in (a). The SECIS sequences were aligned using MAFFT v7.455 (Katoh and Standley, 2013) and the figure was created by UGene (Okonechnikov et al., 2012).

**(a)**

**
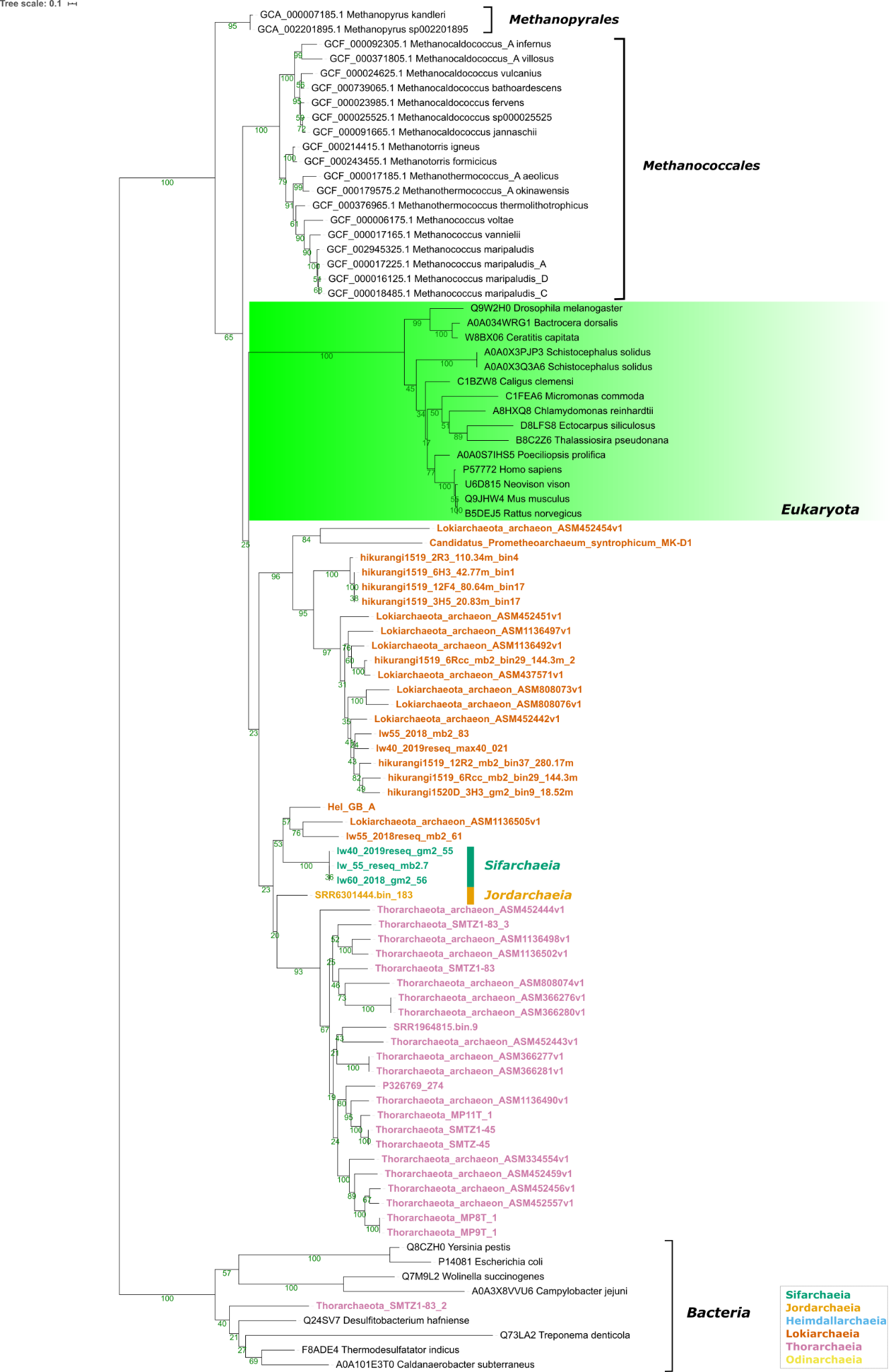
**

**(b)**

**
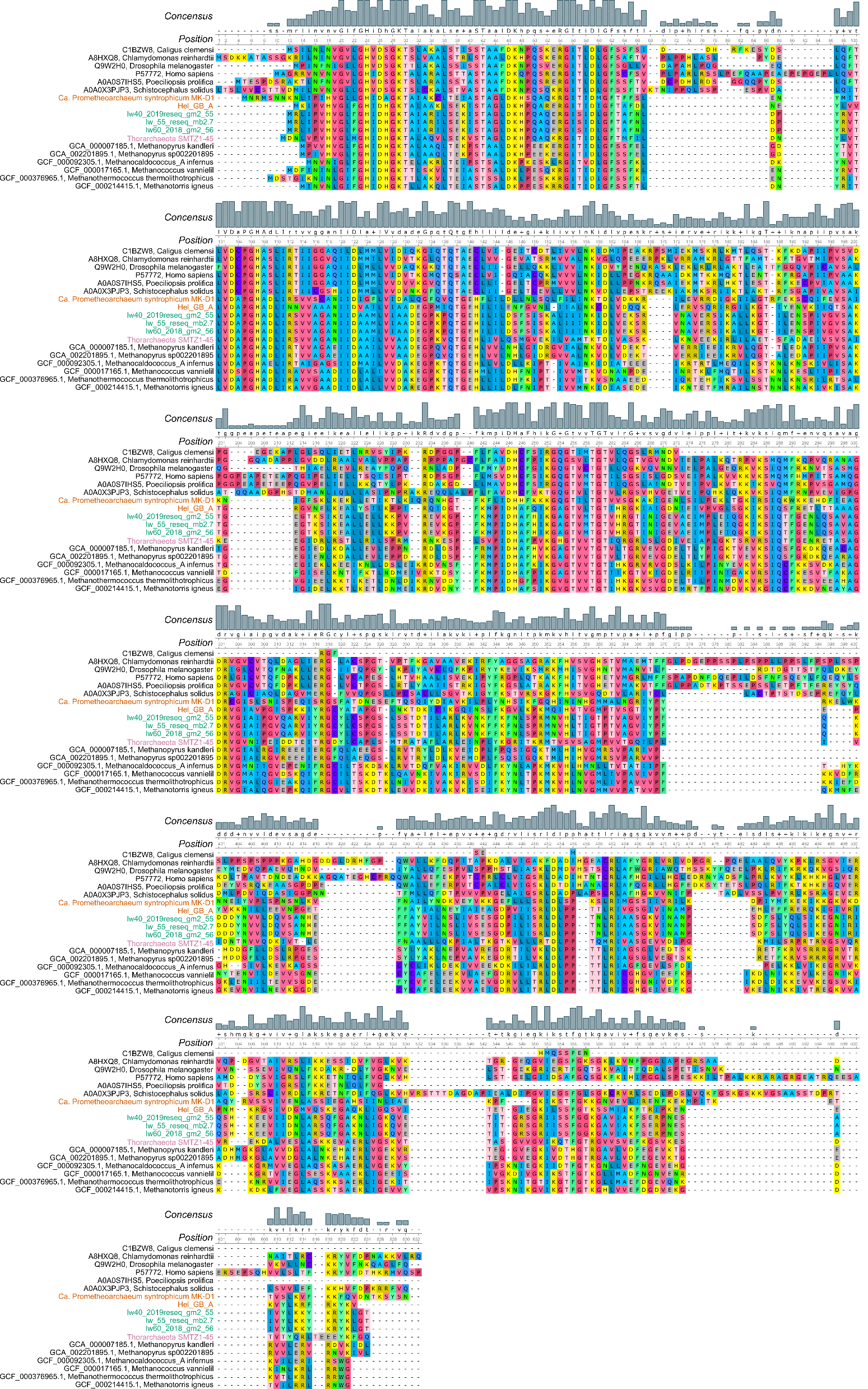
**

**Fig. S21 | Phylogenetic tree and alignment of SelB. a.** Phylogenetic tree of SelB. Tree was inferred with IQ-TREE (LG+C10+F+G+PMSF model) from a Trimal-trimmed (with ‘-automated1’ option) alignment of SelB genes from archaeal (represented by blue branches), bacterial (represented by black branches), and eukaryotic (highlighted with green shade) genomes, and was rooted between the bacterial and archaeal-eukaryotic clade. Tree is based on a 371-position alignment with Bootstrap support values (green numbers under branches) based on 100 IQ-TREE trees. Asgardarchaeota sequences are represented with red clades and individual classes are highlighted with different color labels: Bright cyan - Sifarchaeia; dark yellow - Jordarchaeia; Light pink - Thorarchaeia; orange - Lokiarchaeia. One copy of selB genes in Thorarchaeota SMTZ1-83 clustered with bacterial sequences, indicating contamination. **b.** Alignment of SelB. The alignment includes six eukaryotic selB homologs, six archaeal non-Asgardarchaeota selB homologs, and six Asgardarchaeota selB homologs (highlighted with colours correlating to the colour scheme in the selB tree in Fig. S21a). Sequences were aligned using Muscle with default settings.

**
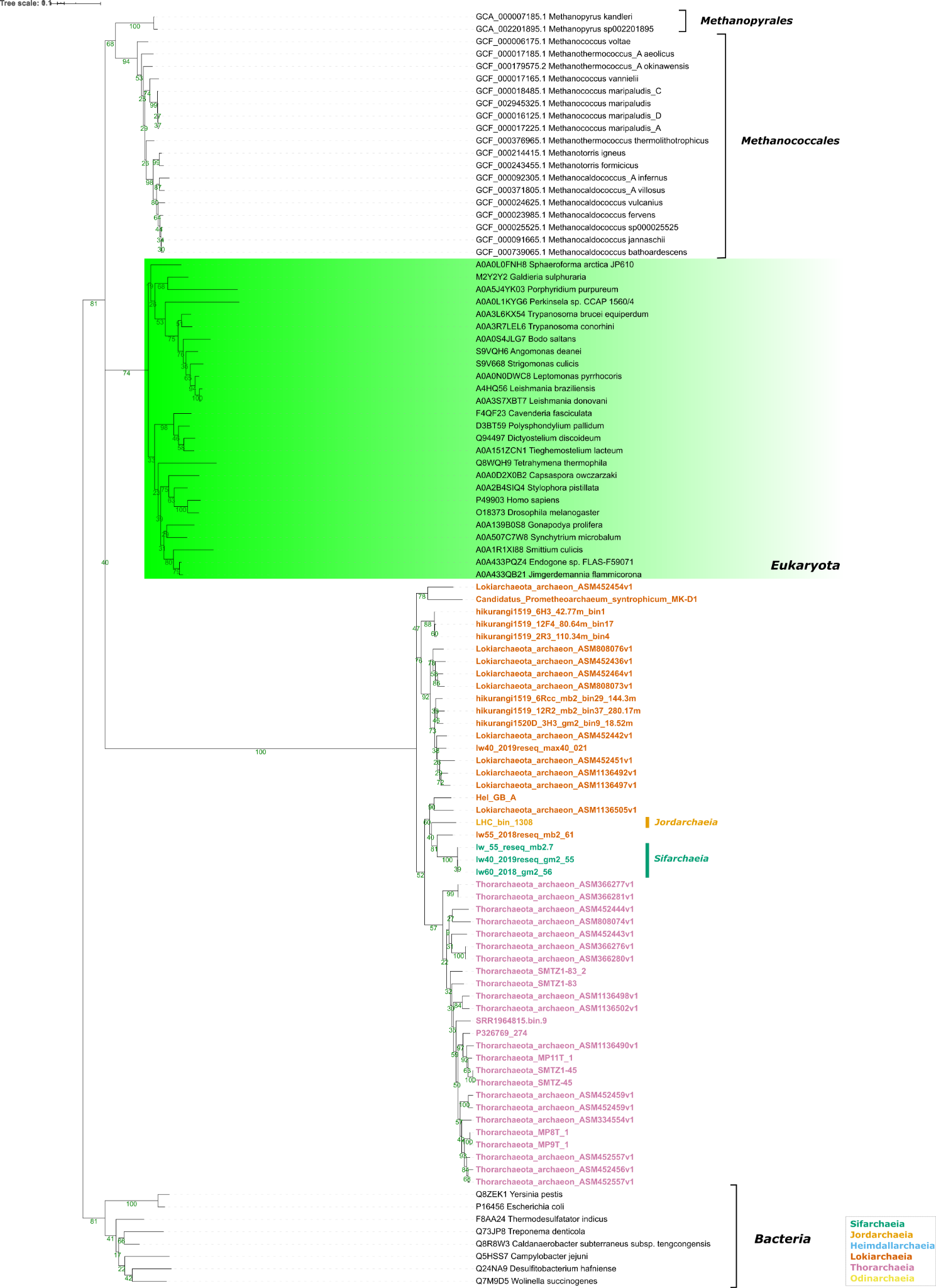
**

**Fig. S22 | Phylogenetic tree of SPS.** Tree was inferred with IQ-TREE (LG+C10+F+G+PMSF model) from a Trimal-trimmed (with ‘-automated1’ option) alignment of SPS/SelD genes from archaeal (represented by blue branches), bacterial (represented by black branches), and eukaryotic (highlighted with green shade) genomes, and rooted at the bacterial node. Tree is based on a 448-position alignment with Bootstrap support values (green numbers under branches) based on 100 IQ-TREE trees. Asgardarchaeota sequences are represented with red clades and individual classes are highlighted with different color labels: Bright cyan - Sifarchaeia; dark yellow - Jordarchaeia; Light pink - Thorarchaeia; orange - Lokiarchaeia.


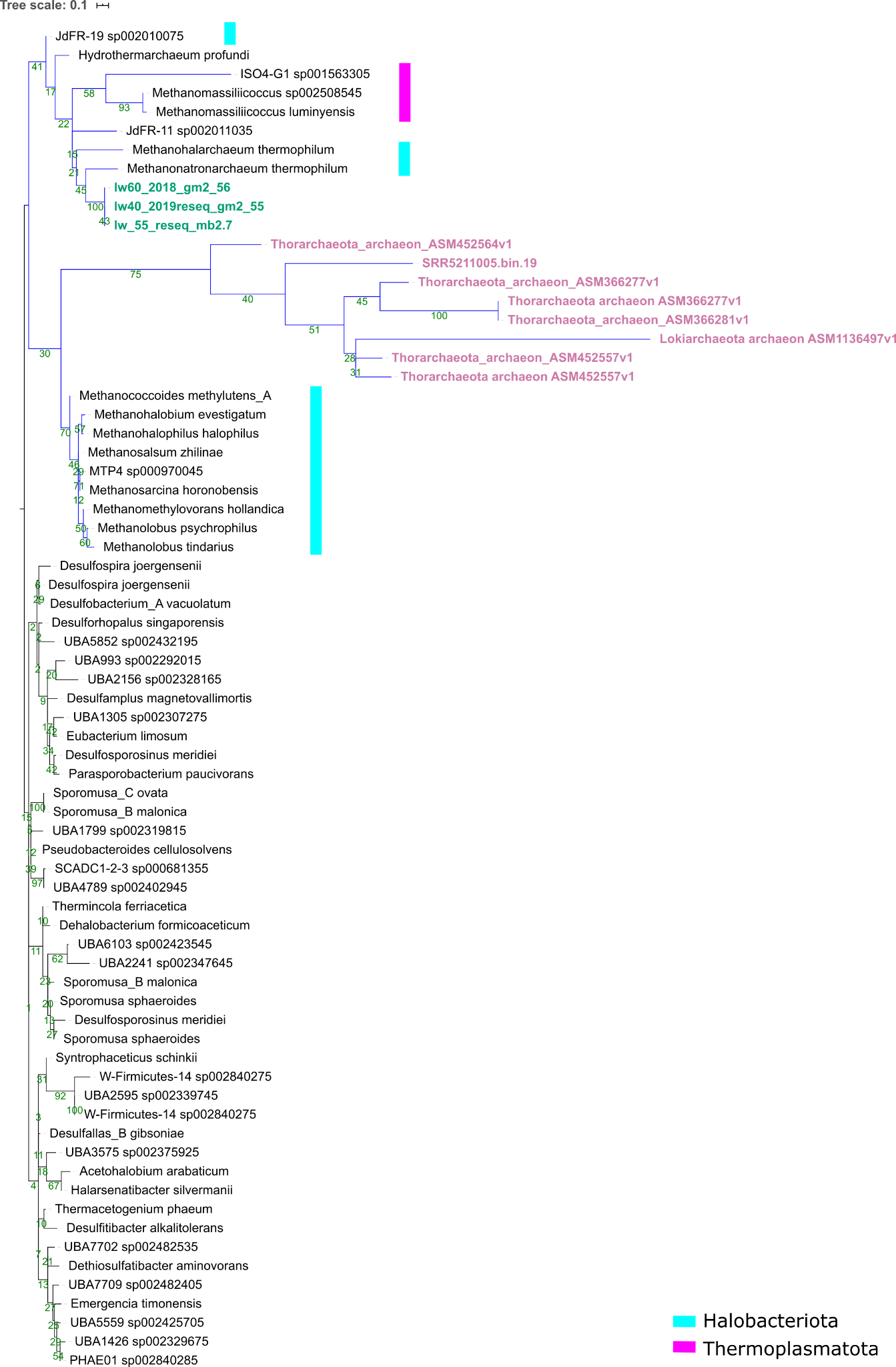


**Fig. S23 | Phylogenetic tree of pyrrolysine tRNA (tRNA-Pyl) sequences.** Tree was inferred with IQ-TREE (TPM3+F+G4) from an untrimmed alignment, and rooted at the bacterial node. Tree is based on a 100-position alignment with bootstrap support values (green numbers under branches) based on 100 IQ-TREE trees. Archaeal sequences were indicated by blue branches. Sifarchaeia and Thorarchaeia are highlighted with bold labels in green and light pink.


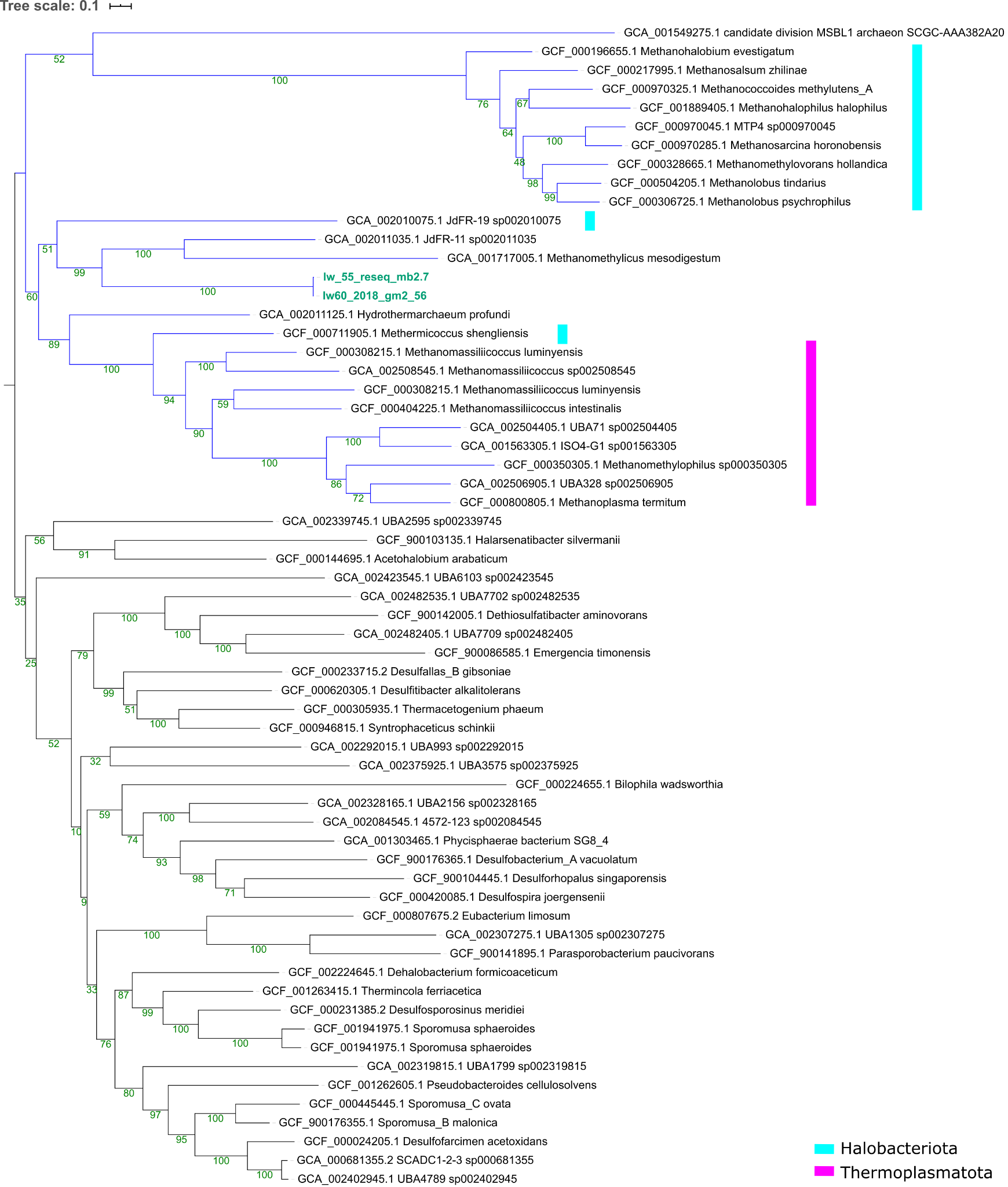


**Fig. S24 | Phylogenetic tree of PylSBCD.** The concatenated alignment (1103 columns) of pylS, pylB, pylC and pylD genes from archaeal (represented by blue branches), and bacterial (represented by black branches) genomes were trimmed using TrimAl with ‘-automated1’ option and removed columns of less than 40% consensus. Tree was inferred with IQ-TREE (LG+C10+F+G+PMSF model) and rooted at the bacterial node. Bootstrap support values (green numbers under branches) were calculated based on 100 IQ-TREE trees under the same model. Sifarchaeia sequences are represented with bold labels in green.


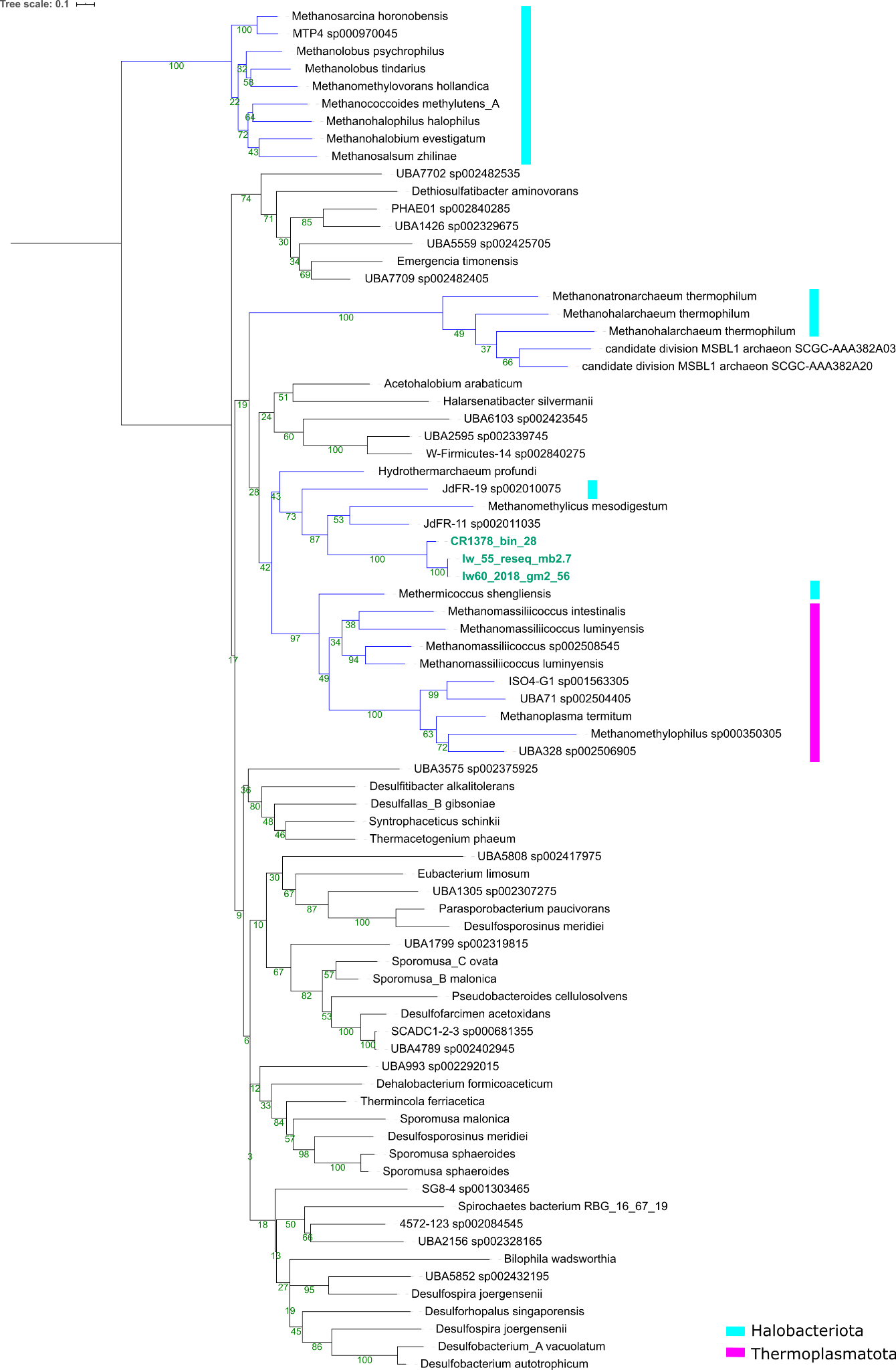


**Fig. S25 | Phylogenetic tree of PylB.** Tree was inferred with IQ-TREE (LG+C60+F+G+PMSF model) from a Trimal-trimmed (with ‘-automated1’ option) alignment of pylB gene from archaeal (represented by blue branches), and bacterial (represented by black branches) genomes, and was rooted on the Halobacteriota family Methanosarcinaceae (as this is the only stable group across all single Pyl gene trees (**Fig. S25-28**)). Tree is based on a 337-position alignment with Bootstrap support values (green numbers under branches) based on 100 trees under the same model. Sifarchaeia sequences are represented with bold labels in green.


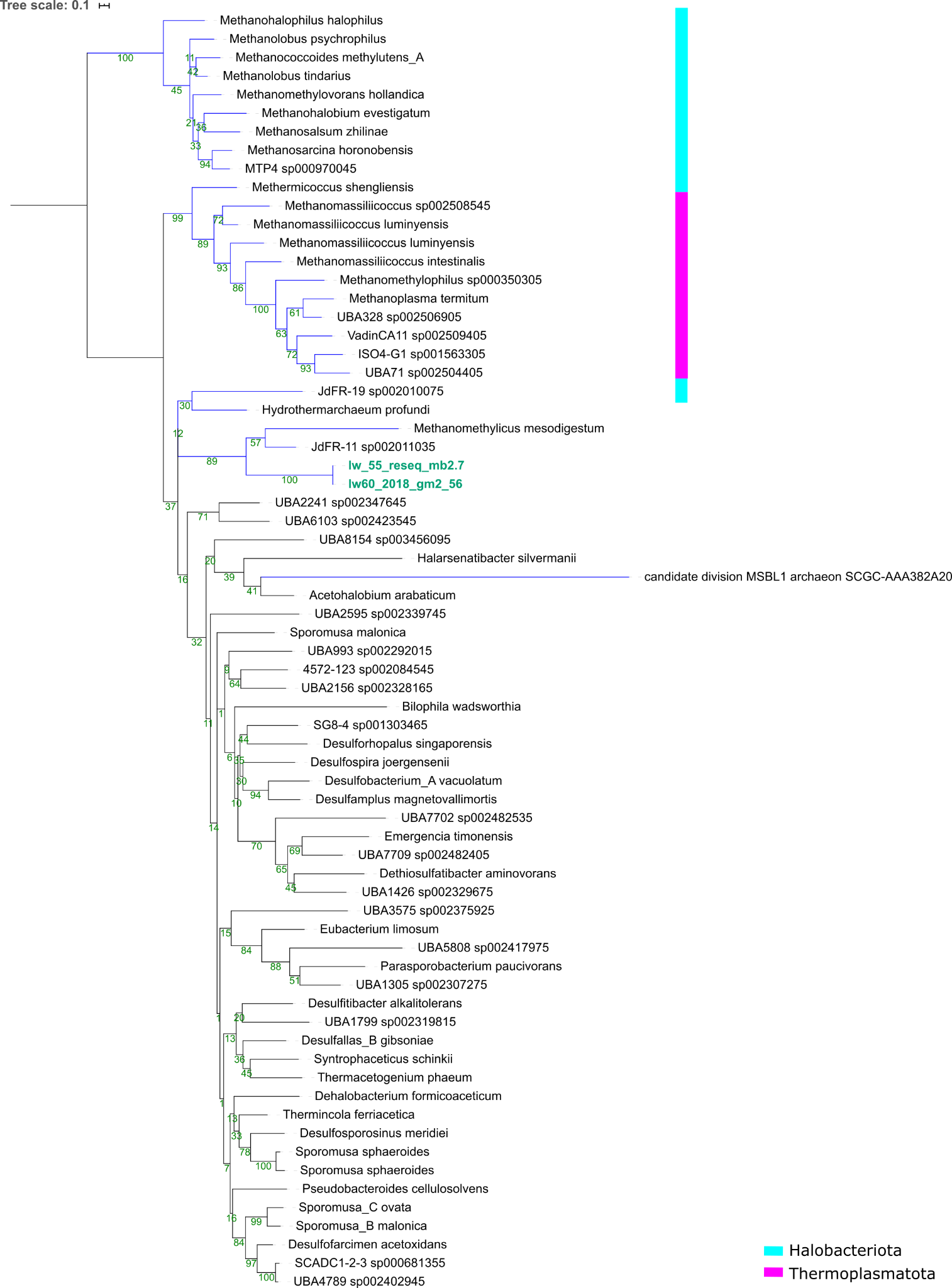


**Fig. S26 | Phylogenetic tree of PylC.** Tree was inferred with IQ-TREE (LG+C60+F+G+PMSF model) from a Trimal-trimmed (with ‘-automated1’ option) alignment of pylC gene from archaeal (represented by blue branches), and bacterial (represented by black branches) genomes, and was rooted on the Halobacteriota family Methanosarcinaceae. Tree is based on a 254-position alignment with Bootstrap support values (green numbers under branches) based on 100 trees under the same model. Sifarchaeia sequences are represented with bold labels in green.

**
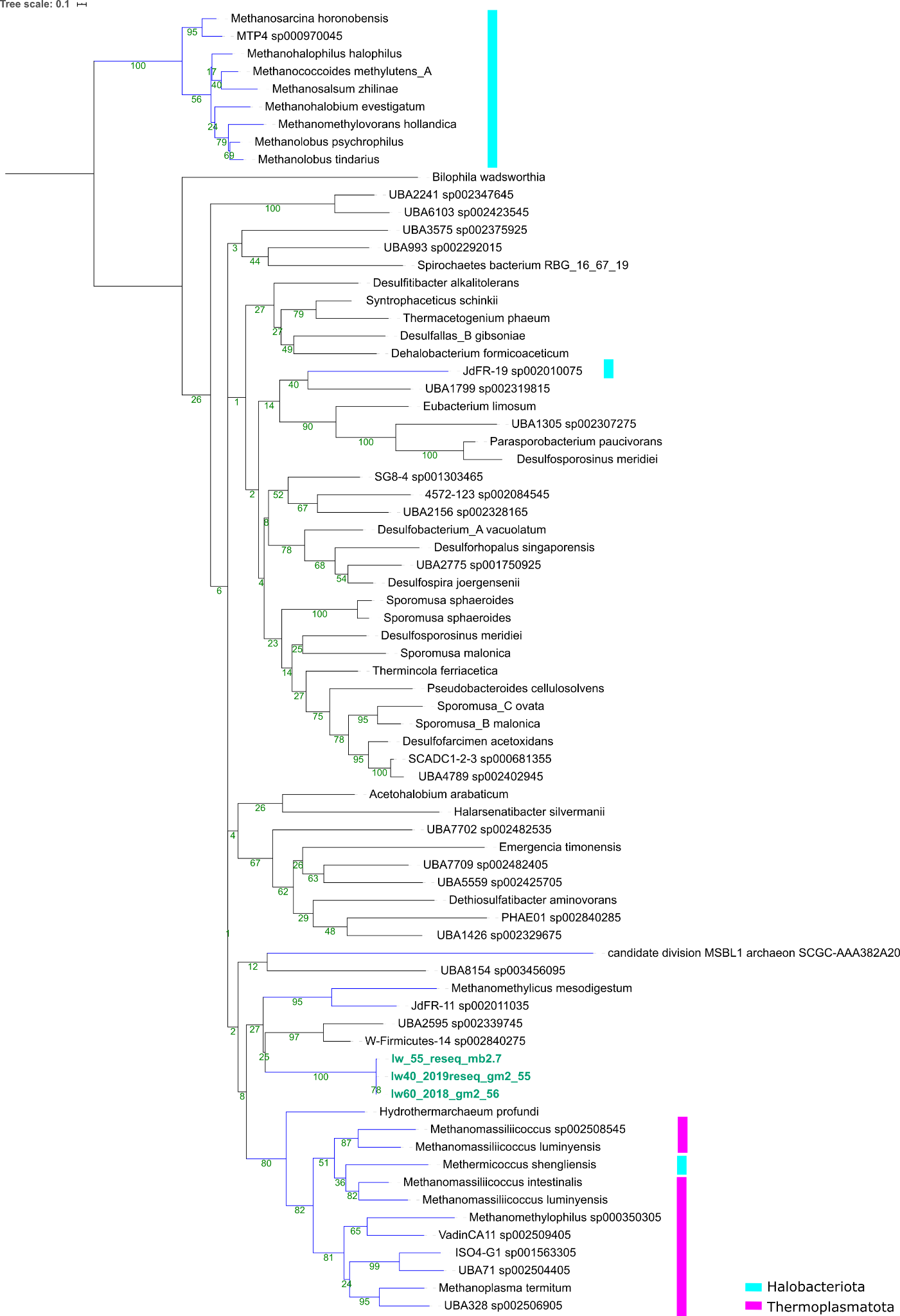
**

**Fig. S27 | Phylogenetic tree of PylD.** Tree was inferred with IQ-TREE (LG+C60+F+G+PMSF model) from a Trimal-trimmed (with ‘-automated1’ option) alignment of pylD gene from archaeal (represented by blue branches), and bacterial (represented by black branches) genomes, and was rooted on the Halobacteriota family Methanosarcinaceae. Tree is based on a 199-position alignment with Bootstrap support values (green numbers under branches) based on 100 trees under the same model. Sifarchaeia sequences are represented with bold labels in green.

**
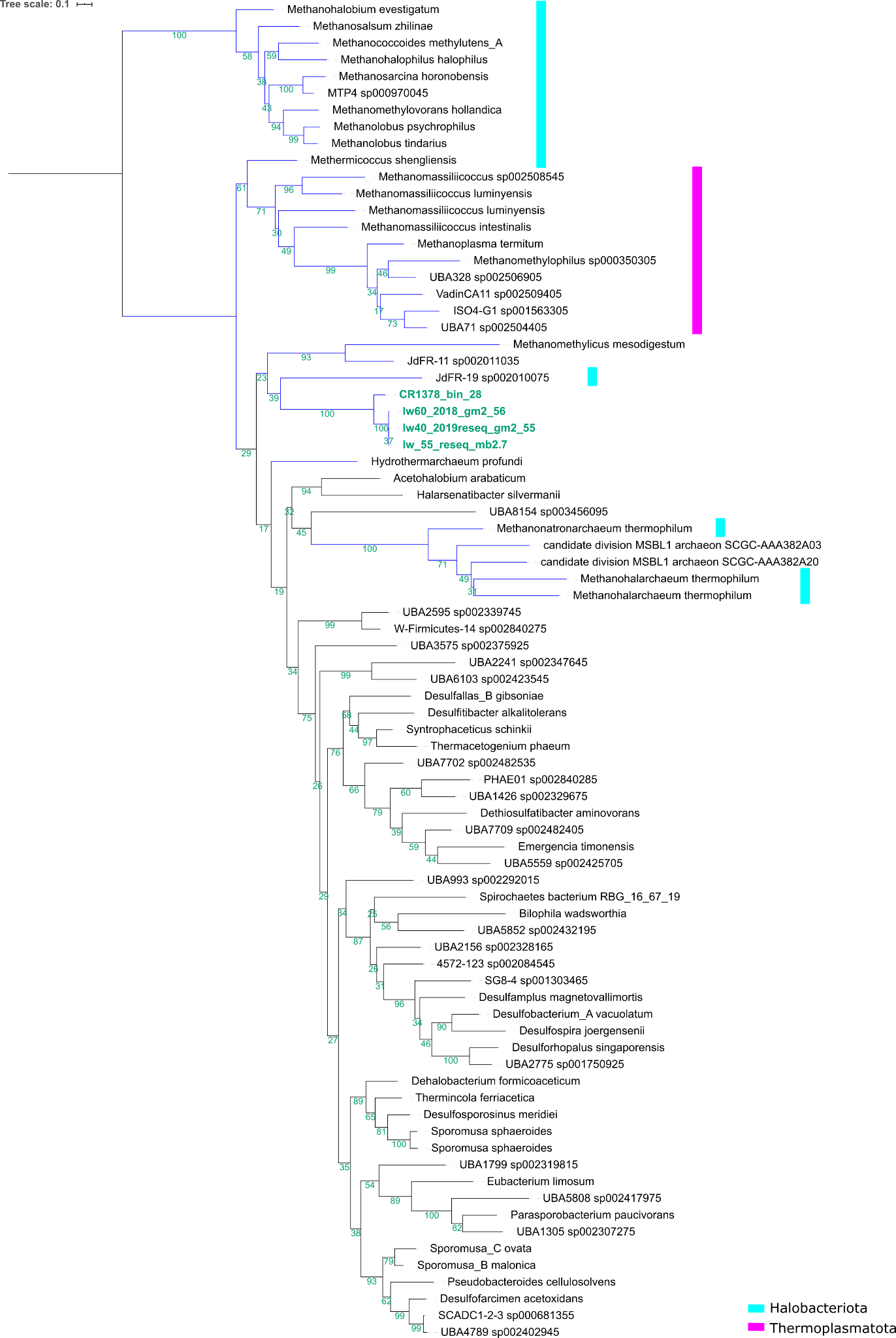
**

**Fig. S28 | Phylogenetic tree of PylS.** Tree was inferred with IQ-TREE (LG+C60+F+G+PMSF model) from a Trimal-trimmed (with ‘-automated1’ option) alignment of pylSnSc genes from archaeal (represented by blue branches), and bacterial (represented by black branches) genomes, and was rooted on the Halobacteriota family Methanosarcinaceae. Tree is based on a 320-position alignment with Bootstrap support values (green numbers under branches) based on 100 trees under the same model. Sifarchaeia sequences are represented with bold labels in green.

**(a)
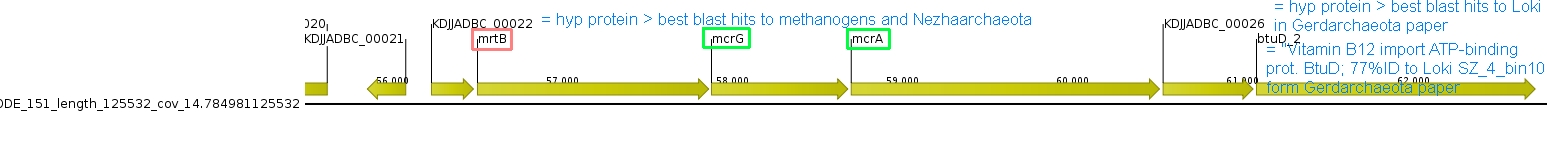
**

**(b)**

**
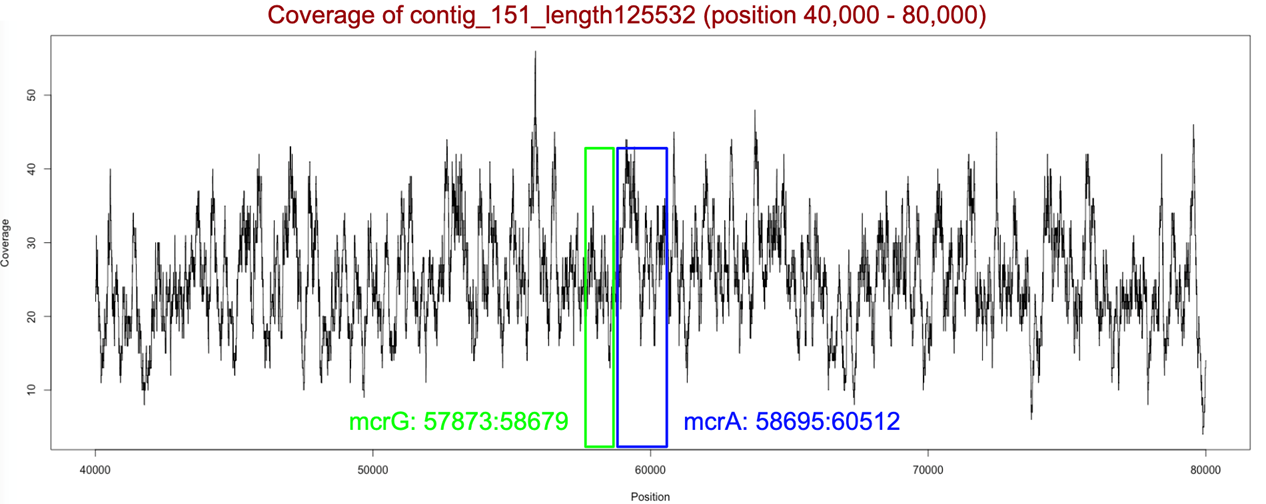
**

**(c)
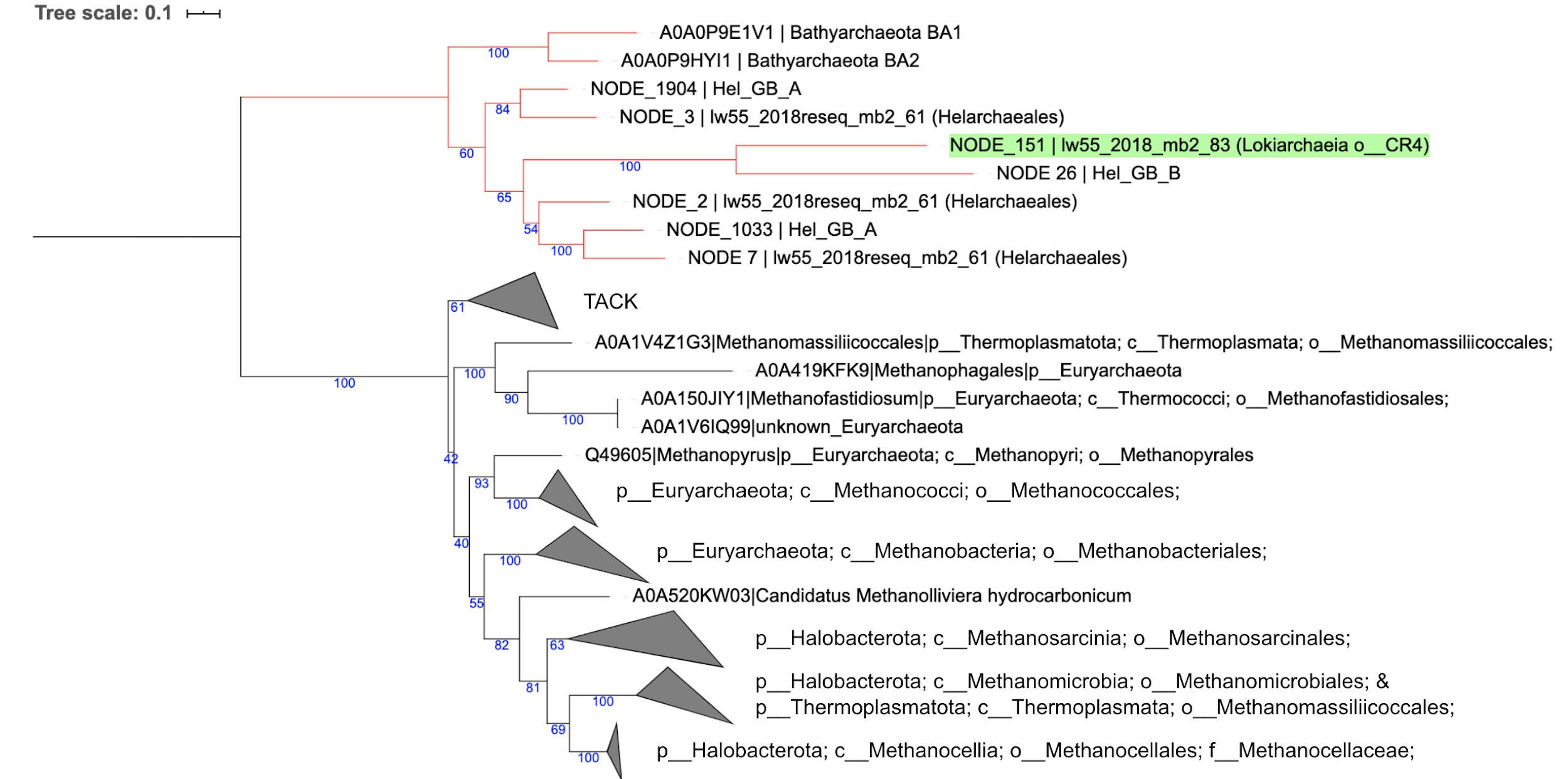
**

**(d)**

**
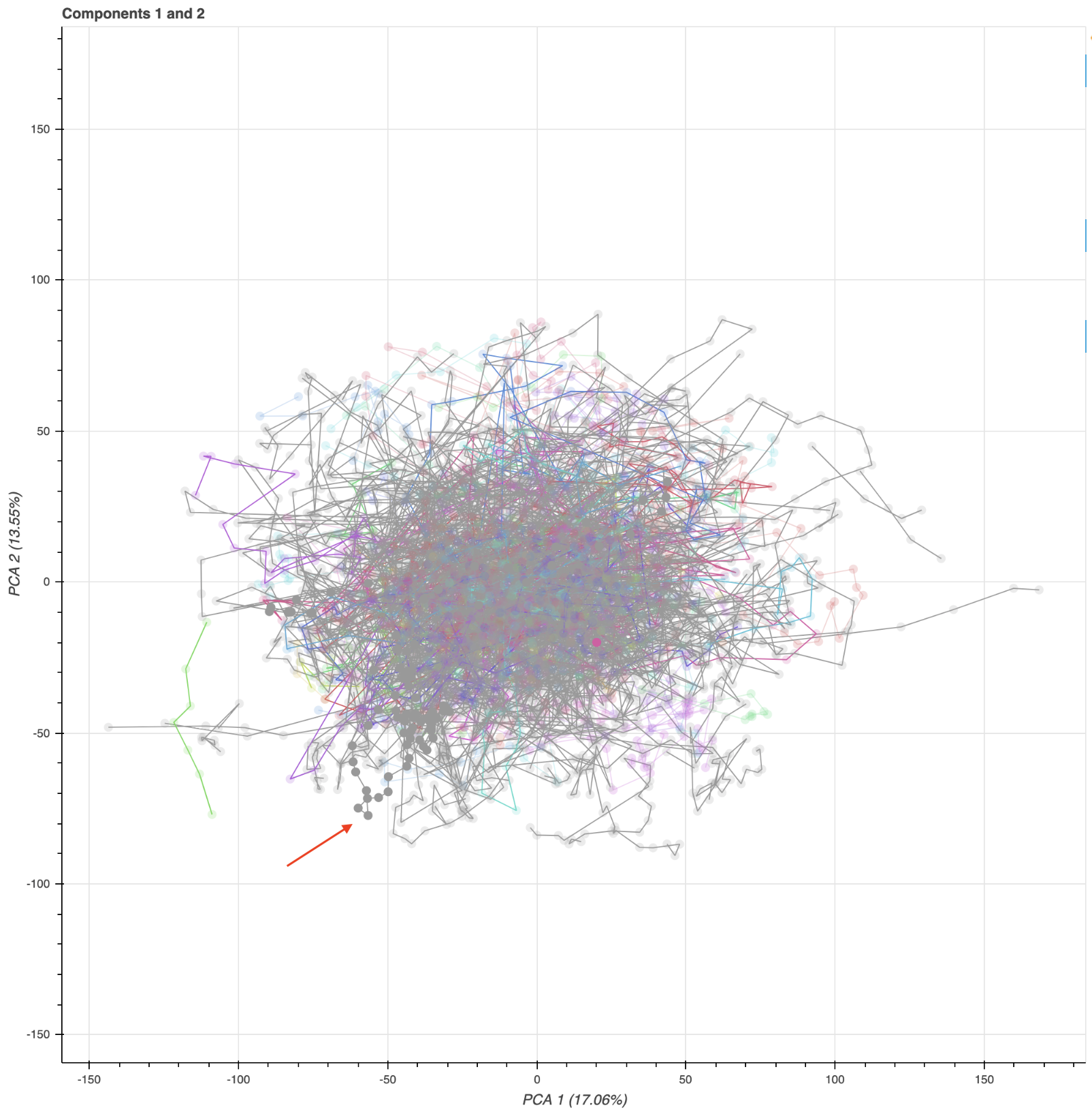

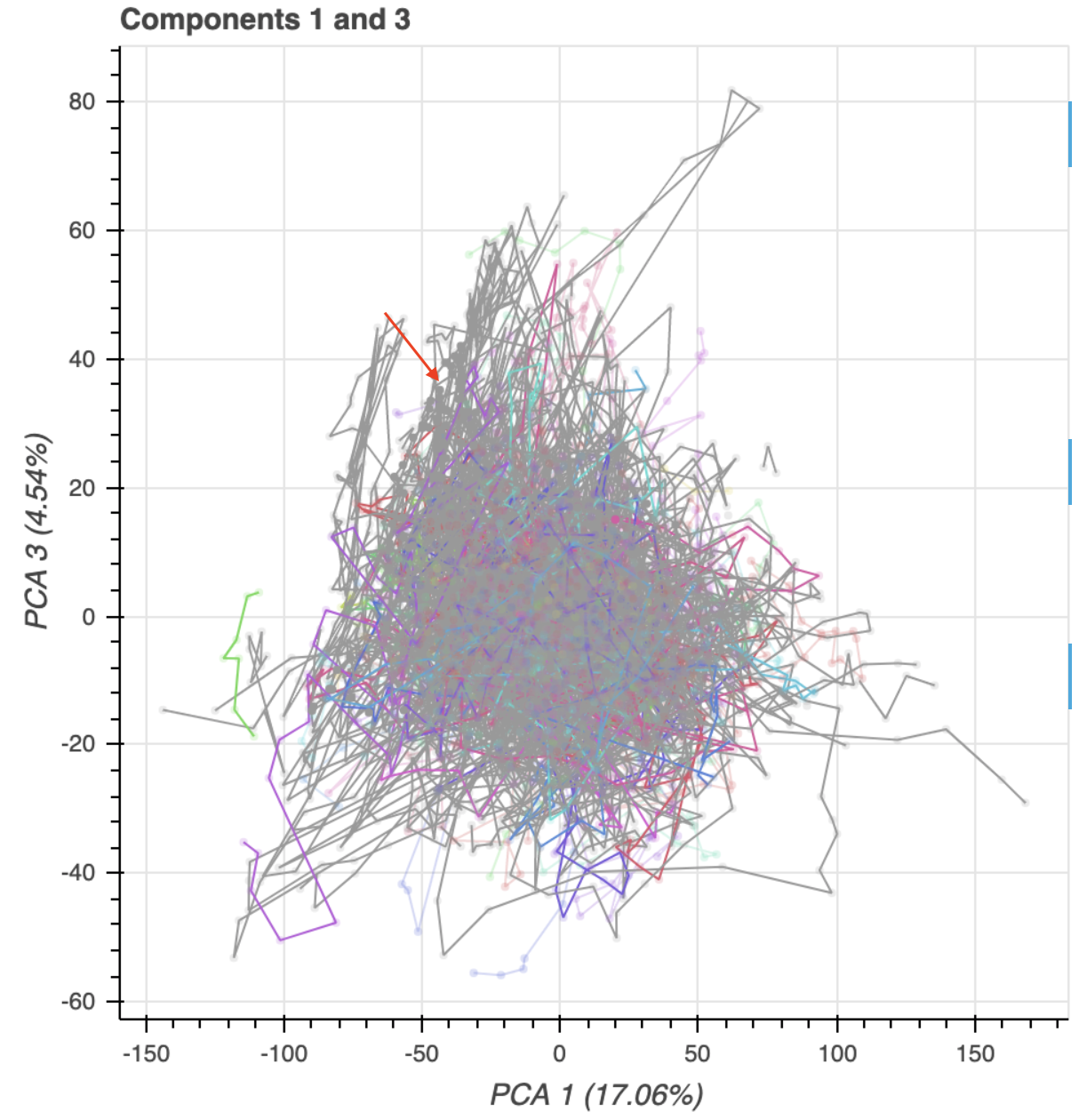

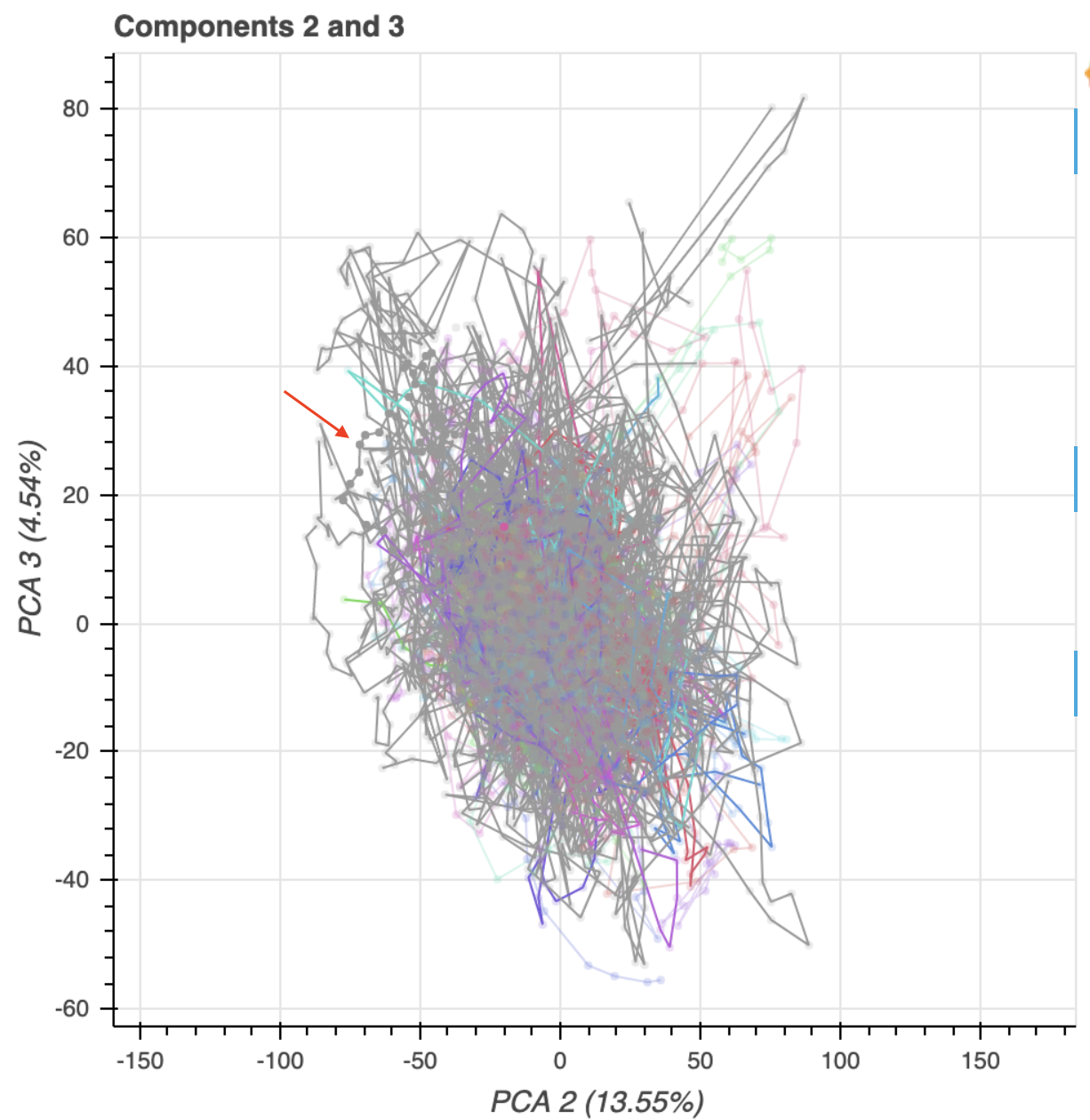
**

**(e)**

**
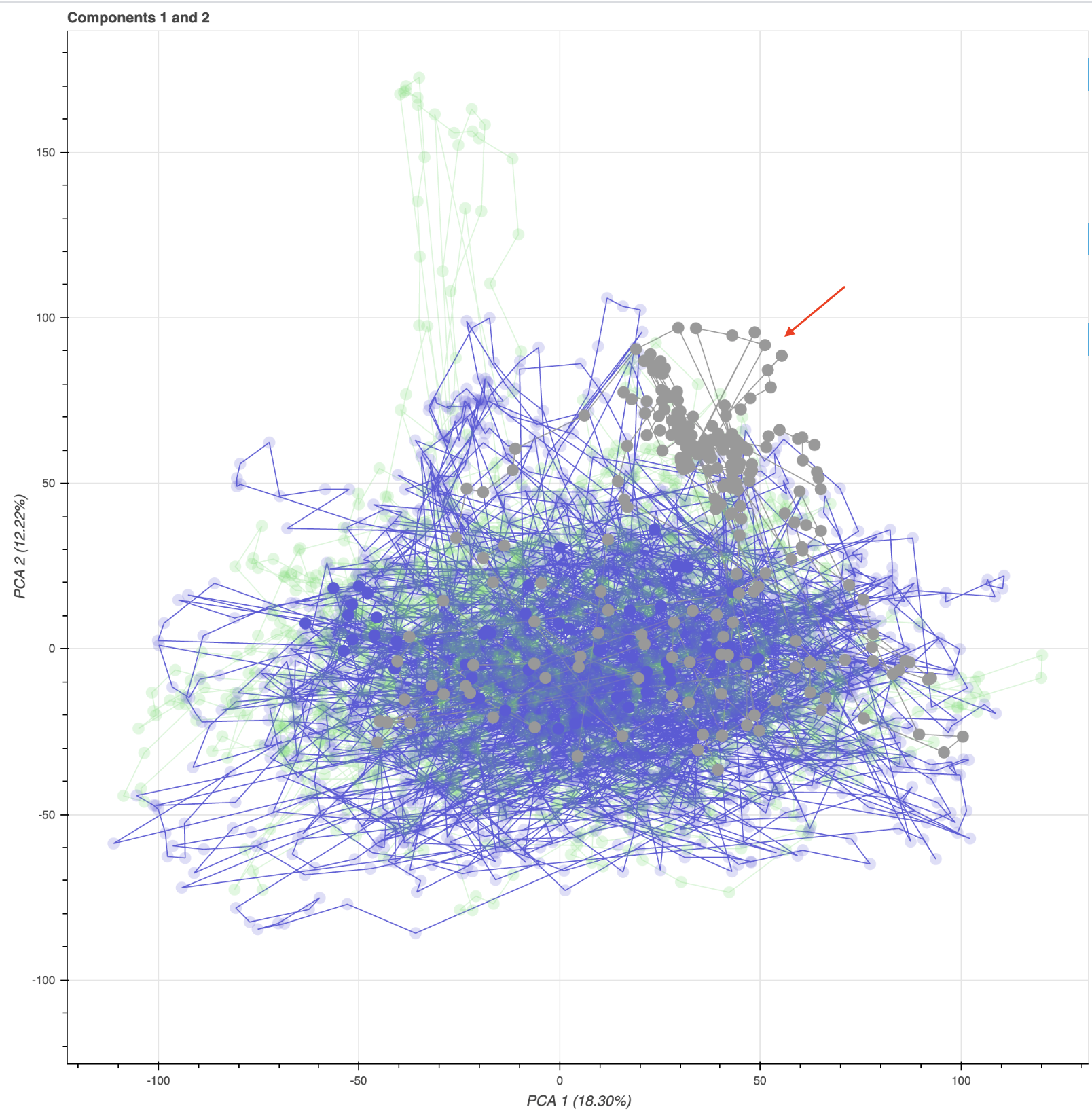

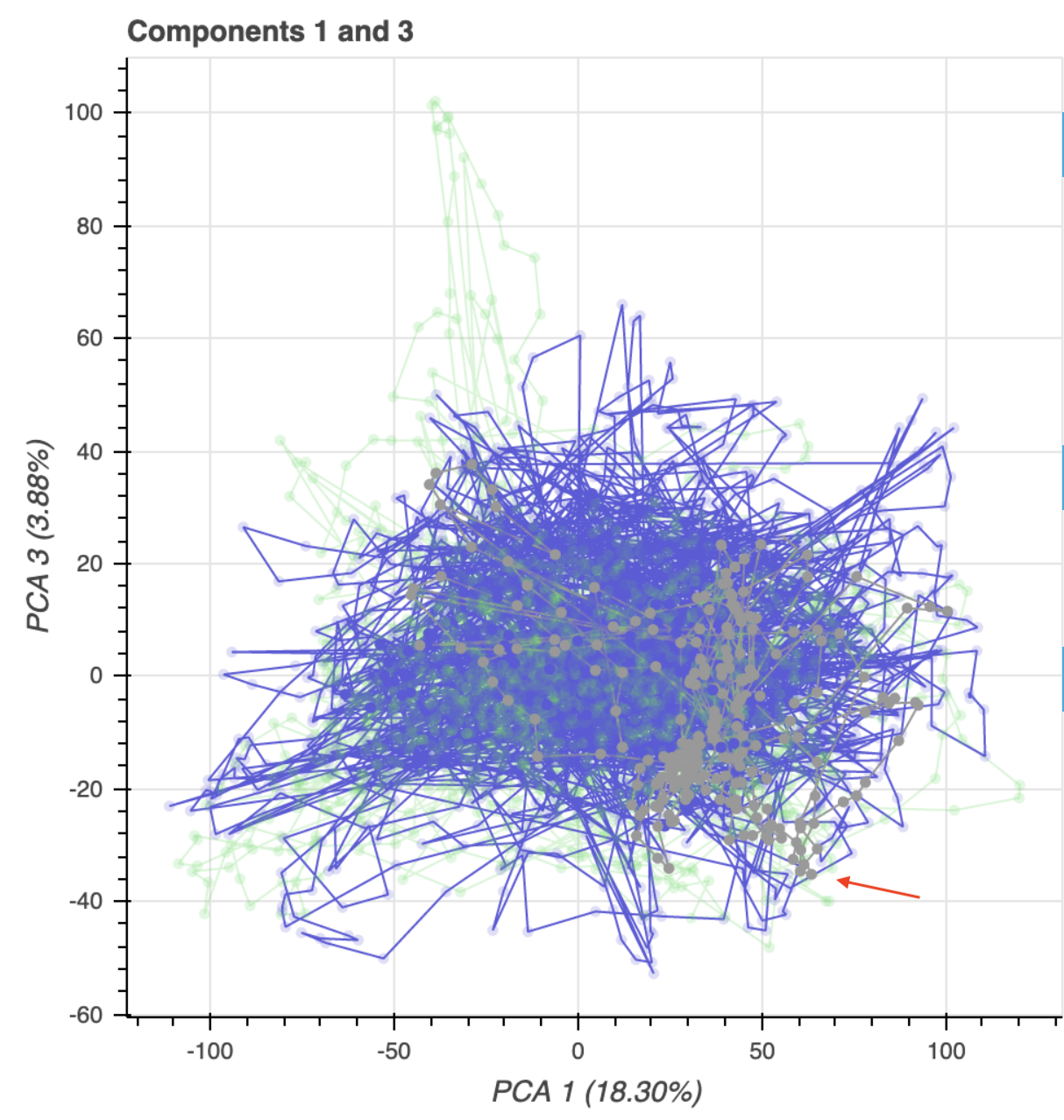

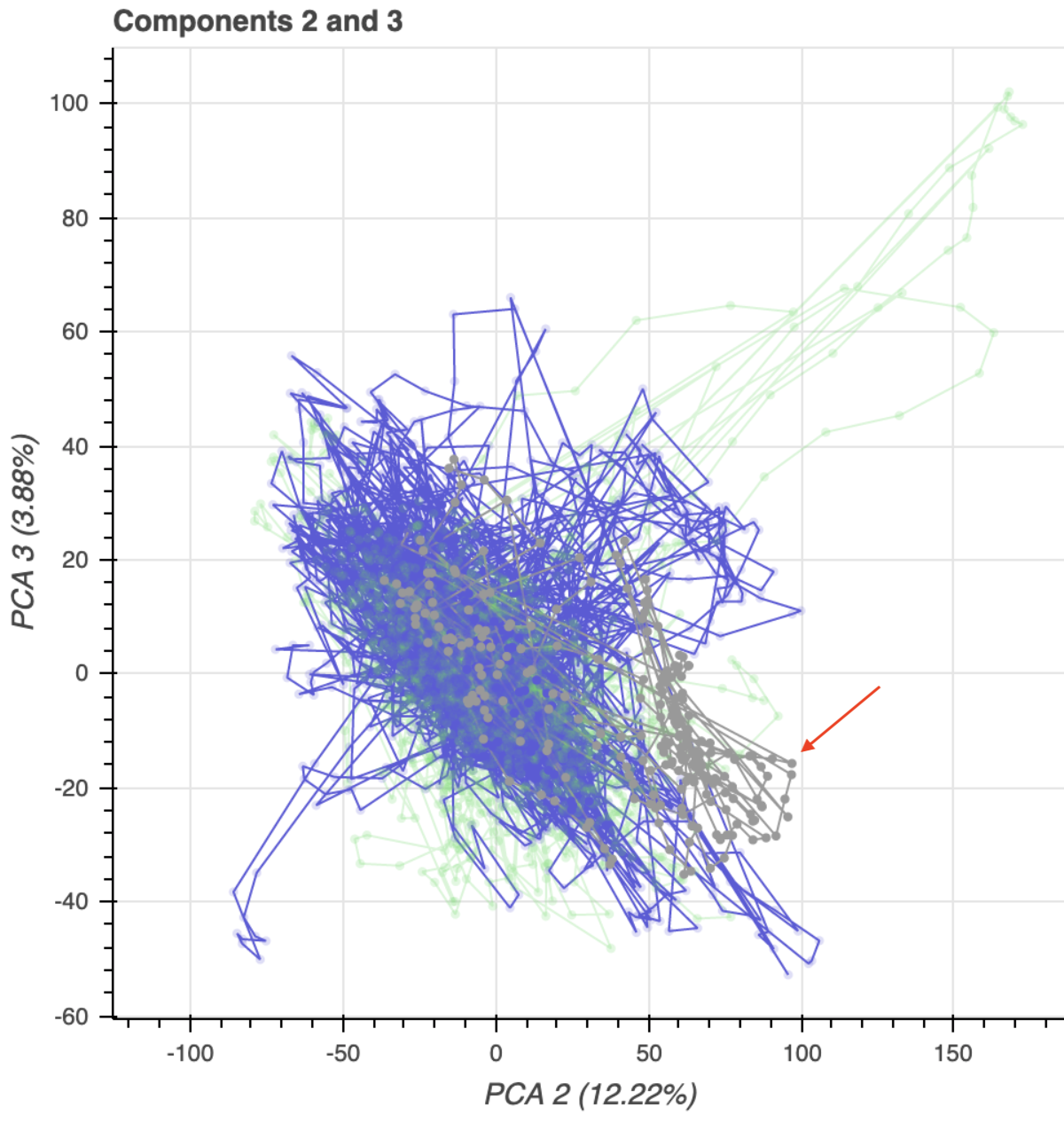
**

**Fig. S29 | Methyl coenzyme M reductase (mcr) genes in MAG lw55_2018_mb2_83. a.** Mcr gene neighbourhood. The taxonomy of the blastp top hit is provided for each gene. **b.** Coverage plot - contig NODE_151 in lw55_2018_mb2_83. **c.** Phylogenetic tree of mrcA inferred with IQTREE using LG+C10+F+G+PMSF model based on an alignment of columns. This tree shows that the mcrA gene detected in the Lokiarchaeia bin (lw55_2018_mb2_83, highlighted in green) clusters with a sequence from the Hel_GB_B MAG, which was described as one of the original Helarchaeales MAGs (Seitz et al., 2019). **d.** and **e.** K-mer frequency analysis of mcr-encoded contigs in lw55_2018_mb2_83 (Lokiarchaeia) and lw55_2018reseq_mb2_61 (Helarchaeales). Shown are the PCA components 1 vs 2, 1 vs 3, and 2 vs 3. The highlighted grey dots connected by grey lines in **d** and **e** represent contig NODE_151 in lw55_2018_mb2_83.


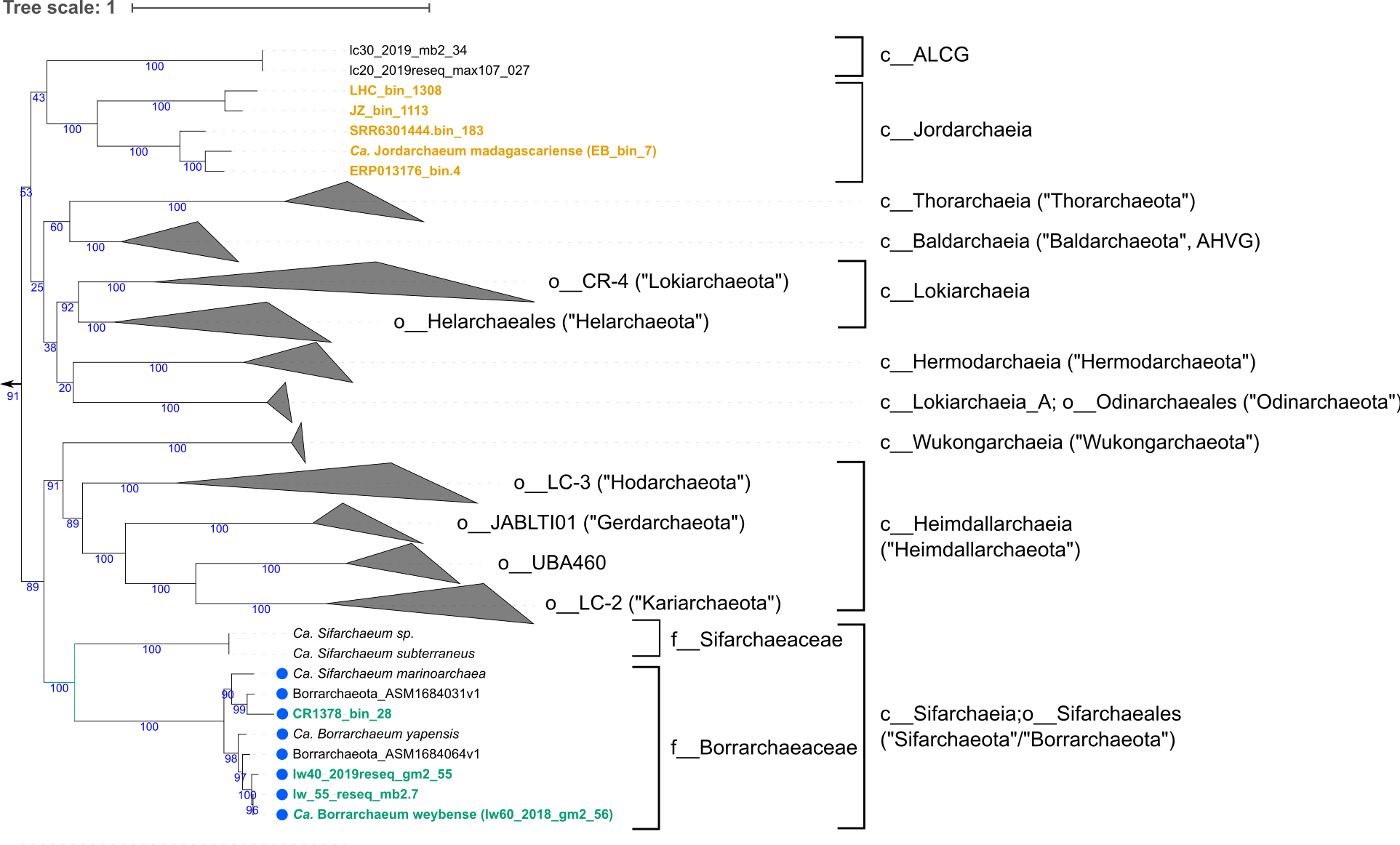


**Fig. S30 | Asgardarchaeota taxonomy including recently published lineages.** The underlying phylogeny was inferred with IQTREE (C10, PMSF) from a concatenated protein sequence alignment of 53 archaeal markers. Our result supports the rank of class for four of the recently proposed lineages, the “Hermodarchaeota” (Liu et al., 2021; Zhang et al., 2021), “Sifarchaeota” (Farag et al., 2021), “Baldarchaeota” and “Wukongarchaeota” (Liu et al., 2021), here referred to as Hermodarchaeia Sifarchaeia, Baldarchaeia, and Wukongarchaeia. The remaining recently proposed lineages “Kariarchaeota”, “Hodarchaeota”, and “Borrarchaeota” (Liu et al., 2021), were placed within these classes and represent the orders Kariarchaeales (LC-2), and Hodarchaeales (LC-3) in Heimdallarchaeia, and the family Borrarchaeaceae within Sifarchaeia, respectively.

Note, that one of the classes proposed in this manuscript (indicated by green genome bin names) is synonymous with the recently proposed lineages “Sifarchaeota” and “Borrarchaeota”. Due to its publication priority, we have used Sifarchaeota as the base name, noting that this lineage represents a class (Sifarchaeia; proposed in this study) according to rank normalisation, which we use throughout this manuscript. We also propose the intermediate ranks of family and order, and a corrected spelling of the genus *Ca.* Sifarchaeotum, i.e. *Ca.* Sifarchaeum, as well as the species *Ca.* Borrarchaeum weybense (lw60_2018_gm2_56). Further note, that we detected pyrrolysine recoding in other members of the family Borrarchaeaceae (blue dots), but not in the two genomes representing the family Sifarchaeaceae. Arrow points to outgroup of 10 archaeal genomes sampled across the archaeal domain.

**References**

Coleman, G.A., Pancost, R.D., and Williams, T.A. (2019). Investigating the Origins of Membrane Phospholipid Biosynthesis Genes Using Outgroup-Free Rooting. Genome Biol Evol *11*, 883–898.

Seitz, K.W., Dombrowski, N., Eme, L., Spang, A., Lombard, J., Sieber, J.R., Teske, A.P., Ettema, T.J.G., and Baker, B.J. (2019). Asgard archaea capable of anaerobic hydrocarbon cycling. Nature Communications *10*, 1822.

Spang, A., Stairs, C.W., Dombrowski, N., Eme, L., Lombard, J., Caceres, E.F., Greening, C., Baker, B.J., and Ettema, T.J.G. (2019). Proposal of the reverse flow model for the origin of the eukaryotic cell based on comparative analyses of Asgard archaeal metabolism. Nature Microbiology 1.

Welte, C., and Deppenmeier, U. (2011). Membrane-Bound Electron Transport in Methanosaeta thermophila▿. J Bacteriol *193*, 2868–2870.

Widmann, J., Harris, J.K., Lozupone, C., Wolfson, A., and Knight, R. (2010). Stable tRNA-based phylogenies using only 76 nucleotides. RNA *16*, 1469–1477.

Yu, H., Wu, C.-H., Schut, G.J., Haja, D.K., Zhao, G., Peters, J.W., Adams, M.W.W., and Li, H. (2018). Structure of an Ancient Respiratory System. Cell *173*, 1636-1649.e16.

Dym, O., Pratt, E.A., Ho, C., and Eisenberg, D. (2000). The crystal structure of D-lactate dehydrogenase, a peripheral membrane respiratory enzyme. Proc Natl Acad Sci U S A *97*, 9413–9418.

Katoh, K., and Standley, D.M. (2013). MAFFT Multiple Sequence Alignment Software Version 7: Improvements in Performance and Usability. Mol Biol Evol *30*, 772–780.

Mariotti, M., Lobanov, A.V., Guigo, R., and Gladyshev, V.N. (2013). SECISearch3 and Seblastian: new tools for prediction of SECIS elements and selenoproteins. Nucleic Acids Res *41*, e149.

Okonechnikov, K., Golosova, O., Fursov, M., and the UGENE team (2012). Unipro UGENE: a unified bioinformatics toolkit. Bioinformatics *28*, 1166–1167.

Farag, I.F., Zhao, R., and Biddle, J.F. (2021). “Sifarchaeota” a novel Asgard phylum from Costa Rica sediment capable of polysaccharide degradation and anaerobic methylotrophy. Appl. Environ. Microbiol.

Liu, Y., Makarova, K.S., Huang, W.-C., Wolf, Y.I., Nikolskaya, A.N., Zhang, X., Cai, M., Zhang, C.-J., Xu, W., Luo, Z., et al. (2021). Expanded diversity of Asgard archaea and their relationships with eukaryotes. Nature 1–5.

Zhang, J.-W., Dong, H.-P., Hou, L.-J., Liu, Y., Ou, Y.-F., Zheng, Y.-L., Han, P., Liang, X., Yin, G.-Y., Wu, D.-M., et al. (2021). Newly discovered Asgard archaea Hermodarchaeota potentially degrade alkanes and aromatics via alkyl/benzyl-succinate synthase and benzoyl-CoA pathway. The ISME Journal 1–18.
