# Supplementary material for: Recoding of stop codons expands the metabolic potential of two novel Asgardarchaeota lineages
Source: ISME Commun. 2021 Jun 28;1:30. doi: 10.1038/s43705-021-00032-0 (PMC9723677; doi:10.1038/s43705-021-00032-0)
Supplement: Supplementary file 3 — Supplementary Text [file 43705_2021_32_MOESM3_ESM.docx]

**Supplementary Text**

**Vitamin biosynthesis**

Similar to the Lokiarchaeia culture Ca. *Prometheoarchaeum syntrophicum strain MK-D1* genome, the Sifarchaeia and Jordarchaeia MAGs do not encode complete biosynthesis pathways for several vitamins including cobalamin (vitamin B12), peridoxin (vitamin B6), and biotin (vitamin B7) (**Table S9**). We therefore concluded that these vitamins could be provided by a syntrophic partner (see main text).

**Methylamine utilisation in Sifachaeia**

Previously reported Pyl-containing proteins include dimethylamine methyltransferase (MtbB), trimethylamine methyltransferase (MttB), and monomethylamine methyltransferase (MtmB). In all three cases Pyl is located at the active sites of the enzyme and therefore Pyl is regarded as being essential for methylamine utilisation and the methyl transfer to coenzyme M under anoxic conditions [[1]](https://www.zotero.org/google-docs/?RSJZxn).

Sifarchaeia genomes encode the methyltransferases MtbB and MtmB in MAGs lw55 and lw60, and MtmB in MAG lw40. Screening for Pyl-containing genes by applying a previously published approach [[2]](https://www.zotero.org/google-docs/?KkyIny), we identified MtbB, encoding dimethylamine methyltransferase (DMA) as the only encoded Pyl containing protein in Sifarchaeia. The presence of the subunits mtbA and mtbC suggests that Sifarchaeia are able to incorporate Pyl to transfer the methyl group from dimethylamine to coenzyme M and a corrinoid protein.

Note that we did not detect the MtbB gene in two of the four Sifarchaeia MAGs (lw40_2019reseq_gm2_55 and CR1378_bin_28) which represent less complete genomes with 52.44% and 47.38% estimated completeness, respectively. It is therefore likely that the MtbB gene is missing from these MAGs due to the low completeness.

To verify that the mtmB gene in Sifarchaeia includes indeed no Pyl recoding, we compared gene lengths and searched for TAG codons in regions of 300bp upstream and downstream of each predicted mtmB gene. We found that the average length of the predicted Sifarchaeia mtmB genes is 450bp, which covers the full HMM length of pyl-encoded mtmB genes in the Pfam database, making it unlikely that the gene length was predicted incorrectly and that possible Pyl codons were missed. These results suggest that Sifarchaeia mtmB genes do not contain Pyl and therefore could perform functions other than methyl transfer from monomethylamines.
Examples of alternative functions of methylamine targeting enzymes are known for trimethylamine methyltransferase (MttB) which has been reported from lineages lacking Pyl stop codon recoding.

For example, a non-Pyl mttB homolog has been reported for *Desulfitobacterium hafniense*, and found to encode an enzyme catalysing the transfer of methyl groups from trimethylglycine rather than trimethylamine [[3]](https://www.zotero.org/google-docs/?KcR5mB).

**Methyl transfer to and from coenzyme M**

Methanogens use an eight-subunit membrane-associated complex (MtrABCDEFGH) that couples the transfer of a methyl group from H_4_MPT to coenzyme M with the translocation of a sodium ion across the cell membrane [[4]](https://www.zotero.org/google-docs/?DGJQYO). Genomes encoding only two subunits, the methyltransferase (MtrH) catalysing the transfer of the methyl group from H_4_MPT, and MtrA a corrinoid-harboring protein supplying the methyl group to CoM [[5]](https://www.zotero.org/google-docs/?pwlhbw) have also been reported for Methanomassiliicoccales [[6]](https://www.zotero.org/google-docs/?cbuU7X). The authors of this study proposed that mtrAH encodes a H_4_F/H_4_MPT-CoM methyltransferase. Likewise, we found genes only encoding the two catalytic subunits (MtrAH) in Sifarchaeia and Jordarchaeia (**Table S9**). We thus speculate that both novel lineages might use this enzyme to catalyse the reverse reaction to facilitate the transfer of methyl groups from methyl-CoM to methyl-H_4_MPT, and subsequently to acetyl-coenzyme A (CoA) to be reduced to acetate for energy conservation (**Fig. 2**; main text).

**Coenzyme B, coenzyme M, and tetrahydromethanopterin biosynthesis**

We detected key genes in the coenzyme B (CoB), coenzyme M (CoM), and tetrahydromethanopterin (H_4_MPT) biosynthesis pathway (**Table S9**).

We identified methanogen homocitrate synthase (aksA) genes in three Sifarchaeia MAGs and methanogen homoaconitase (aksD) genes in Jordarchaeia JZ_BIN_1113, both of which are key genes in the CoB biosynthesis pathway. Missing key genes include EC 4.2.1.114 for methanogen homoaconitase that catalyzes trihomocitrate to homoisocitrate, and aksF for methanogen homoisocitrate dehydrogenase, oxidizing homoisocitrate to 2-oxosubutyrate. Genes encoding enzymes catalyzing the last four steps in the CoB biosynthesis using the substrate 2-oxosuberate and ATP are still uncharacterised.

Key genes in CoM biosynthesis pathway, including phosphosulfolactate synthase (comA), 2-phosphosulfolactate phosphatase (comB), L-2-hydroxycarboxylate dehydrogenase (NAD+) (ComC), and sulfopyruvate decarboxylase (comDE) have been detected in Sif- and Jordarchaeia MAGs. The gene encoding the enzyme catalyzing the final reaction using the substrate sulfoacetaldehyde to produce coenzyme M remains unknown.

Dihydromethanopterin reductase (acceptor) (dmrX) and 7,8-dihydropterin-6-yl-methyl-4- (beta-D-ribofuranosyl) aminobenzene 5'-phosphate synthase (EC:2.5.1.105) are two of the key enzymes in H_4_MPT biosynthesis pathway that are encoded in Sif- and Jordarchaeia MAGs. The gene dmrX encodes the enzyme catalysing the production of H_4_MPT together with dihydromethanopterin reductase (dmrA), the latter is absent in both lineages, however.

**Menaquinone**

Menaquinone is the presumed electron acceptor for electron transfer via the proposed, energy conserving Nuo (i.e., complex I) homolog. This conclusion is based on the fact that Sif- and Jordarchaeia encode the gene for demethylmenaquinone methyltransferase (ubiE), an enzyme that catalyses the biosynthesis of menaquinol with demethylmenaquinol. Menaquinols are the reduced form of menaquinones. Other genes in the menaquinone biosynthesis pathway present in Jordarchaeia MAGs include o-succinylbenzoate synthase (menC) and naphthoate synthase (menB). Two Sifarchaeia MAGs possess the genes for 1,4-dihydroxy-2-naphthoate polyprenyltransferase (menA). Sif- and Jordarchaeia also encode genes for enzymes synthesizing other cofactors such as coenzyme F420, NAD^+^, NADP^+^, and methanofuran. The annotations of these genes are listed in **Table S9** in the section ‘Biosynthesis of cofactors’.

**Bifurcating electron transfer flavoprotein (bf-ETF) and β-oxidation**

Sifarchaeia and Jordarchaeia possess genes which were annotated as fixA and fixB using Prokka and EnrichM (see Methods). These genes encode enzymes representing a subgroup of bifurcating electron transfer flavoproteins (bf-ETFs) which were found associated with nitrogen fixation and were therefore named ‘Fix’ [[7]](https://www.zotero.org/google-docs/?ON515w).

Both genes fixA and fixB have been described as part of the fixABCX operon which has been proposed to perform quinone‐mediated reverse electron transport from reduced ETF to a membrane‐bound hydrogenase for H_2_ generation [[8]](https://www.zotero.org/google-docs/?QYcQU5). However, Sif- and Jordarchaeia do not encode any of the remaining genes fixC and fixX in this operon .

Based on this finding and our analysis of the gene neighbourhood (**Suppl. Text Fig. 1a**), we propose that the fixAB genes are mis-annotated in Sif- and Jordarchaeia in are in fact electron bifurcating ETFs that are not involved in quinone reduction but rather associated with a substrate-specific dehydrogenase. A reaction scheme for such ETFs has been proposed previously for anaerobic fermentative Firmicutes [[9]](https://www.zotero.org/google-docs/?vWseeC), in which ETF-b accepts electrons from NADH and bifurcates them to Ferredoxin and to the FAD in ETF-a which then donates electrons to a Butyryl-CoA dehydrogenase to reduce crotonyl-CoA to butyryl-CoA. We propose a similar mechanism for Sif- and Jordarchaeia, where Acyl-CoA dehydrogenases reduce Acyl-CoA in the initial step of each cycle in the fatty acid β-oxidation (**Suppl. Text Fig. 1a**).


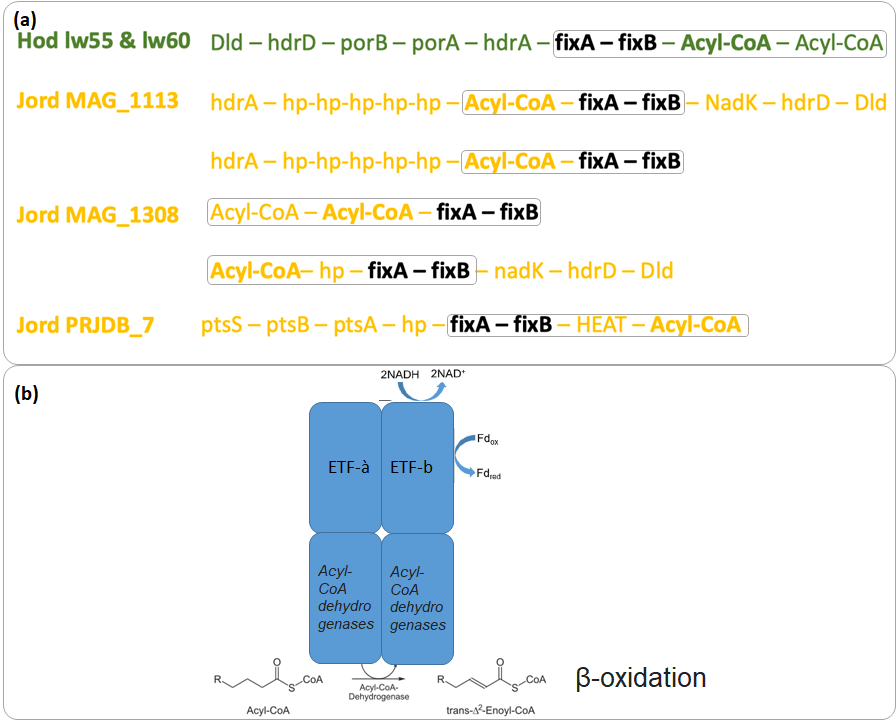


**Sif**

**Suppl. Text Fig. 1 | FixAB annotated ETF genes in Sif- and Jordarchaeia**. **(a)** Gene neighbourhood. Genes originally annotated as fixA and fixB, Acyl-CoA dehydrogenases (Acyl-CoA), and intermediate genes are highlighted with a light grey box. **(b)** Proposed ETF-dehydrogenase complex. ETF-b accepts electrons from NADH and bifurcates them to ferredoxin and to the FAD in ETF-a which then donates electrons to a Acyl-CoA dehydrogenases to reduce Acyl-CoA in the initial step of each cycle in the fatty acid β-oxidation.

**β-oxidation of fatty acids**

Sifarchaeia and Jordarchaeia encode a near complete β-oxidation pathway (**Suppl. Text Fig. 2**), including Acyl-CoA dehydrogenases (ACD) and Enoyl-CoA hydratase (ECH) [EC:4.2.1.17] . Genes for the enzyme Acetyl-CoA C-acyltransferase (alternative name Beta-ketothiolase) were not detected, however we found the gene K00626 (atoB; acetyl-CoA C-acetyltransferase [EC:2.3.1.9]) in both Sif- and Jordarchaeia with 3 to 19 copies per genome. It has been suggested that this enzyme is a likely component of the archaeal pathway of fatty acid metabolism, although it has been characterised as an enzyme of the mevalonate biosynthesis pathway [[10]](https://www.zotero.org/google-docs/?EfYOjA). Genes for 3-hydroxyacyl-CoA dehydrogenase (HDH) [EC:1.1.1.35] were absent from Sif- and Jordarchaeia genomes, similar to previous reports that HDH was not detected in several Crenarchaeota genomes [[10]](https://www.zotero.org/google-docs/?UGAzp8), which suggest that a so far uncharacterized enzyme might catalyse this reaction in some Archaea.

**
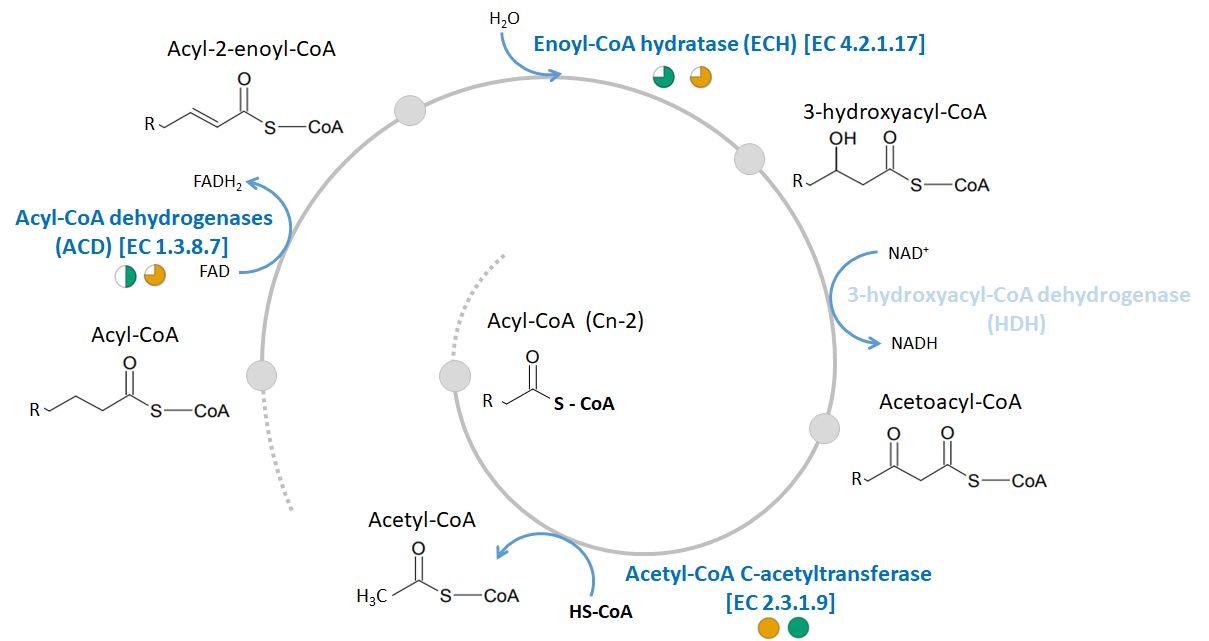
**

**Suppl. Text Fig. 2 | β-oxidation of fatty acids.** Genes present in Sifarchaeia genomes are indicated by green pie charts and genes present in Jordarchaeia by yellow pie charts. Note that each cycle reduces the fatty acid by two carbon atoms. Sketch is modified from [[10]](https://www.zotero.org/google-docs/?L73imj).

**Hydrogen and formate evolving enzymes and the link to lactate conversion**

Sif- and Jordarchaeia encode a HdrABC-MhvADG-NiFe-hydrogenase (**Fig. 2**). This enzyme could perform a function similar to the H₂ evolving electron-confurcating homolog reported for strain MK-D1 (Imachi et al. 2020). Thereby reduced ferrodoxin and CoM-CoB heterodisulfide (Com-SS-Cob) are oxidised, whereby Com-SS-Cob is converted to coenzyme B and coenzyme M which in turn catalyses the reduction of D-lactate to pyruvate via a putative DlD-HdrD complex (**Fig. 2**). The predicted D-lactate dehydrogenase (Dld) of Sifarchaeia is structurally similar to *Archaeoglobus fulgidus* homologs (**Fig. S15b,c**), contains previously proposed FAD-binding sites (**Fig. S15d**), clusters with other Dld sequences in our phylogenetic inference (**Fig. S15e**), and was found to be encoded next to a CoB--CoM heterodisulfide reductase subunit D (HdrD) gene in our gene neighborhood screen (**Fig. S15a, Table S20**). Therefore, we propose that the HdrABC-MhvADG-NiFe-hydrogenase complex in Asgardarchaeota is coupled with the conversion of D-lactate to pyruvate in Asgardarchaeota.

The Lokiarchaeia culture MK-D1 also encodes the electron transfer complex FixABCD–HdrABC associated with H_2_ based syntrophy [[11]](https://www.zotero.org/google-docs/?kLG3rc). This complex is not encoded in Sif- and Jordarchaeia genomes, instead both novel lineages encode a formate dehydrogenase which has been suggested to be involved in syntrophic interactions with sulfate reducing bacteria (SRB) [[12]](https://www.zotero.org/google-docs/?s7Xnbg). A possible SRB association of Sifarchaeia is further supported by our analysis of metadata, taxonomic and functional community profiles, and features inferred from genomic reconstructions (see main text).

**Selenocysteine-inserting tRNA (tRNAsec)**

We predicted Selenocysteine cognate tRNAs (tRNA-Sec) using the tool Secmarker [[13]](https://www.zotero.org/google-docs/?SNpeMW) (see Methods) which uses models that incorporate the structural features characteristic for tRNASec in each of the three proposed domains of life. The Asgardarchaeota tRNA-Sec sequences detected by Secmarker show these unique features: 1) they are considerably larger than canonical elongator tRNAs [[14]](https://www.zotero.org/google-docs/?HhbyAe), i.e. 86-142 nucleotides in Asgardarchaeota tRNAsec as compared to ~75 nucleotides in canonical tRNAs, 2) they possess a long variable arm [[14]](https://www.zotero.org/google-docs/?eVk97E), 3) they have a 13nt AT-stem which differs from the canonical 7/5 fold (i.e. 7 base pairs in the A-stem, and 5 base pairs in the T-stem) in other tRNAs [[13, 14]](https://www.zotero.org/google-docs/?alwLNH), and 4) they show a 4nt D-loop and a 6ntD-stem, in contrast to the 3–4 bp D-stem and 7–12 nt D-loop in the canonical tRNAs [[15–17]](https://www.zotero.org/google-docs/?wdJ1VM).

**References**

[1. Krzycki JA. Function of genetically encoded pyrrolysine in corrinoid-dependent methylamine methyltransferases. *Current Opinion in Chemical Biology* 2004; **8**: 484–491.](https://www.zotero.org/google-docs/?SdLC7H)

[2. Borrel G, Parisot N, Harris HM, Peyretaillade E, Gaci N, Tottey W, et al. Comparative genomics highlights the unique biology of Methanomassiliicoccales, a Thermoplasmatales-related seventh order of methanogenic archaea that encodes pyrrolysine. *BMC Genomics* 2014; **15**: 679.](https://www.zotero.org/google-docs/?SdLC7H)

[3. Ticak T, Kountz DJ, Girosky KE, Krzycki JA, Ferguson DJ. A nonpyrrolysine member of the widely distributed trimethylamine methyltransferase family is a glycine betaine methyltransferase. *PNAS* 2014; **111**: E4668–E4676.](https://www.zotero.org/google-docs/?SdLC7H)

[4. Upadhyay V, Ceh K, Tumulka F, Abele R, Hoffmann J, Langer J, et al. Molecular characterization of methanogenic N5-methyl-tetrahydromethanopterin: Coenzyme M methyltransferase. *Biochimica et Biophysica Acta (BBA) - Biomembranes* 2016; **1858**: 2140–2144.](https://www.zotero.org/google-docs/?SdLC7H)

[5. Wagner T, Ermler U, Shima S. MtrA of the sodium ion pumping methyltransferase binds cobalamin in a unique mode. *Scientific Reports* 2016; **6**: 28226.](https://www.zotero.org/google-docs/?SdLC7H)

[6. Speth DR, Orphan VJ. Metabolic marker gene mining provides insight in global mcrA diversity and, coupled with targeted genome reconstruction, sheds further light on metabolic potential of the Methanomassiliicoccales. *PeerJ* 2018; **6**.](https://www.zotero.org/google-docs/?SdLC7H)

[7. Mohamed-Raseek N, Duan HD, Hildebrandt P, Mroginski MA, Miller A-F. Spectroscopic, thermodynamic and computational evidence of the locations of the FADs in the nitrogen fixation-associated electron transfer flavoprotein. *Chem Sci* 2019; **10**: 7762–7772.](https://www.zotero.org/google-docs/?SdLC7H)

[8. Sieber JR, McInerney MJ, Gunsalus RP. Genomic Insights into Syntrophy: The Paradigm for Anaerobic Metabolic Cooperation. *Annual Review of Microbiology* 2012; **66**: 429–452.](https://www.zotero.org/google-docs/?SdLC7H)

[9. Costas AMG, Poudel S, Miller A-F, Schut GJ, Ledbetter RN, Fixen KR, et al. Defining Electron Bifurcation in the Electron-Transferring Flavoprotein Family. *Journal of Bacteriology* 2017; **199**.](https://www.zotero.org/google-docs/?SdLC7H)

[10. Dibrova DV, Galperin MY, Mulkidjanian AY. Phylogenomic reconstruction of archaeal fatty acid metabolism. *Environ Microbiol* 2014; **16**: 907–918.](https://www.zotero.org/google-docs/?SdLC7H)

[11. Nobu MK, Narihiro T, Hideyuki T, Qiu Y-L, Sekiguchi Y, Woyke T, et al. The genome of Syntrophorhabdus aromaticivorans strain UI provides new insights for syntrophic aromatic compound metabolism and electron flow. *Environmental Microbiology* 2015; **17**: 4861–4872.](https://www.zotero.org/google-docs/?SdLC7H)

[12. Imachi H, Nobu MK, Nakahara N, Morono Y, Ogawara M, Takaki Y, et al. Isolation of an archaeon at the prokaryote–eukaryote interface. *Nature* 2020; **577**: 519–525.](https://www.zotero.org/google-docs/?SdLC7H)

[13. Santesmasses D, Mariotti M, Guigó R. Computational identification of the selenocysteine tRNA (tRNASec) in genomes. *PLoS Comput Biol* 2017; **13**.](https://www.zotero.org/google-docs/?SdLC7H)

[14. Commans S, Böck A. Selenocysteine inserting tRNAs: an overview. *FEMS Microbiol Rev* 1999; **23**: 335–351.](https://www.zotero.org/google-docs/?SdLC7H)

[15. Sherrer RL, Araiso Y, Aldag C, Ishitani R, Ho JML, Söll D, et al. C-terminal domain of archaeal O-phosphoseryl-tRNA kinase displays large-scale motion to bind the 7-bp D-stem of archaeal tRNASec. *Nucleic Acids Res* 2011; **39**: 1034–1041.](https://www.zotero.org/google-docs/?SdLC7H)

[16. Mariotti M, Lobanov AV, Manta B, Santesmasses D, Bofill A, Guigó R, et al. Lokiarchaeota Marks the Transition between the Archaeal and Eukaryotic Selenocysteine Encoding Systems. *Mol Biol Evol* 2016; **33**: 2441–2453.](https://www.zotero.org/google-docs/?SdLC7H)

[17. Yuan J, O’Donoghue P, Ambrogelly A, Gundllapalli S, Sherrer RL, Palioura S, et al. Distinct genetic code expansion strategies for selenocysteine and pyrrolysine are reflected in different aminoacyl-tRNA formation systems. *FEBS Lett* 2010; **584**: 342–349.](https://www.zotero.org/google-docs/?SdLC7H)
